# Charge-Engineered LPS-Targeting Magnetic Nano-adsorbents with Optimized Harvesting Strategy Advance Sepsis Blood Purification Nanotherapeutic

**DOI:** 10.34133/research.0991

**Published:** 2025-12-08

**Authors:** Xianda Liu, Shengjun Cheng, Xijing Yang, Yilin Wang, Shifan Chen, Ziyue Ling, Yujie Xiao, Weifeng Zhao, Changsheng Zhao

**Affiliations:** ^1^ College of Polymer Science and Engineering, State Key Laboratory of Advanced Polymer Materials, Sichuan University, Chengdu, China.; ^2^ The Experimental Animal Center of West China Hospital, Sichuan University, Chengdu, China.; ^3^ Med-X Center for Materials, Sichuan University, Chengdu, China.

## Abstract

This study overcomes 2 critical barriers to the clinical translation of nano-adsorbents for sepsis blood purification: the weakening of adsorption function caused by nanoparticle biofouling and the limitations in clinical translation of recovery devices. We pioneer electrically neutral phosphocholine zwitterions as selective lipopolysaccharide (LPS) ligands. Their precise charge orientation resolves the core conflict between anti-fouling efficacy and LPS capture via differential dipole realignment upon LPS binding, enabling unprecedented selective LPS capture capacity with minimal protein adsorption. To address the persistent challenges of nano-adsorbent retrieval from blood, and clinical incompatibility of existing retrieval devices with blood purification systems, we developed a discretely assembled magnetic nanocomposite platform (PCAPAN-Fe) and an extracorporeal LPS-targeting magnetic array system (ELMAS), eliminating key risks inherent in monolithic designs while ensuring complete nanoparticle harvest. In septic rabbit models, the integrated platform exhibited 100% survival with early intervention, 84.7% LPS clearance (versus 20% survival and 45.6% LPS clearance for commercial adsorbents), and marked reduction of key proinflammatory cytokines. Crucially, the therapy achieved 82.2% LPS clearance efficacy in progressive sepsis, extending survival to 40% (versus 0% for commercial adsorbents). By ingeniously integrating molecular-level ligand design with a clinically viable device, this work pioneers a paradigm shifts in sepsis nanotherapeutic, resolving the performance–biosafety paradox in blood purification.

## Introduction

Sepsis is a systemic disease due to microbial invasion that mediates dysregulation of the host’s immune response, ultimately leading to acute organ dysfunction and a high risk of death [1]. As of 2019, about 13.9 million people worldwide died from infectious syndromes related to sepsis [2]. In China, approximately 1 million deaths were associated with sepsis from 2017 to 2019 [3]. Additionally, hospitalized sepsis and in-hospital mortality continue to rise in China, which signals the intensifying pressures on the healthcare system. Because of the high morbidity, lethality, and treatment cost, sepsis has become one of the significant threats to public health in the world [4].

Lipopolysaccharide (LPS), also known as endotoxin, produced by Gram-negative bacteria is considered one of the causative agents of sepsis [5]. LPSs possess a substantial number of phosphate residues, giving them a negative charge [6]. In the bloodstream, LPS could interact with the immune cell membrane and signal through Toll-like receptor 4–myeloid differentiation factor 2 (TLR4-MD2) complexes, triggering a cytokine storm and leading to irreversible organ damage. [7]. Thus, sepsis progresses with remarkable rapidity, and its treatment necessitates urgent intervention within a critical time window.

The early removal of LPS from the blood could potentially mitigate the onset of an uncontrolled pro-inflammatory reaction, thereby enhancing the prognosis for septic patients [8,9]. Removing LPS from septic blood by extracorporeal hemoperfusion has turned into a crucial therapeutic approach for managing sepsis [10]. Various cartridges have been developed for the removal of LPS in sepsis treatment. Unfortunately, the current resin-based LPS adsorbents often do not achieve the desired therapeutic outcomes [11–13]. The limited adsorption capacity and kinetic performance of existing resin-based LPS adsorbents are insufficient to effectively inhibit the LPS-induced cascade inflammatory response. Recent advances in LPS adsorbent development have focused on utilizing ligands with a higher binding affinity for LPS, such as polyethyleneimine (PEI) [14], quaternary ammonium [15], imidazole [16], and polypeptide [17]. While increasing ligand graft density and binding strength on resin-based materials improves LPS adsorption capacity and kinetics, it also amplifies nonspecific interactions with plasma proteins, compromising efficacy.

In recent years, numerous research teams have developed various nano-adsorbents aimed at application in sepsis blood purification [18–20]. Owing to their ultra-high specific surface area, these nano-adsorbents can significantly enhance the clearance rate and adsorption capacity for LPS, thereby substantially overcoming the limitations inherent in traditional resin adsorbents. However, considerable caution and significant concerns persist within the scientific community regarding the deployment of these nano-adsorbents in blood purification. Given the complex protein milieu of blood, a protein corona forms on the nano-adsorbent’s surface within seconds of blood contact [21]. This corona not only masks the functional sites on the nano-adsorbents surface but also provides docking sites for fibrinogen (FIB) and coagulation factors. Consequently, this phenomenon can induce particle aggregation, platelet activation, thrombogenesis, and inflammatory responses [22]. Furthermore, the nonspecific protein adsorption occurring on nano-adsorbents in the bloodstream compromises their intended adsorption efficacy and concurrently poses substantial hemotoxicity.

Another critical challenge limiting the application of nano-adsorbents in sepsis blood purification is the direct cytotoxicity induced by nanoparticle leaching following systemic administration, alongside cumulative toxicity arising from organ uptake [23]. A prevalent strategy to address this issue employs maneuverable magnetic nanoparticles (MNPs) [24]. In this approach, blood is extracorporeally circulated, mixed with MNPs for LPS adsorption, and subsequently passed through a magnetic retrieval unit designed to achieve complete MNP separation before blood reentry into the body. Notably, although various magnetic recovery systems for MNPs have been developed, these retrieval units predominantly rely on multi-channel configurations fabricated by precision etching within microfluidic modules [25–27]. This design paradigm entails complex manufacturing processes, elevated costs, and significant challenges in sterilizing the integrated architecture. Furthermore, effective removal of air bubbles within the confined microchannels during blood purification procedures is problematic, posing a substantial risk of thrombogenesis. The current suboptimal design of extracorporeal retrieval systems significantly impedes the broader implementation of MNPs in blood purification modalities.

Overall, the primary challenges limiting the application of nano-adsorbents in sepsis blood purification therapy stem from the inherent conflict between nonspecific protein adsorption and the selective adsorption of LPS, coupled with the engineering difficulties associated with blood separation devices. To address these limitations, we propose a novel strategy utilizing nano-adsorbents for sepsis treatment via blood purification. Firstly, targeting the fundamental challenge of reducing thermodynamic competition while enhancing LPS selectivity, we introduce a theoretical concept: employing a dynamically polarizable zwitterionic phosphocholine (PC) as the LPS-adsorbing moiety. Under whole blood conditions, the zwitterion exhibits a zero net electrostatic potential (ESP), forming a hydration layer that effectively resists protein fouling [28,29]. Upon approach of negatively charged LPS, polarization of the choline cation center within the zwitterion occurs, enabling specific LPS adsorption. This mechanism thereby concurrently achieves protein fouling resistance and high-efficiency LPS clearance. To our knowledge, this represents the first proposal of a simple, electrically neutral ligand serving as a selective LPS adsorbent. We discuss the relationship between the charge orientation of the PC zwitterion and LPS adsorption, elucidate the underlying binding mechanism, and provide a comprehensive, detailed comparison with traditional quaternary ammonium adsorbent ligands in terms of efficacy and safety profile.

Addressing the nanoparticle separation challenge, we engineered a discretely assembled magnetic nanocomposite characterized by smaller superparamagnetic nanoparticles uniformly immobilized on larger carrier nanoparticles, exhibiting a “blueberry muffin” morphology. This architecture enhances magnetic responsivity while ensuring colloidal stability in biological fluids. Departing from conventional monolithic separation systems, we developed a modular magnetic retrieval system for extracorporeal circuits. The system employs optimized magnet array configurations to generate amplified high-gradient magnetic fields, enabling efficient nanoparticle capture under physiological shear conditions. The modular architecture resolves critical limitations of integrated systems through detachable component processing and flexible standalone circuit design. This approach specifically addresses sterilization complexity and bubble entrapment risks. This design demonstrates superior clinical adaptability and translational feasibility compared to existing retrievable magnetic adsorbent platforms.

Collectively, this study pioneers an exceptionally simple method for achieving superior LPS selectivity within the complex blood environment. This is accomplished solely by designing a zwitterionic structure that leverages its differential polarization behavior under varying ionic conditions. This technology fundamentally resolves the persistent thermodynamic competition challenge between LPS and plasma proteins in blood purification. Through synergistic engineering of nano-scale optimization strategies and a magnetic separation system, we concurrently address the critical issues of nano-adsorbents leakage and organ retention-induced in vivo toxicity. This establishes a translational solution framework for the safe application of nanomaterials in extracorporeal therapies. The research provides a paradigm-shifting technological pathway for designing next-generation sepsis blood purification nanoplatforms.

The contributions of this work can be briefly summarized as follows:

1. We first demonstrate optimal charge-oriented zwitterionic nano-adsorbent, elucidating their mechanism for selective LPS adsorption in blood.
2. The nano-adsorbent (PCAPAN-Fe) delivered ~1,000-fold higher LPS adsorption capacity than reported resin-based adsorbents while maintaining ~^1^/_10_ nonspecific protein adsorption of other nano-adsorbents (e.g., nanofibers).
3. Innovative nano-adsorbent recovery system (ELMAS) overcomes operational limitations of previous counterparts, enabling clinical blood purification translation of nano-adsorbents.
4. Early intervention using the ELMAS device loaded with PCAPAN-Fe achieved a 100% 28-d survival rate in septic rabbits, with a superior prognosis compared to commercial resin adsorbents.

## Results and Discussion

### Design and characterization of the LPS-targeting MNPs

The design of LPS-targeting MNPs exploits differential polarization responses of PC zwitterions in distinct electrostatic environments: maintaining charge homeostasis to resist plasma protein adsorption while undergoing field-induced dipole realignment for selective LPS capture. Despite exhibiting net electroneutrality [30], PC fundamentally differs from nonionic materials through localized anionic (phosphate) and cationic (choline) charge centers. This zwitterionic architecture confers protein fouling resistance via hydration effects while retaining inducible adsorption capacity under strong dipole fields, which is a phenomenon driven by dipole–dipole complementarity through oriented alignment [31]. We therefore hypothesize that PC zwitterions with rationally designed dipole orientations can function as dual anti-fouling and LPS-binding ligands via charge-complementary molecular recognition.

**Fig. 1.**
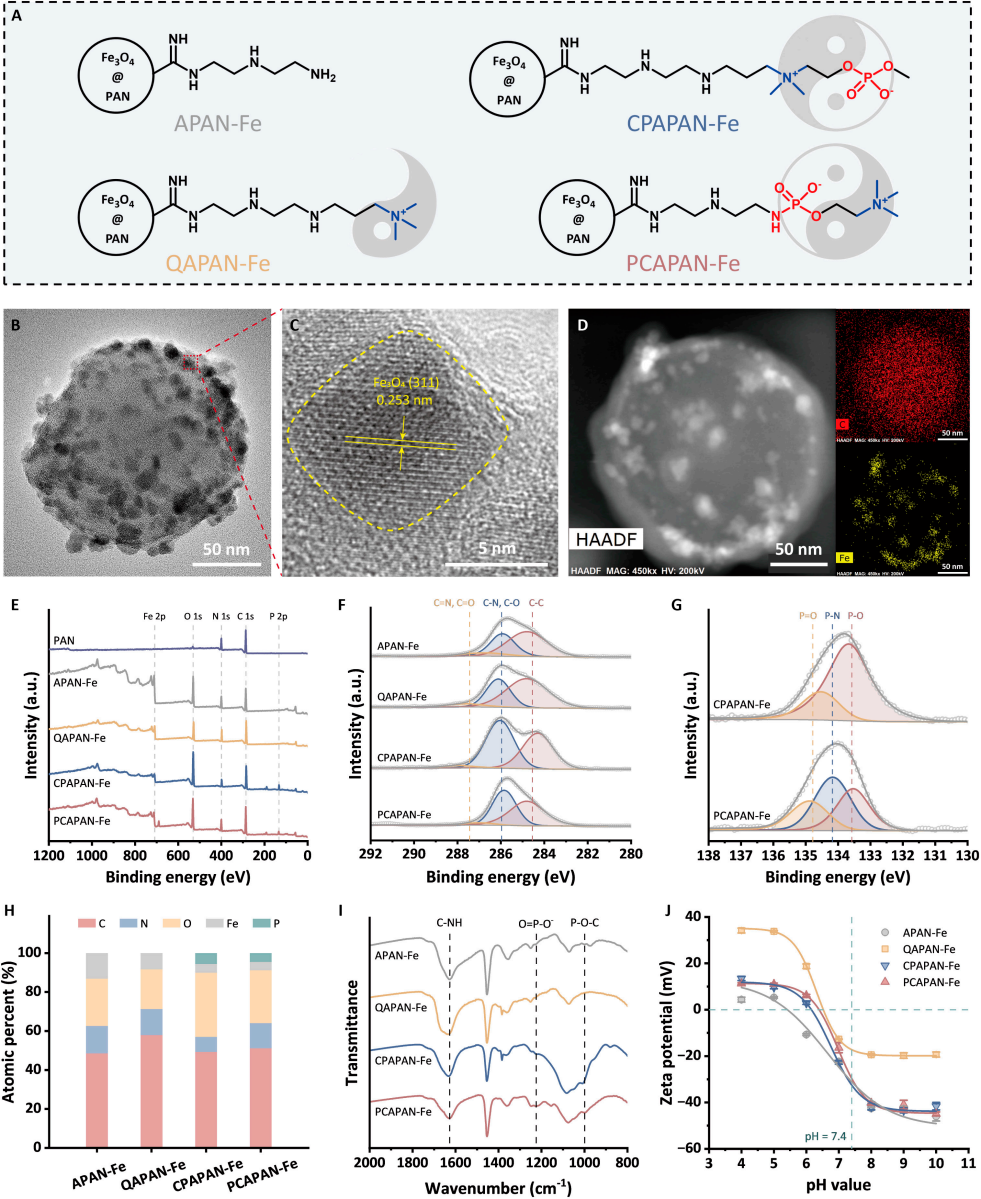
Ligand chemical structures on MNPs and characterization of their morphology and composition. (A) Chemical structures of MNPs. (B) TEM image and (C) HRTEM image of PCAPAN-Fe. (D) High-angle annular dark field image (HAADF) image and C, Fe species mapping of PCAPAN-Fe. (E) XPS survey scans of PAN and MNPs. (F) C 1s and (G) P 2p spectra of MNPs. (H) Elemental composition of the MNP surface. (I) FTIR spectra of MNPs. (J) Zeta potential under different pH values of MNPs.

To validate this hypothesis, 4 ligand structures were designed (Fig. 1A). The amino-functionalized MNP (APAN-Fe) with weak charges was used as the nanoplatform, onto which 2 distinct zwitterionic structures with different electric dipole orientations were grafted. This resulted in the formation of CPAPAN-Fe (with a positive-to-negative dipole orientation) and PCAPAN-Fe (with a negative-to-positive dipole orientation). Additionally, a quaternary ammonium-functionalized MNP (QAPAN-Fe) was synthesized as a control, featuring a strong positive charge, since strong positive charges were widely regarded as a conventional strategy for designing LPS adsorbents. MNPs were fabricated as a “blueberry muffin”-like discretely assembled magnetic nanocomposite. The preparation route involved in situ deposition of Fe_3_O_4_ onto microemulsion-polymerized polyacrylonitrile (PAN) [32], followed by grafting of PC, choline phosphate (CP), or quaternary ammonium groups (the preparation processes were shown in Figs. S1 and S2).

Transmission electron microscopy (TEM) images revealed the composite “blueberry muffin” structure (Fig. 1B and Fig. S3). High-resolution TEM (HRTEM) image (Fig. 1C) distinguished the Fe_3_O_4_–polymer interface, with Fe_3_O_4_ lattice spacing at 0.253 nm [(311) plane] [33]. The energy-dispersive x-ray spectroscopy (EDS) mapping (Fig. 1D and Fig. S4) showed homogeneous carbon distribution and clustered Fe in island patterns, confirming uniform Fe_3_O_4_ dispersion on the polymer substrate.

X-ray photoelectron spectroscopy (XPS) detected Fe, O, N, C, and P (Fig. 1E). Post-grafting, P emerged in CPAPAN-Fe and PCAPAN-Fe. Deconvoluted C 1s spectra (Fig. 1F) showed peaks for C–C (~284.8 eV), C–N/C–O (~285.9 eV), and C=N/C=O (~286.7 eV). N 1s spectra (Fig. S5) indicated C–NH/C=N (~399.1 eV) from the polymer backbone and C–N^+^ (~401.2 eV) in QAPAN-Fe, CPAPAN-Fe, and PCAPAN-Fe, confirming quaternary ammonium/chlorine incorporation [34]. P 2p spectra (Fig. 1G) displayed P–O (~133.5 eV) and P=O (~134.7 eV) in CPAPAN-Fe [35], while PCAPAN-Fe showed P–N (~134.2 eV) from PC–amino group grafting. Based on the calculation of surface atomic percentage ratios (Fig. 1H), the functional group grafting ratios for QAPAN-Fe, CPAPAN-Fe, and PCAPAN-Fe were determined to be 7.07, 16.67, and 13.77 mol %, respectively.

The Fourier transform infrared spectroscopy (FTIR) spectra revealed N–H bending (1,630 cm^−1^) and new peaks at 1,219 cm^−1^ (P–O stretch) and 997 cm^−1^ (P–O–C stretch) in CPAPAN-Fe and PCAPAN-Fe [36] (Fig. 1I, peak assignments in Table S1). Zeta potentials (Fig. 1J) showed QAPAN-Fe strongly positive due to dense quaternary ammonium charge. At low pH, CPAPAN-Fe and PCAPAN-Fe exhibited slightly higher potentials than APAN-Fe, likely from phosphate protonation exposing cationic choline [37]. Minimal differences at pH 7.4 reflected zwitterionic charge balance.

### Thermodynamic and kinetic mechanisms of LPS binding to the LPS-targeting MNPs

**Fig. 2.**
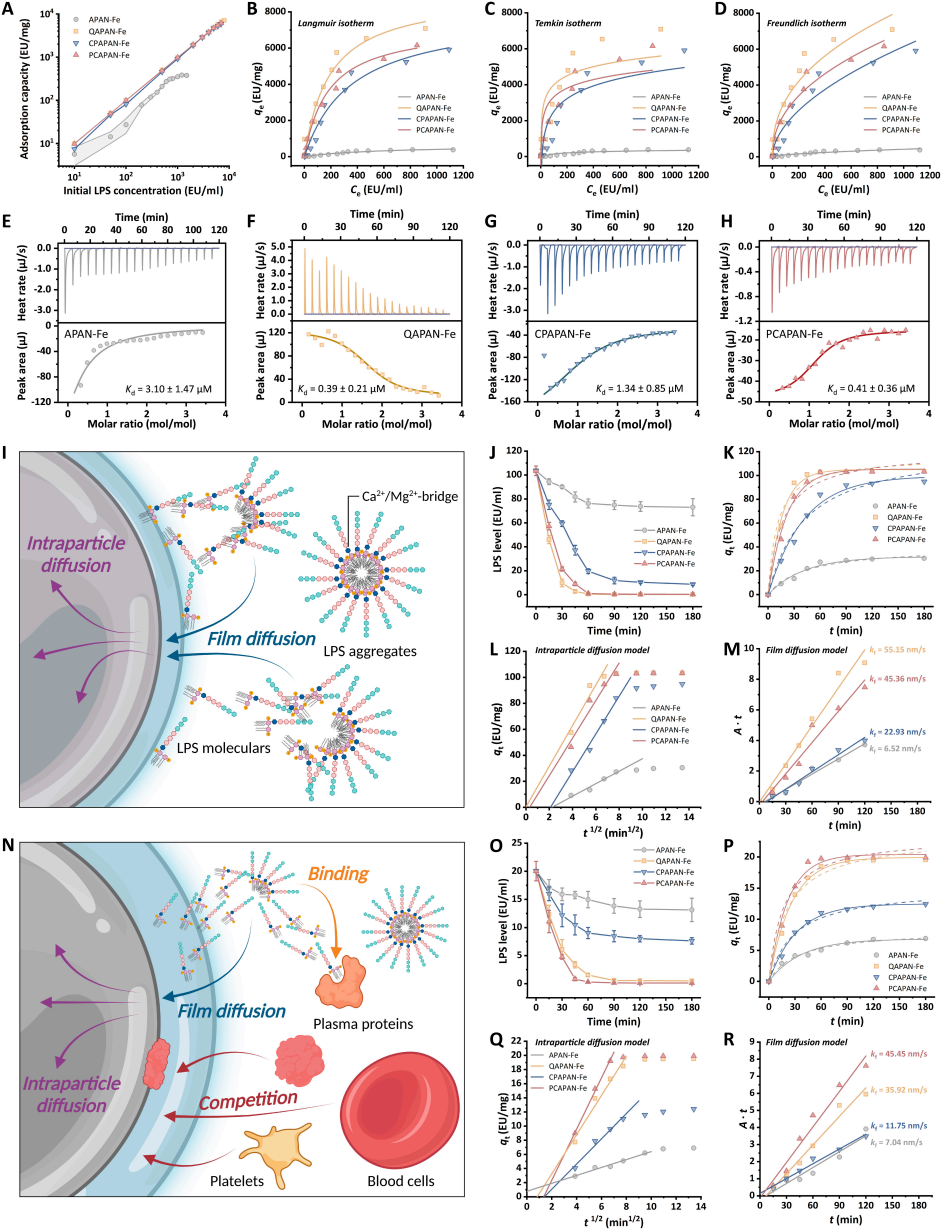
Thermodynamic and kinetic investigations of LPS adsorption by MNPs in buffer and blood environment. (A) Equilibrium adsorption capacity of MNPs for solutions with different initial LPS concentrations. (B) Langmuir isotherm, (C) Temkin isotherm, and (D) Freundlich isotherm fitted image of MNPs. (E) ITC titration and fitted curves of APAN-Fe, (F) QAPAN-Fe, (G) CPAPAN-Fe, and (H) PCAPAN-Fe. (I) Schematic illustration of LPS adsorption mechanism by MNPs in DPBS buffer. (J) Time-dependent level changes of LPS treated by MNPs in DPBS buffer. (K) Fitting plots of PFO/PSO adsorption kinetic models, (L) intraparticle diffusion model, and (M) film diffusion model in DPBS buffer. (N) Schematic illustration of LPS adsorption mechanism by MNPs in septic blood. (O) Time-dependent level changes of LPS treated by MNPs in human whole blood. (P) Fitting plots of PFO/PSO adsorption kinetic models, (Q) intraparticle diffusion model, and (R) film diffusion model in human whole blood.

To conduct a systematic study of the LPS adsorption thermodynamic and kinetic mechanisms of MNPs, we first assessed their adsorption capacity at 37 °C. Compared to APAN-Fe, the adsorption capacities of QAPAN-Fe, CPAPAN-Fe, and PCAPAN-Fe were notably higher at gradient initial LPS concentrations (Fig. 2A). We characterized this adsorption process using adsorption isotherms. The corresponding fitting parameters were listed in Table S2. The Langmuir isotherm was employed to describe monolayer adsorption on an ideal homogeneous surface [38]. The Langmuir isotherm demonstrated a good fit to the adsorption data (Fig. 2B). Compared to APAN-Fe, the *q*_max_ values of PCAPAN-Fe and CPAPAN-Fe were significantly increased, with CPAPAN-Fe having a close *q*_max_ than PCAPAN-Fe [7,986.35 versus 7,275.23 endotoxin units (EU)/mg]. It was indicated that at adsorption equilibrium, the binding sites of both PC and CP groups were effectively occupied by LPS.

The Temkin and Freundlich isotherms were used to model multilayer adsorption on heterogeneous surfaces [38]. The key distinction was that the Temkin isotherm assumed a uniform distribution of binding energies across adsorption sites, with the binding energy decreasing linearly as sites are occupied [39]. In contrast, the Freundlich isotherm assumed an exponential decrease in binding energy as the adsorption sites become occupied [40]. The Freundlich isotherm provided a better fit to the data (Fig. 2C and D). Due to the complex surface and interface behavior of LPS in solution, a single simple adsorption isotherm was inadequate for accurately describing the adsorption process of MNPs on LPS. By comparing the fitting results of various isotherm models, we inferred that LPS is likely adsorbed onto MNPs in a manner approaching a monolayer, which can be attributed to steric hindrance effects resulting from the large molecular size of LPS. As the adsorption sites were occupied, the binding energy decreased nonlinearly.

To further study the thermodynamic process of MNPs binding with LPS, isothermal titration calorimetry (ITC) was employed to investigate the differences in binding affinity. The parameter fitting results were listed in Table S3. The Gibbs free energies (*ΔG*) for all MNPs were <0, proving that the binding of MNPs to LPS tended to proceed spontaneously. APAN-Fe exhibited a weak affinity for LPS (Fig. 2E), with a dissociation constant (*K*_d_) of 3.10 μM. The binding of QAPAN-Fe to LPS was an endothermic, entropy-driven process (Fig. 2F), resulting from the release of counterions and bound water when quaternary ammonium groups interact with polyanions [41,42].

However, the zwitterion-modified MNPs exhibited an enthalpy-driven adsorption process for LPS (Fig. 2G and H), characterized by exothermic and entropy-decreasing interactions, in stark contrast to QAPAN-Fe. According to established theories, the binding process between MNPs and LPS involved both the release of counterions and bound water, and the migration of LPS to MNPs [43]. The release of counterions and bound water was an entropy-driven process, whereas the migration of LPS was enthalpy-driven. At lower temperatures (37 °C), the binding of QAPAN-Fe to LPS was predominantly governed by entropy. For the zwitterion-rich PCAPAN-Fe and CPAPAN-Fe, their strong water-retention capacity diminished the release of counterions and water, thereby making the adsorption process predominantly enthalpy-driven due to LPS migration. The order of binding capacities for the 4 types of MNPs was QAPAN-Fe > PCAPAN-Fe > CPAPAN-Fe > APAN-Fe. QAPAN-Fe exhibited the minimum *K*_d_ for LPS (0.39 μM), attributed to its high density of cationic groups. However, due to the different orientations of zwitterionic charge centers, CPAPAN-Fe exposes negative charge centers, while PCAPAN-Fe exposes positive ones. As a result, CPAPAN-Fe requires more energy for conformational adjustment to bind with LPS, leading to greater entropic hindrance. Therefore, PCAPAN-Fe demonstrated a significantly lower *K*_d_ for LPS compared to CPAPAN-Fe (0.41 versus 1.34 μM), indicating a higher affinity of the PC group for LPS.

Next, we conducted a systematic investigation into the adsorption kinetics of LPS by MNPs. Initially, Dulbecco’s phosphate-buffered saline (DPBS) with Ca^2+^ and Mg^2+^ was selected as the buffer system to simulate physiological conditions. The LPS adsorption process by MNPs can be divided into 2 distinct stages: film diffusion and intraparticle diffusion (Fig. 2I). Film diffusion referred to the migration of LPS molecules across the liquid–solid interface to the MNP surface [44], while intraparticle diffusion described the subsequent penetration of surface-bound LPS into the micropores of MNPs [45]. In Ca^2+^- and Mg^2+^-containing solutions, LPS molecules tend to form bridged micelles [46], thereby increasing resistance during the film diffusion stage. QAPAN-Fe and PCAPAN-Fe exhibited superior adsorption performance compared to CPAPAN-Fe (Fig. 2J), which can be attributed to their significantly lower *K*_d_ for LPS, resulting in stronger driving forces for liquid film diffusion. The adsorption kinetics were analyzed using pseudo-first-order (PFO) and pseudo-second-order (PSO) models, with corresponding parameters summarized in Table S4. The adsorption kinetics of LPS by MNPs in DPBS are better described by the PFO model (Fig. 2K). This phenomenon can likely be attributed to the substantial molecular dimensions of LPS, which renders its adsorption process predominantly governed by mass transport limitations during migration to the adsorbent surface, rather than being primarily dictated by the intrinsic adsorption kinetics at the active sites.

To further elucidate the distinct contributions of film diffusion and intraparticle diffusion to the adsorption process, we conducted complementary mechanistic analyses. The Weber & Morris (W&M) model was employed to characterize intraparticle diffusion dynamics. This model proposed that when the plot of *q*_t_ versus *t*^1/2^ yields a linear relationship passing through the origin during the initial adsorption phase, intraparticle diffusion serves as the rate-limiting step [47]. Only QAPAN-Fe and PCAPAN-Fe exhibited linear fits intersecting the origin (Fig. 2L), indicating that their LPS adsorption processes were governed by intraparticle diffusion.

Concurrently, to investigate the film diffusion dynamics during LPS adsorption by MNPs, we applied a dynamic model developed by Yao and Chen [48], which coupled thin-film mass transfer theory with Langmuir equilibrium principles. According to this model, adsorption processes dominated by film diffusion control demonstrate linear correlations passing through the origin, with slope magnitudes reflecting film diffusion rates. The 4 MNPs exhibited origin-intersecting linear fits (Fig. 2M), suggesting universal film diffusion control across these systems. This dichotomy in rate-limiting mechanisms can be rationalized through interfacial energetics: APAN-Fe and CPAPAN-Fe demonstrated weaker LPS-binding affinity, resulting in insufficient driving forces for rapid surface migration. Consequently, their adsorption kinetics remained governed by film diffusion limitations. In contrast, QAPAN-Fe and PCAPAN-Fe exhibited stronger LPS-binding energies, enabling efficient interfacial transport that shifted the rate control to a combined film/intraparticle diffusion regime. In DPBS, QAPAN-Fe demonstrated significantly enhanced film diffusion rate constant (*k*_f_) for LPS compared to PCAPAN-Fe (55.15 versus 45.36 nm/s), a phenomenon potentially attributable to its superior surface charge characteristics that facilitate electrostatic-driven interfacial transport.

The adsorption dynamics in whole blood environments exhibited significantly greater complexity compared to buffer systems. Plasma proteins and blood cells competitively occupied adsorption sites on MNPs with LPS, while LPS-binding plasma proteins further competed with MNPs for LPS complexation (Fig. 2N). Time-dependent LPS removal profiles in human whole blood revealed comparable elimination efficiencies between QAPAN-Fe and PCAPAN-Fe (Fig. 2O), both demonstrating statistically superior performance over APAN-Fe and CPAPAN-Fe. Consistent with observations in DPBS, the adsorption kinetics remained describable by the PFO model (Fig. 2P and Table S5).

To rationalize the intricate adsorption kinetics in blood, we attribute the process to dual constraints on interfacial transport: (a) enhanced film diffusion resistance from competitive adsorption by blood components and (b) reduced intraparticle diffusion rates due to protein fouling within MNP’s micropores. The W&M model analysis (Fig. 2Q) showed non-origin-intersecting fitting lines for all 4 MNPs, contrasting with the origin-passing film diffusion model plots (Fig. 2R). This dichotomy confirmed film diffusion as the dominant rate-limiting step in whole blood. Upon contact with blood, MNPs immediately form a protein corona on their surface. The inner layer, known as the hard protein corona, consists of tightly bound proteins that cannot exchange with solutes, while the outer layer, referred to as the soft protein corona, is dynamic and allows exchange with solutes [49]. The formation of the hard protein corona could hinder the interaction between functional groups on the MNP surface and LPS, whereas the soft protein corona enhanced the competition for adsorption between blood components and LPS. Together, these effects contribute to the increased resistance in liquid film diffusion. Mechanistically, competitive adsorption and protein fouling collectively impede LPS migration to MNP surfaces, thereby amplifying the kinetic control exerted by liquid film transport.

Although both QAPAN-Fe and PCAPAN-Fe effectively adsorbed LPS in DPBS and human whole blood, comparative analysis of their *k*_f_ revealed distinct environmental dependencies. QAPAN-Fe exhibited a significantly lower *k*_f_ in blood compared to DPBS (35.92 versus 55.15 nm/s), whereas blood conditions cause no notable reduction in the *k*_f_ of PCAPAN-Fe (45.45 versus 45.36 nm/s). Indeed, the *k*_f_ of PCAPAN-Fe in blood even exceeded that of QAPAN-Fe. This suggested that competitive interactions and fouling by blood components impaired adsorption performance of QAPAN-Fe by introducing substantial mass transfer resistance for LPS. In contrast, zwitterionic structures of PCAPAN-Fe mitigated interactions with proteins and blood cells while preserving the efficient combination of LPS, thereby preventing interference from blood components in the LPS adsorption process. Consequently, PCAPAN-Fe retained superior adsorption capacity in blood environments.

### Computer simulation of binding processes between PC-functionalized surfaces and LPS

**Fig. 3.**
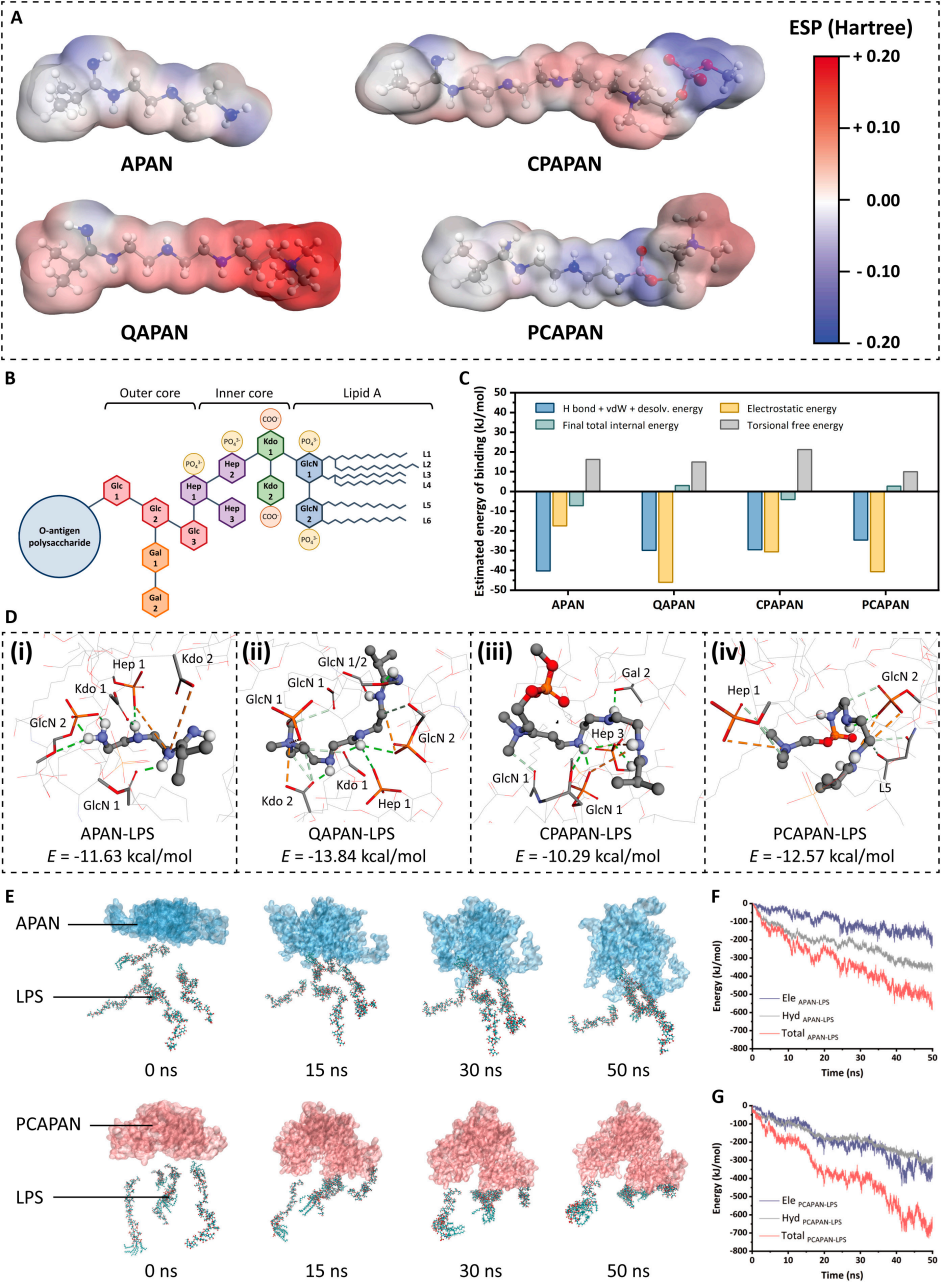
Computational modeling of interactions between ligand structures of MNPs and LPS. (A) Electrostatic potential surface (EPS) maps of MNPs (C, gray; H, white; O, red; N, blue; P, pink). (B) Encoding of sugar residues in the core oligosaccharide and lipid A of *E. coli* LPS (Glc, d-glucose; Gal, d-galactose; Hep, l-glycero-d-manno heptose; Kdo, 2-keto-3-deoxyoctulosonate; GlcN, d-glucosamine). (C) Estimated energy of the complex systems formed by the organic parts of various MNPs with LPS in molecular docking simulations. (D) Visualization of the lowest-energy conformations and interaction binding sites from molecular docking simulations of (i) APAN, (ii) QAPAN, (iii) CPAPAN, and (iv) PCAPAN binding LPS complex systems. (E) MD simulation of the interaction of the LPS with MNPs. The changes of interaction energy between (F) APAN or (G) PCAPAN and LPS during the simulation.

In vitro adsorption experiments, we found that PCAPAN-Fe exhibited stronger adsorption capacity for LPS compared to CPAPAN-Fe. Building on this foundation, we sought to investigate the relationship between zwitterionic dipole orientation and LPS-binding interactions. We initially calculated the surface ESP distributions of 4 ligand molecules (Fig. 3A). APAN exhibited minimal charge across its molecular surface. Notably, QAPAN demonstrated strong molecular polarization due to the inherent positive charge of its quaternary ammonium groups, forming a localized positive charge center. In contrast, CPAPAN and PCAPAN displayed opposing charge orientations. Their choline and phosphate moieties exhibited equimolar positive and negative charges, respectively, resulting in net charge neutralization.

To further explore these mechanisms, we employed computational simulation methods to investigate the binding interactions between the organic components of various MNPs and LPS. LPS is a negatively charged polysaccharide, with anionic groups such as carboxyl and phosphate groups predominantly located in the core oligosaccharide region. Following the previous methodology of Erridge et al. [50] and Wu et al. [51], the sugar residues of the LPS core oligosaccharide from *Escherichia coli* (one of the most common bacteria in sepsis) were encoded to facilitate subsequent studies (Fig. 3B).

To investigate equilibrium binding interactions between MNPs and LPS, we performed global flexible molecular docking to quantify binding energies of various MNP organic components with LPS core oligosaccharide and lipid A domains. AutoDock-derived scoring (Fig. 3C) revealed 4 dominant interaction types governing these complexes. While APAN primarily utilized hydrogen bonding and van der Waals forces through diethylenetriamine (DETA) side-chain amino/imino groups (Fig. 3D), quaternary ammonium-modified QAPAN exhibited enhanced electrostatic interactions targeting Kdo2 carboxyl and GlcN1 phosphate groups. In contrast, CPAPAN maintained electrostatic binding primarily via imino groups due to ineffective CP group interactions, whereas PCAPAN demonstrated superior phosphate group engagement at Hep1 through reversed electrostatic orientation of PC moieties. This mechanistic distinction explains the greater stability of the PCAPAN–LPS complexes (−12.57 kcal/mol) versus CPAPAN–LPS (−10.29 kcal/mol).

The binding of LPS to the PC group has been overlooked in previous studies, presenting a novel and understudied phenomenon.

To understand the molecular mechanism of the PC group’s interaction with LPS, molecular dynamics (MD) simulations were employed to study the binding process between the complete LPS molecule and PCAPAN. For comparison, we also simulated the interaction of LPS with APAN lacking grafted PC. LPS molecules rapidly approached the PCAPAN molecular chain, and as the simulation time was prolonged, LPS formed a stable bond with PCAPAN (Fig. 3E). In contrast, APAN could only form weak dynamic binding with LPS. Further analysis of the energies between different components (Fig. 3F and G) revealed that the binding of APAN to LPS primarily stemmed from hydrophobic interactions. However, after grafting PC groups, the electrostatic binding between PCAPAN to LPS became stronger. Consequently, the total binding force exerted on LPS was significantly enhanced.

The 50-ns simulation pose of the PCAPAN–LPS system revealed that LPS did not integrate into the PCAPAN polymer chain but remained tightly affixed to it (Fig. S6), which may be related to the steric hindrance of the O-antigen portion [52]. Notably, the negative charge center of the core oligosaccharide aligned with the choline positive charge center of PCAPAN, resulting in a dipole–dipole attraction. This observation corroborated the molecular docking findings, indicating a significant electrostatic interaction between the PC group and the core oligosaccharide of LPS. Consequently, we hypothesize that during the adsorption process of LPS by PCAPAN-Fe, the PC group engaged with the core oligosaccharide of LPS via electrostatic forces, thereby facilitating the adhesion of LPS to the PCAPAN-Fe surface.

### Effects of MNPs on blood composition and endothelial cells

Following theoretical investigations into the adsorption mechanisms between 4 MNPs and LPS, we have now shifted focus to systematically evaluate the application potential of these MNPs in sepsis hemopurification therapies. The adsorption dynamics in blood environments fundamentally differ from those in simple ionic solutions, as evidenced by the marked differences in LPS adsorption capacities observed between DPBS buffer and whole blood systems. The primary challenge in hematological adsorption lay in interference from blood cells and plasma proteins. To address this issue, we first conducted comprehensive studies of the interactions between each MNP and blood cellular components, because these interactions govern both the efficacy and the safety of MNPs in blood applications.

**Fig. 4.**
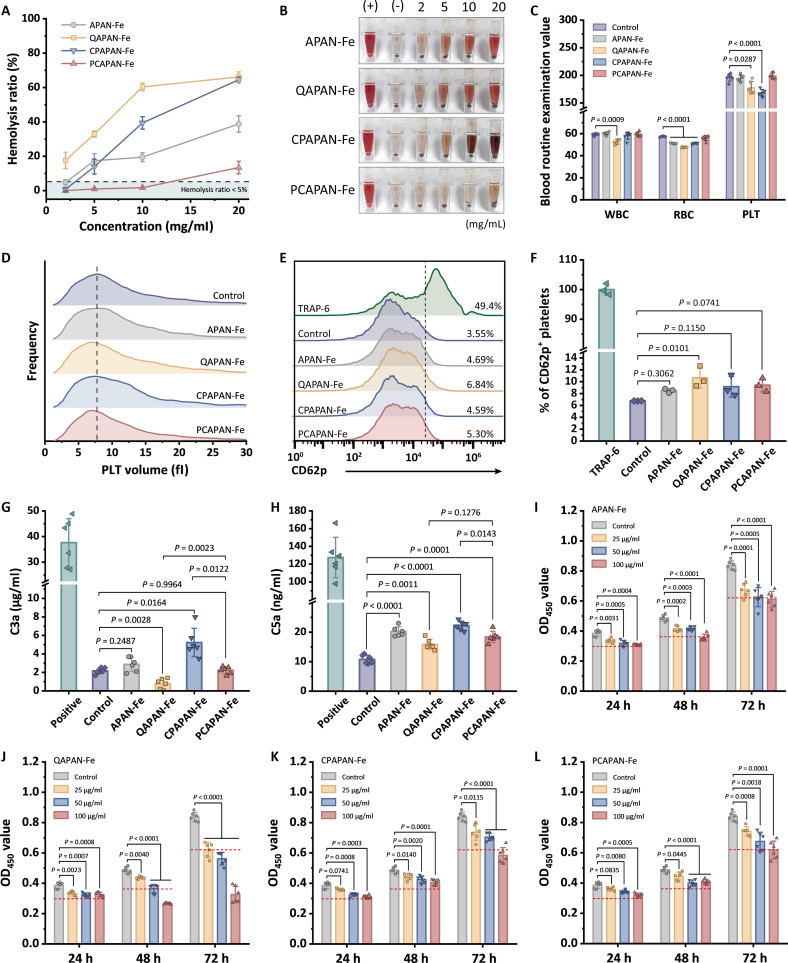
Effects of MNPs on blood cells, complement system, and endothelial cells. (A) Hemolysis ratio of the blood after incubation with MNPs. (B) Optical images of the supernatants after centrifugation of the RBC suspensions. (C) Blood cell counts for pristine blood and MNP-treated blood (WBC, 10^8^/l; RBC, 10^11^/l; PLT, 10^9^/l). (D) Volume distribution of pristine PLTs and MNP-treated PLTs. (E) Histogram of fluorescence intensity of PLT CD62p expression after incubation with MNPs. (F) Relative amount of CD62p expression in PLT co-incubated with MNPs. (G) Generation of C3a and (H) C5a in the blood after incubation with MNPs. (I) Cell proliferation measurements of EA.hy926 cultured with APAN-Fe, (J) QAPAN-Fe, (K) CPAPAN-Fe, and (L) PCAPAN-Fe on 24, 48, and 72 h. Red dashed line represents 75% cell viability.

The hemolysis ratio of gradient concentrations of MNPs was examined to gauge the extent of the damage to blood cells. At a concentration of 2 mg/ml, QAPAN-Fe led to a hemolysis ratio of >10% due to the strong interaction between the quaternary ammonium and the red blood cell (RBC) membrane, destructing RBCs (Fig. 4A and B). Interestingly, CPAPAN-Fe caused a dramatic increase in hemolysis at high concentrations, presumably because the CP group had a complementary structure to the PC headgroup on the RBC membrane, causing the RBC adhesion and aggregation, which led to their destruction. Additionally, the acidic phosphate group exposed by the CP denatured hemoglobin. In contrast, PCAPAN-Fe had a lesser effect on RBCs. The PC group formed a hydrated layer and repelled RBCs due to its charge orientation, which matched the PC headgroup on the RBC membrane, thus reducing RBC destruction. Therefore, even at concentrations as high as 10 mg/ml, the hemolysis ratio of PCAPAN-Fe remained less than 5%.

Subsequent evaluation of MNP effects on hematological parameters revealed distinct biocompatibility profiles. QAPAN-Fe and CPAPAN-Fe induced nonspecific adhesion to circulating blood cells (Fig. 4C), significantly reducing platelet counts (PLTs) with concurrent microaggregate formation (Fig. 4D). These MNPs also adhered to leukocytes [white blood cells (WBCs)], inducing significant alterations in cellular volume and morphological complexity (Fig. S7). In contrast, PCAPAN-Fe maintained baseline blood cell counts without inducing platelet aggregation or WBC morphological changes.

Nanoparticles have the potential to cause PLT aggregation and the formation of vascular thrombosis by activating GPIIb/IIIa [53]. Thus, it is essential to assess the effect of MNPs on PLTs. CD62p serves as a significant marker of PLT aggregation, and its expression was examined by flow cytometry in platelet-rich plasma (PRP) after exposure to MNPs. Thrombin receptor activating peptide-6 (TRAP-6) served as a positive control that significantly increased CD62p expression and activated GPIIb/IIIa, and the normal PRP served as a negative control. PLT CD62p activations by the MNPs were nonsignificant, except for QAPAN-Fe (Fig. 4E and Fig. S8). The activation of PLTs by QAPAN-Fe was attributed to charge stimulation by cations [54] (Fig. 4F). However, this effect was mitigated by the charge-balancing properties of zwitterions [55].

Next, we assessed the activation level of the complement system by MNPs. Complement activation would generate free complement anaphylatoxins C3a and C5a, which mediated the activation of immune cells through a cascade reaction. Compared to normal blood, PCAPAN-Fe did not result in elevated levels of C3a and showed a mild increase in C5a (Fig. 4G and H). This observation may be related to PCAPAN-Fe size-mediated adsorption of IgM and conformational changes of its surface charge affecting complement proteins [56].

The inevitable interaction between MNPs and vascular endothelial cells upon systemic administration raises critical biocompatibility concerns. Cellular damage caused by such interactions may trigger the release of damage-associated molecular patterns (DAMPs), thereby exacerbate inflammatory responses and elevate thrombotic risks, both factors known to compromise prognostic outcomes in sepsis management. The cytotoxicity of MNPs was evaluated using the human umbilical vein cell line (EA.hy926). QAPAN-Fe exhibited more significant cytotoxicity, with the number of cells decreasing dramatically compared to the control, as the QAPAN-Fe concentration increased and the coculture time was prolonged (Fig. 4I and J). In contrast, the cytotoxicity of the zwitterion-modified MNPs were significantly reduced (Fig. 4K and L), with PCAPAN-Fe maintaining >75% cell viability when cocultured with cells at a concentration of 100 μg/ml for 48 h. However, PCAPAN-Fe also manifested worrisome cytotoxicity when high concentrations of the MNP were cocultured with the cells for 72 h. Consistent results were reflected in microscopic photographs of live/dead fluorescent staining (Figs. S9 to S11). These suggested that PCAPAN-Fe did not exhibit toxicity during short extracorporeal contact with blood. Nevertheless, significant cytotoxicity occurred when a high concentration was infused back into the body, leading to prolonged contact with endothelial cells.

### The MNP–protein interactions and LPS selectivity

Beyond cellular interactions, a critical challenge in blood-based LPS adsorption is plasma protein interference. Competitive adsorption and biofouling by plasma proteins increase mass transfer resistance, reducing adsorbent efficacy and posing risks of protein depletion and thrombogenic complications. We hypothesize that zwitterionic materials with precise charge strength and orientation could enhance LPS selectivity by stabilizing hydration layers and screening charges to minimize protein interactions.

**Fig. 5.**
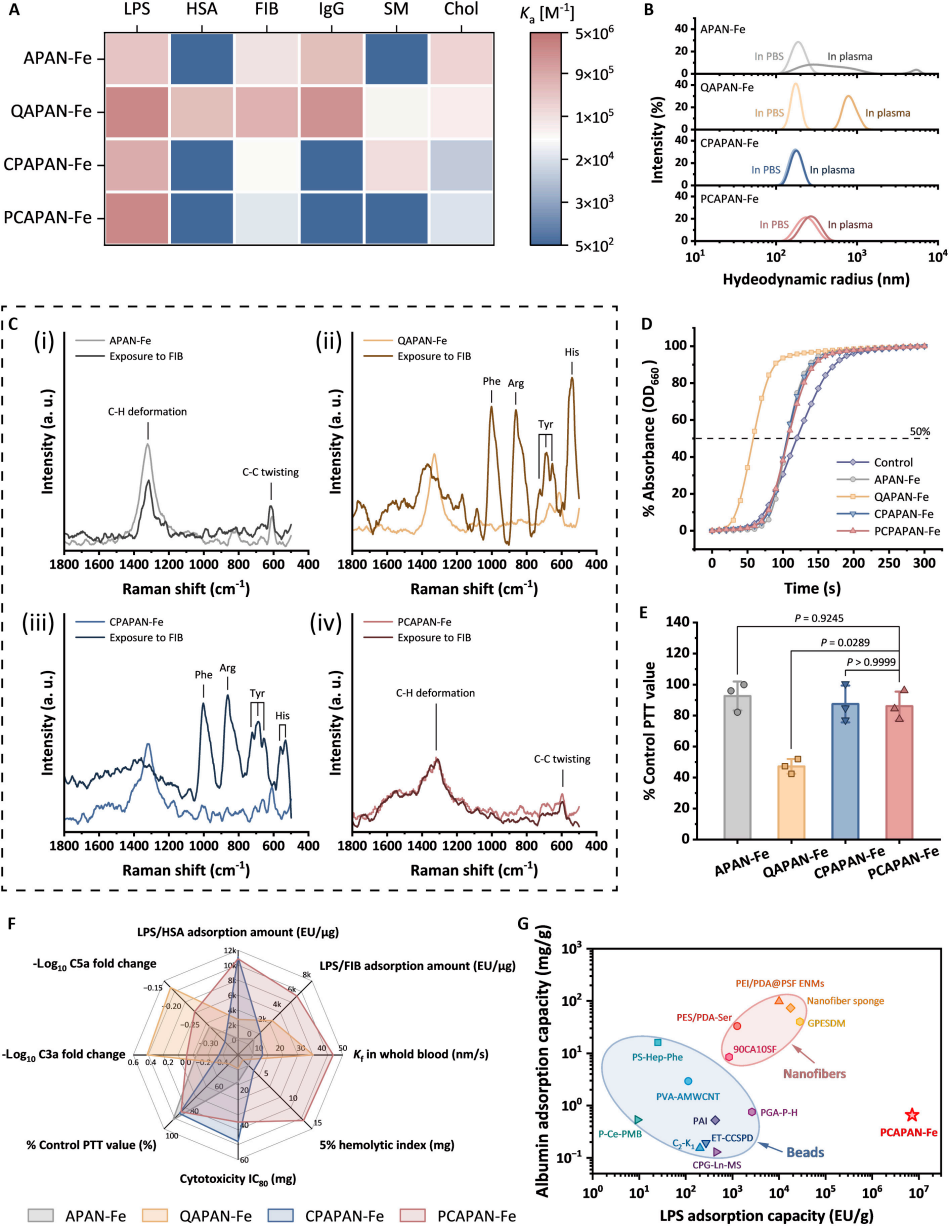
Comprehensive assessment of the MNP interactions with plasma proteins for effectiveness and safety evaluation. (A) Heatmap of association constants of MNPs with major biomolecules in blood. (B) Hydrodynamic radius of MNPs in PBS buffer and plasma. (C) Raman spectra of the MNPs [(i) APAN-Fe, (ii) QAPAN-Fe, (iii) CPAPAN-Fe, (iv) PCAPAN-Fe] before and after co-incubation with FIB. (D) PTT coagulation curves of PPP following co-incubation with MNPs. (E) Normalized PTT values. (F) Comprehensive quantitative radar chart of the effectiveness and safety of the 4 MNPs. (G) Ashby plot of LPS and protein adsorption capacities for reported adsorbents and PCAPAN-Fe.

Based on this hypothesis, we measured the association constants (*K*_a_) of MNPs with key plasma components, containing human serum albumin (HSA), FIB, immunoglobulin G (IgG), as well as sphingomyelin (SM; a major cell membrane component) and cholesterol (Chol; a common hydrophobic plasma molecule) using ITC, with LPS as a reference (Fig. 5A; raw data were displayed in Fig. S12). APAN-Fe and QAPAN-Fe exhibited high affinity for plasma proteins (HSA and FIB) and Chol, indicating nonspecific adsorption that interferes with LPS binding. In contrast, the zwitterionic CPAPAN-Fe and PCAPAN-Fe minimized protein interactions. PCAPAN-Fe uniquely avoided SM binding due to structural mimicry of cell membranes via its PC headgroup, preventing blood cell adhesion, while CPAPAN-Fe showed complementary SM affinity. Single-protein adsorption assays (Fig. S13) confirmed PCAPAN-Fe’s anti-fouling properties, with minimal HSA/FIB adsorption. Hydrodynamic radius measurements (Fig. 5B) further revealed PCAPAN-Fe’s plasma stability versus aggregation-prone APAN-Fe and QAPAN-Fe.

We observed that PCAPAN-Fe retained a measurable binding affinity for FIB, albeit with a low *K*_a_ value, which remains a concern. Hard binding of FIB to the MNP surfaces can induce conformational changes in FIB, potentially triggering particle aggregation and thrombotic risks [57]. To evaluate this, confocal laser Raman spectroscopy was employed to analyze the surface composition of MNPs after co-incubation with FIB and subsequent buffer washing. Raman spectroscopic analysis revealed no FIB-specific signals on PCAPAN-Fe surfaces after washing (Fig. 5C), indicating reversible soft interactions. In contrast, QAPAN-Fe and CPAPAN-Fe retained FIB-specific peaks corresponding to amino acid residues, confirming irreversible adsorption.

To further assess thrombogenic risks, we measured partial thromboplastin time (PTT) after recalcification of MNP-treated platelet-poor plasma (PPP). Coagulation curves (Fig. 5D) revealed a significant procoagulant tendency for QAPAN-Fe, while PCAPAN-Fe and CPAPAN-Fe showed negligible effects. Quantitatively, QAPAN-Fe reduced PTT by approximately 50% compared to pristine PPP (Fig. 5E). Notably, zwitterionic modifications (CP or PC) maintained baseline PTT levels, demonstrating their hemocompatibility advantages.

A comprehensive radar analysis (Fig. 5F) highlighted PCAPAN-Fe’s optimal balance: high LPS selectivity, minimal protein/cell interactions, and hemocompatibility. Benchmarking against existing materials (Fig. 5G and Table S6) revealed that traditional polymer beads suffer from low LPS capacity, while nanofiber membranes trade high LPS adsorption for excessive protein fouling. PCAPAN-Fe resolves this conflict through reasonable zwitterionic design and nanoscale effects, achieving unmatched LPS capacity with negligible protein loss, surpassing current clinical standards.

The inherent conflict between LPS adsorption and protein selectivity has long posed a critical challenge in developing sepsis hemopurification materials. PCAPAN-Fe resolved this dilemma by combining rational charge compatibility design with nanoscale effects, achieving both ultrahigh LPS adsorption capacity and exceptionally low protein adsorption. This breakthrough performance surpassed existing materials, addressing the longstanding trade-off between specificity and efficiency in clinical blood purification applications.

### Design of the modular extracorporeal magnetic retrieval system

**Fig. 6.**
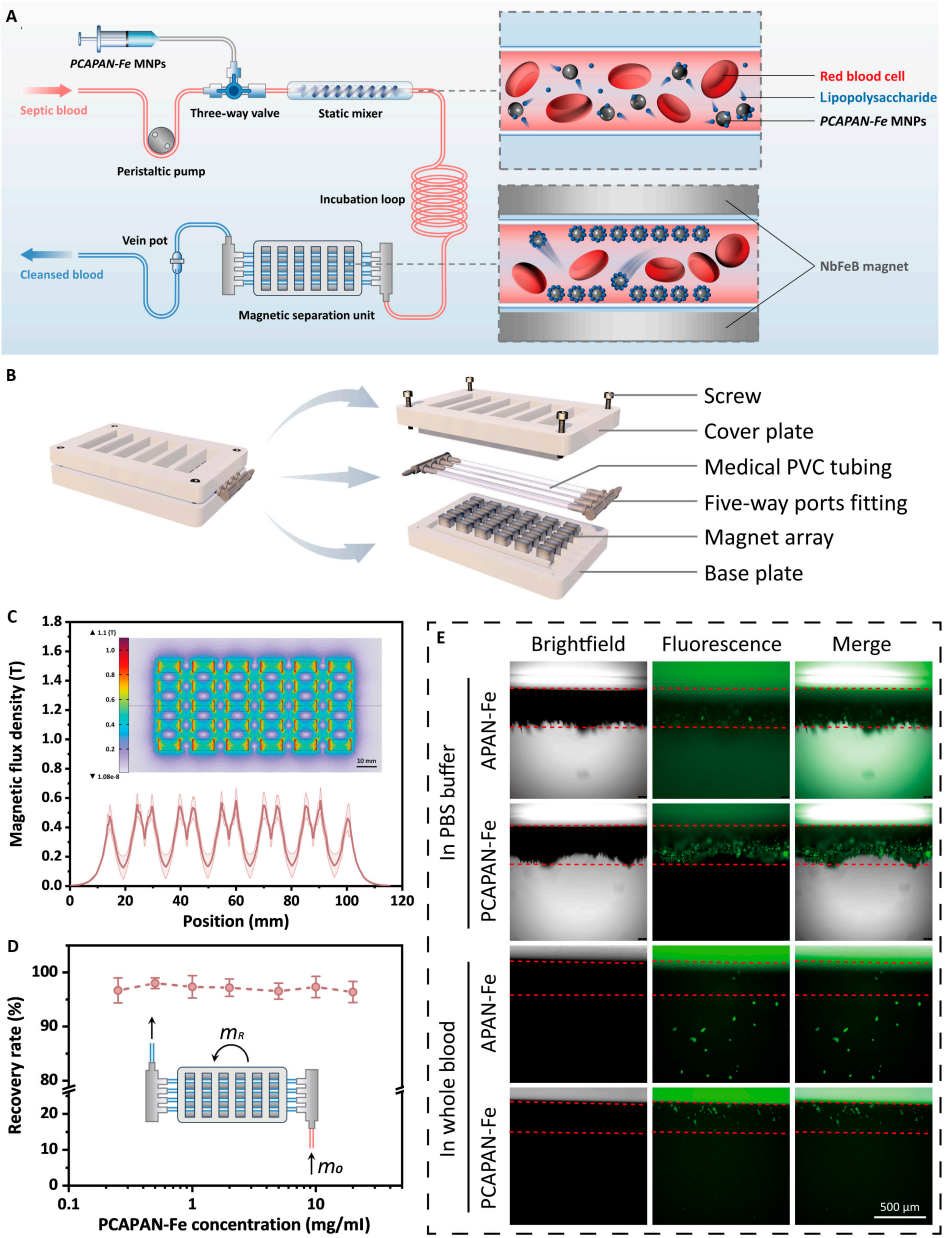
Design of ELMAS and evaluation of its magnetic separation performance. (A) Schematic diagram of the ELMAS treating strategy. (B) Part assembly design drawing of the magnetic separation unit. (C) Values of magnetic flux density along the direction of fluid motion in the magnetic separation unit. Insert: Magnetic flux density distribution pattern of the magnetic array. (D) Magnetic recovery rates of PCAPAN-Fe with different concentrations. (E) Brightfield and fluorescence images of PCAPAN-Fe captured by FITC–LPS and subsequently attracted to the tube wall by a magnet in the ELMAS tubing. The red dashed lines indicate the regions of PCAPAN-Fe magnetically separated, with the magnet positioned directly above these areas.

Although PCAPAN-Fe achieved effective LPS clearance, minimizing plasma protein interactions while maintaining high LPS affinity, their clinical translation is hindered by systemic nanoparticle exposure risks, including cumulative and metabolic toxicity. To resolve this, we developed the extracorporeal LPS-targeting magnetic array system (ELMAS), which confined PCAPAN-Fe to an external circuit for localized LPS adsorption without systemic nanoparticle entry (Fig. 6A).

Distinct from reported integrated microfluidic magnetic separation systems, the ELMAS device employs a detachable modular configuration. Its core architecture features a peristaltic pump-driven extracorporeal circuit where blood interfaces with PCAPAN-Fe suspensions through an in-line static mixer to optimize contact dynamics (Fig. S14). The subsequent separation phase utilizes bifurcated tubing integrated with externally mounted magnetic array modules (Fig. S15). When engaged with the magnetic array, this branching conduit enables efficient nanoparticle capture through localized high-gradient field confinement. Medical-grade polyvinyl chloride (PVC) tubing with standardized connectors ensures modular assembly, featuring single-use sterilizable disposable pathways while maintaining reusable external magnetic components. Crucially, the system permits comprehensive degassing via standardized extracorporeal priming protocols prior to magnetic module assembly, thereby eliminating air embolism risks inherent in monolithic microfluidic designs.

Critical to ELMAS efficacy is the magnetic separation unit’s optimized design. A NdFeB magnet array with alternating polarities (Fig. 6B) generates a high-gradient magnetic field (simulated flux density in Fig. 6C), significantly outperforming conventional bar magnets (Fig. S16). When PCAPAN-Fe passed through the magnetic separation unit, the particle generated 3 velocity components, which are flow velocity (*v*_flow_), magnetic drag velocity (*v*_mag_), and gravitational settling velocity (*v*_g_) (Fig. S17). Due to the extremely small mass of nanoscale PCAPAN-Fe, *v*_g_ was considered negligible in the calculations. *v*_flow_ was calculated from the blood flow rate, and the residence time of PCAPAN-Fe was determined in the magnetic field region. *v*_mag_ was related to the magnetic flux density that determined the time PCAPAN-Fe took to be transported to the tubing wall. The vibrating sample magnetometer (VSM) was utilized to measure the hysteresis curve of PCAPAN-Fe (Fig. S18), allowing for the determination of its saturation magnetization, which is necessary for calculating *v*_mag_. Based on *v*_flow_ and *v*_mag_, we designed the dimension of the magnetic separation unit (Fig. S19). The magnetic separation unit achieved over 96% recovery for any concentration in a single run, proving its effectiveness in capturing PCAPAN-Fe (Fig. 6D).

Functional validation using fluorescein isothiocyanate (FITC)-labeled LPS confirmed PCAPAN-Fe’s ability to adsorb and magnetically separate LPS in both PBS and whole blood (Fig. 6E). Under magnetic guidance, LPS-bound PCAPAN-Fe migrated to tubing walls, achieving complete phase separation without nanoparticle reinfusion. This confirms ELMAS’s capacity for extracorporeal LPS clearance while eliminating systemic toxicity risks.

### Evaluation of ELMAS loaded with the PCAPAN-Fe therapeutic effects in sepsis biological models

To demonstrate the feasibility of applying ELMAS with PCAPAN-Fe in practical sepsis therapy, an assessment of the in vivo LPS clearance performance is essential. LPS could stimulate blood monocytes/macrophages polarizing to the M1 phenotype and releasing proinflammatory cytokines. Before conducting animal experiments, the mouse macrophages (RAW264.7) were used as a model to ascertain whether PCAPAN-Fe could effectively capture LPS, and thereby protecting the macrophage from stimulation. In this experiment, an LPS solution was added to RAW264.7, followed by the addition of PCAPAN-Fe. In the control group, APAN-Fe was added. After 1 h of co-incubation, MNPs were magnetically removed, and the cells were further incubated for 12 h. Cellular expression of inducible nitric oxide synthase (iNOS) and the typical proinflammatory cytokine tumor necrosis factor-α (TNF-α) was analyzed semiquantitatively by immunofluorescence staining. Compared to the untreated and APAN-Fe-treated groups, the fluorescence signals of iNOS and TNF-α were significantly attenuated after the PCAPAN-Fe treatment (Fig. S20). Consistent conclusions were drawn from statistical analysis of fluorescence intensities (Fig. S21).

Then, enzyme-linked immunosorbent assay (ELISA) was used to quantify the cytokine concentration released by RAW264.7 with and without the PCAPAN-Fe treatment. Preliminary cell counting kit-8 (CCK-8) testing confirmed the nontoxicity of the experimental concentration on cells (Fig. S22). The levels of released TNF-α and interleukin-6 (IL-6) were then measured (Fig. S23). The PCAPAN-Fe treatment significantly reduced cytokine release from RAW264.7. The above results indicated that PCAPAN-Fe could effectively bind LPS and inhibit the subsequent inflammatory response.

**Fig. 7.**
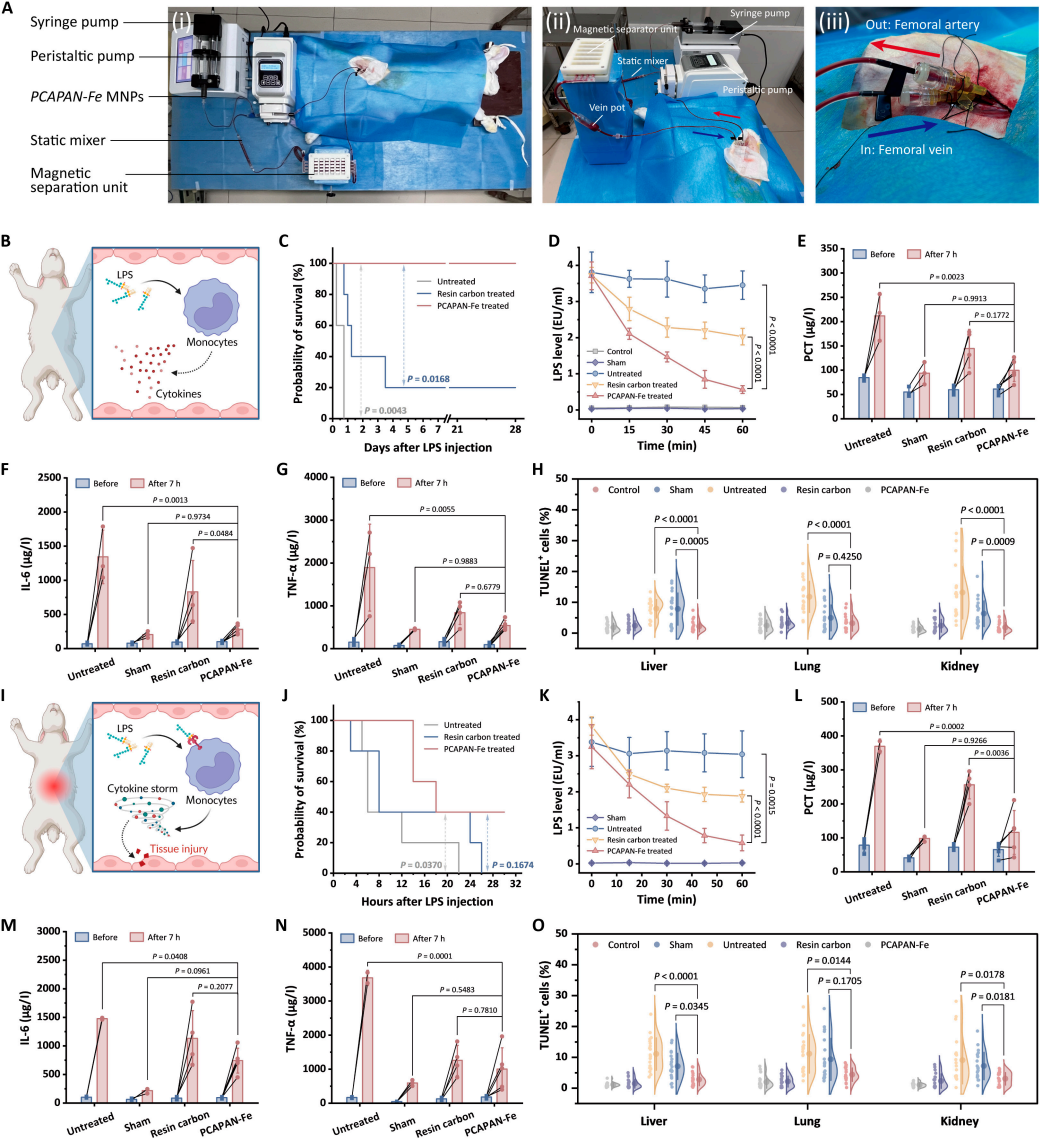
Therapeutic efficacy of PCAPAN-Fe-loaded ELMAS in rabbit models of sepsis. (A) Photograph of ELMAS for treating septic rabbits. (i) Overall view, (ii) side view, and (iii) cannulation site of femoral artery and vein. (B) Schematic illustration of immune status in early-stage septic rabbit models. (C) Survival rate curves following early intervention in septic rabbit models (*n* = 5). (D) Temporal variations in blood LPS levels during early-stage sepsis intervention. (E) Post-intervention PCT, (F) IL-6, and (G) TNF-α level variations in early-stage septic rabbits. (H) Statistical analysis of TUNEL-positive rates in early-stage septic rabbits (*n* = 3 technical replicates from 5 biological replicates for each group). (I) Schematic representation of immune dynamics in progressive-stage septic rabbits. (J) Survival rate curves after therapeutic intervention in progressive-stage sepsis models (*n* = 5). (K) Blood LPS level kinetics during progressive-stage sepsis treatment. (L) Post-treatment PCT, (M) IL-6, and (N) TNF-α profiles in progressive-stage septic rabbits. (O) Quantification of TUNEL-positive cells in progressive-stage septic rabbits after therapy (*n* = 3 technical replicates from 5 biological replicates for each group).

Following cellular-level validation, we established an ELMAS-based extracorporeal circulation therapeutic protocol in rabbits (Fig. 7A). Specifically, the femoral artery and vein of rabbits were surgically isolated to establish an extracorporeal circulation circuit. In this system, the PCAPAN-Fe suspensions were continuously infused into the extracorporeal circuit, where they mixed with whole blood before being sequentially passed through a mixer, extension tubing, and magnetic separation unit prior to venous reinfusion. The circulatory pathway was configured such that blood was withdrawn from the femoral artery and subsequently returned via the femoral vein after undergoing the full adsorption–separation sequence (Movie S1).

In the early stages of sepsis, massive bacterial death leads to substantial LPS accumulation in the bloodstream [58]. The LPS overload activates immune cells, thereby inducing a systemic inflammatory response (Fig. 7B). Thus, early hemopurification targeting LPS removal is pivotal for improving survival in sepsis. To assess the therapeutic efficacy of the PCAPAN-Fe-loaded ELMAS devices during early-stage sepsis, we employed a validated rabbit model (*n* = 5). Sepsis was induced via intravenous LPS injection (0.5 mg/kg), followed by immediate 1-h ELMAS extracorporeal therapy (Fig. S24). Control groups included untreated sepsis, commercial resin carbon adsorbent therapy, and sham-operated animals (saline injection + ELMAS). The PCAPAN-Fe treatment achieved 100% 28-d survival (Fig. 7C and Movie S2), contrasting with untreated (0%) and resin carbon groups (20%). This survival benefit correlated with rapid 84.7% LPS clearance (versus 45.6% for commercial resin carbon), reducing circulating LPS to subpathogenic thresholds within 1 h (Fig. 7D and Fig. S25). Hematological normalization occurred by 24 h post-treatment, with leukocyte and PLTs matching sham levels (Figs. S26 and S27).

To investigate PCAPAN-Fe’s therapeutic effect on LPS-mediated inflammatory responses, we measured procalcitonin (PCT), IL-6, TNF-α, and C-reactive protein (CRP). These 4 biomarkers provide critical prognostic insights into the early progression of infection (Fig. S28). PCT, a biomarker for acute infection, surges during early-stage systemic inflammation, with its concentration correlating positively with infection severity. Plasma PCT levels in untreated rabbits spiked markedly at 7 h post-LPS injection (Fig. 7E), indicating systemic inflammatory response activation. Conversely, the PCAPAN-Fe-treated rabbits exhibited significantly lower PCT levels compared to untreated and resin carbon groups, suggesting mitigated inflammation.

The explosive release of proinflammatory cytokines such as IL-6 and TNF-α is closely linked to sepsis-related mortality [59]. Plasma IL-6 and TNF-α levels in the PCAPAN-Fe group were substantially reduced compared to untreated rabbits, with outcomes superior to the resin carbon group (Fig. 7F and G). Excessive IL-6 drives the acute-phase response to infection and further stimulates hepatic CRP synthesis, which peaks approximately 24 h post-infection [60]. CRP levels measured at 1-d post-treatment (Fig. S29) revealed severe secondary inflammation in the resin carbon group, whereas the PCAPAN-Fe group showed no significant CRP increase, confirming effective LPS eradication and prevention of downstream inflammatory cascades. Untreated rabbits (all deceased within 1 d) yielded no CRP data.

LPS drives multiorgan injury through dual mechanisms: direct cytotoxicity and cytokine storm-induced systemic pathophysiological dysregulation, both exacerbating clinical management challenges and mortality. To quantify PCAPAN-Fe-mediated organ protection via rapid LPS clearance, we analyzed hepatic/renal biomarkers in treated rabbits. The resin carbon-treated rabbits exhibited pathological alkaline phosphatase (ALP) suppression, alongside elevated alanine transaminase (ALT) and aspartate transaminase (AST), confirming hepatic inflammation (Fig. S30). In contrast, PCAPAN-Fe maintained physiological ALP, ALT, and AST levels matching sham controls. Parallel renal assessments revealed resin carbon-induced creatinine and urea elevation (Fig. S31), contrasted by PCAPAN-Fe’s preservation of glomerular filtration.

Histopathological analysis was performed to evaluate the inhibitory effects of different early intervention strategies on sepsis-induced multiorgan damage (Fig. S32). Hematoxylin and eosin (H&E)-stained sections revealed that untreated septic controls exhibited glomerular capillary congestion (renal), alveolar collapse (pulmonary), and hepatocellular necrosis with neutrophilic infiltration (hepatic). Semiquantitative lesion area scoring (Fig. S33) demonstrated that PCAPAN-Fe achieved near-physiological damage scores (0 to 0.86) across major organs, while resin carbon failed to effectively suppress tissue damage (2.53 to 3.00) compared to untreated controls (2.27 to 2.40). Terminal deoxynucleotidyl transferase-mediated deoxyuridine triphosphate nick end labeling (TUNEL) quantification confirmed that PCAPAN-Fe inhibited apoptosis in visceral tissues by clearing LPS. Compared to untreated and resin carbon-treated groups, PCAPAN-Fe treatment significantly reduced DNA fragmentation-positive cells in hepatic, pulmonary, and renal cells of rabbits (Fig. 7H).

A critical clinical consideration is that most sepsis cases require intervention during the progressive phase, where established cytokine storms and elevated LPS levels exacerbate inflammation, culminating in multiorgan damage (Fig. 7I) [61,62]. To evaluate PCAPAN-Fe’s therapeutic potential in advanced sepsis, we established a progressive-stage rabbit model with delayed ELMAS treatment initiation until clinical symptom manifestation (tachypnea, hyperthermia, and leukopenia; Fig. S34). Given the model’s irreversible pathophysiological impacts, an ethical protocol set the observational endpoint at 30 h post-operation.

PCAPAN-Fe achieved a 40% 24-h survival ratio (Fig. 7J), representing a significant improvement over untreated (0% survival). The adsorbent demonstrated robust performance with 82.2% LPS clearance (Fig. 7K and Fig. S35) and restored WBC and PLT counts to sham levels within 24 h (Figs. S36 and S37), confirming rapid LPS neutralization and systemic recovery. Inflammatory markers were analyzed to assess systemic responses (Fig. S38). The PCAPAN-Fe treatment reduced PCT (Fig. 7L), IL-6 (Fig. 7M), and TNF-α (Fig. 7N) levels compared to untreated controls, signifying controlled immune activation. Furthermore, no significant elevation in CRP was observed in the PCAPAN-Fe group at 24 h post-infection (Fig. S39), confirming suppression of acute inflammatory cascades through effective LPS removal.

In progressive-stage sepsis, particular attention must be given to cytokine storm-induced secondary organ injury. Resin carbon-treated animals exhibited pathological ALP suppression, indicative of LPS-induced neuraminidase-mediated dephosphorylation impairment [63], alongside significant ALT and AST elevation (Fig. S40). In contrast, PCAPAN-Fe demonstrated markedly lower ALT and AST elevation compared to resin carbon therapy, suggesting improved clinical outcomes. Notably, the PCAPAN-Fe treatment caused no significant ALP reduction versus resin carbon intervention. This differential response indicates that PCAPAN-Fe preserves LPS-mediated dephosphorylation defense mechanisms through efficient LPS clearance during sepsis progression. Meanwhile, renal functional biomarkers (Fig. S41) revealed stable creatinine and urea nitrogen levels in PCAPAN-Fe-treated subjects, contrasting with progressive elevation in the resin carbon group, confirming effective mitigation of sepsis-induced nephropathy.

Histopathological evaluation via H&E staining and TUNEL fluorescence (Fig. S42) revealed distinct organ injury patterns: Untreated and Resin carbon-treated groups exhibited hepatic vacuolar degeneration, glomerular capillary congestion, and neutrophilic alveolar wall infiltration. The PCAPAN-Fe treatment reduced histopathological severity while preserving hepatocellular architecture, alveolar septal integrity, and glomerular vascular patency. Semiquantitative injury scoring (Fig. S43) further demonstrated PCAPAN-Fe’s capacity to limit damage progression (final scores: 0.72 to 1.80 versus 2.16 to 2.88 in untreated). TUNEL-positive cell rates were significantly lower in the PCAPAN-Fe-treated subjects compared to untreated groups (Fig. 7O), confirming apoptosis suppression. By clearing LPS, PCAPAN-Fe extended the therapeutic window via delayed irreversible organ failure thresholds.

In order to assess the safety and reliability of the ELMAS strategy, as well as to test whether PCAPAN-Fe entered the body’s circulation causing a worrisome cumulative toxicity, we tested the iron content in the heart, liver, spleen, lungs, and kidneys of all the PCAPAN-Fe-treated rabbits. The results revealed no notable changes in the iron content of the major organs of rabbits after treatment (Fig. S44). Furthermore, we employed the fluorescence-labeled PCAPAN-Fe to investigate the accumulation of nano-adsorbents in various organs following the ELMAS treatment (Fig. S45). PCAPAN-Fe administered without the magnetic separation unit was used as a positive control. Significant accumulation was observed in the spleen and lungs, with additional nano-adsorbent adherence to the vascular walls in the heart, liver, and kidneys. In contrast, administration with the magnetic separation device resulted in a marked decrease in nanoparticle accumulation across all examined organs. Quantification via fluorescent labeling revealed an approximately 90% reduction in nanoparticle uptake by the spleen and lungs (Fig. S46). This demonstrated that ELMAS could effectively intercept PCAPAN-Fe without inducing in vivo toxicity.

Following the validation of PCAPAN-Fe’s effectiveness in adsorbing LPS during sepsis and the safety of the ELMAS device in 2 animal models, we considered the translational potential of this therapeutic strategy for clinical applications. In the rabbit model, the total LPS load introduced into the bloodstream was approximately 540 EU (3 EU/ml × 180 ml of blood volume). In contrast, septic patients typically have a blood LPS load exceeding 1 EU/ml [11], corresponding to a total endotoxin load of approximately 4,000 to 5,000 EU. Given the larger blood volume and higher tolerable blood flow rates in humans compared to rabbits, and based on the total endotoxin load and animal experimental results, the clinical application could consider increasing the PCAPAN-Fe concentration or the infusion rate of the suspension, provided the instantaneous blood nanoparticle concentration does not exceed 10 mg/ml.

Regarding the refinement of the magnetic separation unit, the ELMAS system designed for rabbits operated at a blood flow rate of 7 ml/min and achieved complete recovery of MNPs, resulting in a calculated *v*_mag_ of PCAPAN-Fe within the ELMAS device of approximately 4.2 mm/min. For human applications, which require higher blood flow rates (200 to 300 ml/min, representative of human clinical settings), sufficient residence time for the complete recovery of PCAPAN-Fe can be achieved by increasing the number of channels in the magnetic separation module, reducing the diameter of these tubes, or appropriately extending their length.

In clinical management of severe sepsis, blood purification is rarely employed as a standalone therapy but rather integrated with supportive measures such as oxygen supplementation and lactate clearance for systemic stabilization [64]. PCAPAN-Fe enhanced this multimodal approach by rapidly clearing LPS and blocking subsequent inflammatory cascades and secondary tissue damage, thereby extending the critical therapeutic window and improving patient survival outcomes.

Preclinical validation across 2 animal models demonstrates PCAPAN-Fe’s dual-phase efficacy: (a) In early-stage sepsis intervention, PCAPAN-Fe achieved rapid LPS adsorption without safety concerns, effectively preventing cytokine storm initiation and significantly improving survival rates. (b) In progressive-stage sepsis management, PCAPAN-Fe mitigated secondary immune dysregulation in cases of established organ injury, reducing pathological progression and extending the actionable therapeutic window. This strategy bridges the critical gap between pathogen clearance and immunomodulation, providing a versatile solution for sepsis treatment across disease progression stages.

## Conclusion

This study addressed 2 fundamental barriers impeding nano-adsorbent clinical translation in septic blood purification: (a) compromised functionality and biocompatibility due to charge-mediated nonspecific protein adsorption and (b) challenges in nano-adsorbent retrieval from blood and compatibility of separation devices with blood purification systems. We pioneered zwitterion-functionalized MNPs (PCAPAN-Fe) engineering with precision charge orientation. The PC ligands undergo differential dipole realignment upon contact with proteins versus LPS, simultaneously achieving maximal antifouling efficacy and selective LPS capture. PCAPAN-Fe demonstrates exceptional adsorption kinetics and record capacity in both buffer and whole blood environments.

Through a modular detachable design strategy for extracorporeal magnetic separation devices, we developed the ELMAS device. This system effectively addresses key challenges of conventional magnetic separators: manufacturing complexity, sterilization difficulties, and air removal challenges, thereby providing a translatable solution for nano-adsorbent-based blood purification applications. In a rabbit sepsis model, ELMAS loaded with PCAPAN-Fe achieved 100% survival rate following early intervention, accompanied by significant reductions in proinflammatory cytokines and prevention of cytokine storm cascades. Histopathological and biochemical analyses confirmed marked attenuation of multiorgan damage and apoptosis. Even in progressive sepsis, PCAPAN-Fe maintained an 82.2% LPS clearance efficiency, extending the 24-h survival rate to 40%, highlighting its broad therapeutic applicability.

However, this study has limitations. The investigation into the interactions between ligands with different charge structures and LPS was conducted in aqueous or buffer environments, which may not fully reflect their interactions within the complex blood environment. Undoubtedly, studying intermolecular interactions in such a complex blood system poses significant challenges. This issue has rarely been addressed in previous studies. Future research should focus on exploring the mechanisms within this complex blood system. Additionally, this study solely concentrated on LPS clearance in sepsis treatment. Although removing LPS from septic blood is critical for treatment, it is not the only consideration. The excessive inflammation triggered by LPS may cause irreversible damage, necessitating a multidimensional treatment approach (including fluid resuscitation, immunomodulation, and lung-protective ventilation). Due to the lack of a comprehensive treatment strategy, fatal events continued to occur in progressive-stage septic rabbit models, despite a significant reduction in LPS concentration in blood. Further research is also needed to discuss how the ELMAS device can be systematically scaled up and applied in clinical treatment.

In summary, this work resolves the performance–biosafety paradox in LPS-targeting nano-adsorbent design, delivering a clinically translatable sepsis nanotherapeutic platform. Future efforts will focus on scalable nanomanufacturing and integration into multimodal critical care protocols. Our findings establish a paradigm for developing selective, biocompatible nano-adsorbents, advancing the frontier of blood purification technologies.

## Materials and Methods

### Materials

PAN (average *M*_w_ = 149,000 to 151,000), DETA (99%), allyl trimethylammonium chloride (ATEAC; 98%), tetrahydrofuran (THF; ≥99.9%, anhydrous), acetonitrile (MeCN; 99.8%, anhydrous), trimethylamine (TMA) solution (2 M in THF, anhydrous), triethylamine [TEA; gas chromatography (GC), >99.5%], methanol (99.8%, anhydrous, H_2_O ≤ 100 ppm), pyridine (99.8%, anhydrous), iron (III) chloride hexahydrate [FeCl_3_; analytical reagent (AR), 99%], sodium hydroxide (NaOH; electronic grade, 99.9%), and bovine serum albumin (BSA; New Zealand Precision Grade, ≥98.0%) were purchased from Aladdin Reagent (China). 2-Chloro-1,3,2-dioxaphospholane-2-oxide (COP), *N*,*N*-dimethylallylamine (DMAA), iron (II) chloride tetrahydrate (FeCl_2_; ReagentPlus, 98%), ammonium cerium (IV) nitrate [(NH_4_)_2_Ce(NO_3_)_6_; ACS, ≥98.5%], LPSs (from *E. coli* O55:B5, purified by phenol extraction), and FITC-conjugated LPS (from *E. coli* O55:B5) were purchased from Sigma-Aldrich (USA). Ethanol (C_2_H_5_OH; AR) and hydrochloric acid (HCl; AR) were purchased from Chron Chemicals (China). Normal saline, PBS (pH 7.4), and DPBS (containing Ca^2+^ and Mg^2+^) were purchased from Thermo Fisher Scientific (USA). The resin carbon used as the commercial control was provided by Chongqing Healcom Co. Ltd. (RC Series). A deionized lab water system was used to prepare ultrapure (UP) water in all experiments.

### Synthesis of MNPs

#### Synthesis of APAN-Fe

PAN (2 g) was dispersed in 60 ml of UP water; the suspension was vacuum degassed and nitrogen bubbled. The PAN suspension was heated to 90 °C, and 20 ml of FeCl_2_/FeCl_3_ mixture (0.2 M of FeCl_2_ and 0.3 M of FeCl_3_ in UP water) was added slowly and dropwise. Subsequently, 20 ml of DETA was added dropwise with vigorous stirring. The reaction temperature was reduced to 70 °C to prevent intense reaction that may result in PAN crosslinking by DETA, and the reaction was maintained for 24 h. After the reaction, APAN-Fe was collected by magnetic separation, ultrasonically washed 5 times with ethanol and water, and then lyophilized for storage.

#### Synthesis of QAPAN-Fe

The prepared APAN-Fe was dried for >10 h to ensure adequate drying. APAN-Fe (2 g) was suspended in 80 ml of UP water, deoxygenated by bubbling nitrogen gas, and heated to 60 °C. ATEAC (3.5 g) was dissolved in 20 ml of water, deoxygenated by bubbling nitrogen gas, and then added to the APAN-Fe suspension. Subsequently, 0.5 g of (NH_4_)_2_Ce(NO_3_)_6_ was added to the reaction system, which was then allowed to react overnight at 60 °C [65]. After the reaction, QAPAN-Fe was collected by magnetic separation, ultrasonically washed 5 times with ethanol and water, and then lyophilized for storage.

#### Synthesis of CPAPAN-Fe

First, we synthesized double-bond functionalized CP ligand molecules. The prepared APAN-Fe was dried for >10 h to ensure adequate drying. APAN-Fe (2 g) was suspended in 80 ml of UP water, deoxygenated by bubbling nitrogen gas, and heated to 60 °C. Prop-2-enyl choline phosphate (p-eCP, 3.5 g) was dissolved in 20 ml of water, deoxygenated by bubbling nitrogen gas, and then added to the APAN-Fe suspension. Subsequently, 0.5 g of (NH_4_)_2_Ce(NO_3_)_6_ was added to the reaction system, which was then allowed to react overnight at 60 °C. After the reaction, CPAPAN-Fe was collected by magnetic separation, ultrasonically washed 5 times with ethanol and water, and then lyophilized for storage.

#### Synthesis of PCAPAN-Fe

The prepared APAN-Fe was dried for >10 h to ensure adequate drying. APAN-Fe (2 g) was dispersed in 20 ml of anhydrous THF. The suspension was cooled to −25 °C, and 100 μl of TEA was added. COP (2.14 g) was first dissolved in 5 ml of anhydrous THF and then added dropwise to the suspension within 5 min. After the reaction at −25 °C for 5 h, the suspension was centrifuged at 3,283*g* for 15 min at 4 °C abandonment solution, and the solids were resuspended with 20 ml of anhydrous MeCN. Next, 2.5 ml of TMA was added to the suspension. The reaction was carried out at 70 °C for 24 h (Fig. S1E). After the reaction, PCAPAN-Fe was collected by magnetic separation, ultrasonically washed 5 times with ethanol and water, and then lyophilized for storage.

### In vitro LPS removal experiments

MNPs were dispersed in pyrogen-free DPBS buffer (100 mg/ml) and sonicated for 15 min to disperse. LPS was dissolved in DPBS buffer, and its initial concentration was determined by *Limulus* amebocyte lysate (LAL) assay. The MNP suspension was added to the LPS solution for a final 1 mg/ml concentration. The mixed solution was incubated with shaking at 37 °C. The mixed solution was centrifuged at 3,283*g* at different times, and the supernatant was taken to test the LPS concentration. Pyrogen-free tubes and pipettes (BIOENDO, China) were used in all test sessions. The same method assessed the LPS clearance performance of MNPs in human blood. In addition, by varying the initial concentration of the LPS solution, the theoretical maximum LPS adsorption capacities of MNPs were determined. Detailed experimental protocols can be found in the Supplementary Materials.

### Isothermal titration calorimetry

ITC experiments were conducted on the Nano ITC system (TA Instrument, USA) at 37 °C. The analytes were dissolved in UP water and loaded into a syringe. The concentrations used for the different analytes were as follows: LPS (0.107 mM), HSA (0.100 mM), FIB (0.147 mM), IgG (0.084 mM), SM (1.00 mM), and Chol (5.17 mM). MNPs dispersed (1 mg/ml) in PBS buffer were loaded into the sample cell, and the reference cell was filled with PBS. LPS solution (2.5 μl) was injected into the cell each time, accompanied by stirring at 350 rpm, for a total of 20 injections. The injection interval was 360 s to ensure that the thermal signal could return to the baseline before the next injection. The exothermic heat values were analyzed using NanoAnalyze software (TA Instrument, USA). The molar amount of MNP was calculated according to the molecular weight of PAN, and an “Independent” model was used to fit the data. The dissociation constant (*K*_d_), association constant (*K*_a_), enthalpy change (Δ*H*), entropy change (Δ*S*), and other parameters were thus obtained.

### Computer simulation

The binding modes between the core oligosaccharide and various ligands were determined using global flexible docking with AutoDock 4.2 software. The MD of the interaction between PCAPAN and LPS was simulated using GROMACS (version 2020.6). The molecular structure of LPS was constructed based on a previously reported series [51]. Further details on the computer simulations are described in the Supplementary Materials.

### Hemocompatibility evaluations

To assess the potential impact of MNPs on blood cells, a hemolysis test was conducted. The hemolysis rate was calculated based on the absorbance of free hemoglobin. Additionally, blood cell counts were determined in MNP-treated blood using a hematology analyzer (BC-5100, Mindray, China). Clotting times and coagulation factor activity were measured using an automated coagulometer (CA-2500, Sysmex, Japan). Flow cytometry was carried out with an anti-CD41/anti-CD62p double staining protocol to assess the activation levels of the platelets induced by MNPs. The activation of complement by MNPs was evaluated via ELISA. Additionally, a clinical chemistry analyzer (Cobas 6000, Roche, Switzerland) was utilized to assess changes in protein concentration in the serum after co-incubation with MNPs. The detailed experimental procedures are described in the Supplementary Materials.

### Design of the ELMAS device

PVC tubing with an internal diameter of 1.25 mm was used as the main extracorporeal tubing. A peristaltic pump tubing (internal diameter: 3.2 mm; long: 120 mm) was connected at the arterial end, followed by a 3-way valve (WEGO Medical, China) connected to the MNP injection channel. A static mixer (diameter: 6 mm, long: 74 mm) was attached to the line to enhance the radial mixing of MNPs and blood. In the magnetic separation unit, the internal diameter of the tubing was 3.5 mm. A vein pot (WEGO Medical, China) was attached to remove air bubbles after the magnetic separation unit. The extracorporeal tubing was sterilized using ethylene oxide before application to animal experiments.

The cover and base plate of the magnetic separation unit were produced by 3-dimensional printing. An array of NdFeB magnets (5 × 5 × 10 mm, N52, TAIXIONG, China) with poles arranged in reverse parallel were secured to the base plate by instant adhesive (Ergo 5400, Switzerland). The magnetic flux density was simulated using COMSOL Multiphysics (version 6.1). The *v*_mag_ of PCAPAN-Fe was calculated according to Eq. (1) [66].

$$v_{\mathrm{mag}}\left(x\right)=\frac{2R^{2}\Delta \chi \;B\left(x\right)}{9\mu_{0}\eta }$$ (1)

where *R* is the radius of PCAPAN-Fe (m), *Δχ* is the magnetic susceptibility difference between PCAPAN-Fe and blood (dimensionless), and *B*(*x*) is the magnetic flux density gradient (T^2^/m). *μ*_0_ is the vacuum permeability (N/A^2^), and *η* is the dynamic viscosity of blood (Pa·s).

The dimensions of the magnetic separation unit were determined based on the minimum effective magnetic recovery distance (*D*_m_) [26], which is defined as the distance component along the tubing axial direction during the magnetic movement of PCAPAN-Fe. *D*_m_ must be smaller than the radius of the magnetic separation unit tubing to effectively capture PCAPAN-Fe. *D*_m_ was calculated according to Eqs. (2) and (3).

$$D_{m}=v_{\mathrm{mag}}\left(x\right)\times t_{\mathrm{res}}$$ (2)

$$t_{\mathrm{res}}=\frac{\mathrm{n\pi}d^{2}L}{4Q}$$ (3)

where *t*_res_ is the residence time of PCAPAN-Fe in the tubing while flowing through the device (s), and *n* is the number of branches in the tubing at the magnetic separation unit. *d* is the internal diameter of the tubing in the magnetic separation unit (cm), *L* is the length of the tubing in the magnetic separation unit (cm), and *Q* is the flow rate of the blood (ml/s).

Based on the calculations, 30 magnets were used to arrange in a 5 × 6 array. The range of the magnetic recovery unit was designed to be 86 mm. The PCAPAN-Fe suspension (20 ml) with concentrations ranging from 0.25 to 20 mg/ml was single passed through the magnetic separation unit (flow rate = 7 ml/min). PCAPAN-Fe in the line was flushed out after removing the magnets, which was used to evaluate the recovery rate [Eq. (4)].

$$\text{Recovery rate}\;\left(\%\right)=\frac{m_{R}}{m_{0}}\times 100\%$$ (4)

where *m*_0_ is the initial mass of PCAPAN-Fe in the suspension, and *m*_R_ is the mass of retained PCAPAN-Fe.

### Therapeutic efficacy evaluations of ELMAS loaded with PCAPAN-Fe in a septic rabbit model

#### Establishment of early-stage sepsis rabbit model

First, the rabbits were weighed, followed by intravenous injection of 0.5 mg/kg LPS via the marginal ear vein. One minute later, blood was drawn from the central ear artery to measure LPS levels. Modeling was considered successful if the blood LPS concentration exceeded 3 EU/ml. Treatment was immediately initiated using ELMAS loaded with PCAPAN-Fe (each group *n* = 5).

#### Establishment of progressive-stage sepsis rabbit model

First, the rabbits were weighed, followed by intravenous injection of 0.5 mg/kg LPS via the marginal ear vein. The rabbits were continuously monitored for approximately 60 min post-injection until they exhibited characteristic symptoms, including tachypnea (rapid breathing), elevated body temperature (rectal temperature > 40 °C), and a dramatic drop in WBC count. Blood was then collected from the central ear artery to measure LPS levels. Successful modeling was confirmed when the blood LPS concentration exceeded 3 EU/ml. Subsequently, treatment was administered using ELMAS loaded with PCAPAN-Fe (each group *n* = 5).

#### ELMAS loaded with the PCAPAN-Fe therapy

After collecting preoperative blood, the rabbits’ right femoral artery and vein were isolated and punctured with an indwelling needle (24-gauge, HONGKANG, China) for connection to the extracorporeal circulatory line. The line was precharged with heparin sodium solution (100 U/ml) at a flow rate of 20 ml/min for 30 min. Air bubbles in the lines were eliminated by tapping the tubing. Heparin sodium was used as an anticoagulant with a loading dose of 500 U/kg.

Based on the extracorporeal blood flow rate used in human hemoperfusion and adjusted proportionally according to the difference in body weight between humans and rabbits, we determined an appropriate flow rate while avoiding excessively high speeds that could lead to adverse events such as heart failure. Accordingly, the extracorporeal blood flow rate was set at 7 ml/min. The PCAPAN-Fe saline suspension (1 mg/ml) was continuously infused into the extracorporeal circulation (ECC) circuit via a 3-way connector using an infusion pump at a rate of 0.12 ml·kg^−1^·min^−1^ for a duration of 1 h. Heparin sodium-anticoagulated (1 ml) and EDTA-anticoagulated blood (1 ml) were taken from the femoral artery side at 15-min intervals during the ECC to determine LPS levels and blood routine tests, respectively. At the treatment’s end, saline returned the tubing blood to the rabbit. The surgical incision was carefully sutured and thoroughly sterilized with iodophor. Upon reaching the observational endpoint, the rabbits were euthanized via anesthetic overdose, and major organs were harvested and sectioned for histopathological analysis.

#### PCT and cytokine concentration tests in blood

Anticoagulant-free whole blood was taken from the rabbits in each experimental groups before treatment and 7 h after LPS injection, respectively. The collected whole blood underwent clotting for over 1 h at room temperature, followed by plasma separation through centrifugation at 644*g* for 15 min. The concentrations of PCT, IL-6, and TNF-α in the serum were assessed using commercially available ELISA kits (CUSABIO, China), with 3 parallel controls established for each serum sample.

#### Blood biochemical test

Anticoagulant-free whole blood was taken from rabbits in each experimental groups before treatment, 7 h after LPS injection and 24 h after LPS injection, respectively. The collected whole blood underwent clotting for over 1 h at room temperature, followed by plasma separation through centrifugation at 644*g* for 15 min. The collected plasma was tested for the levels of ALP, AST, ALT, direct bilirubin (BILD), total bilirubin (BILT), creatinine (CREA), urea nitrogen (UREA), and uric acid (UA) using a clinical chemistry analyzer (Cobas 6000, Roche, Switzerland).

#### Pathological section study

The liver, lung, and kidney tissues of the rabbits were carefully extracted and promptly fixed in a 4% paraformaldehyde solution. Following a 10-d fixation period, the paraffin sections were stained with H&E and 4′,6-diamidino-2-phenylindole (DAPI)/TUNEL (DeadEnd Fluorometric TUNEL System, Promega, USA). The assessment of tissue damages was conducted through the imaging of pathology sections utilizing a digital slide scanner (SLIDEVIEW VS200, Olympus, Japan).

Semiquantitative histopathological scoring was performed on tissue sections using H&E staining to assess morphological evidence of cellular injury, including nuclear fragmentation, cytoplasmic vacuolization, and inflammatory infiltration: no damage = 0, abnormal field (0, 25%] = 1, (25%, 50%] = 2, (50%, 75%] = 3, (75%, 100%] = 4. Scoring was performed using a blind analysis.

The cell injury positivity rate was quantified by analyzing TUNEL and DAPI colocalization. TUNEL signals (green fluorescence, indicative of apoptotic nuclei) and DAPI counterstains (blue fluorescence, marking total nuclei) were imaged using fluorescence microscopy. ImageJ software was employed for threshold-based segmentation of TUNEL-positive areas and particle analysis of total DAPI-stained nuclei.

### Statistical analysis

Each experiment was conducted and quantified at least in triplicate. The results were presented as the mean ± SD for each sample, with error bars depicted on all graphs. Significance differences between the 2 samples were calculated using the 2-tailed Student’s *t* test. For comparisons among 3 or more experimental groups, a one-way analysis of variance (ANOVA) test followed by Tukey’s post hoc comparison test was employed. In the animal experiment, survival analysis was performed using the log-rank test. All statistical analyses were performed using GraphPad Prism software (version 10.4.2). A *P* value less than 0.05 was considered statistically significant at a 95% confidence level.

## Ethical Approval

All biological experiments were approved and conducted by West China Hospital of Sichuan University, and all experimental protocols complied with relevant laws and national regulations (GB/T 16886.4-2003/ISO 10993-4:2002). The experiments involving human blood were approved by West China Hospital of Sichuan University (ethics approval number: No.2024553, Chictr.org.cn registration number: ChiCTR2400083626). The experiments involving animals in this study complied with the guidelines for ethical review of animal welfare (GB/T 35892-2018) and were approved by the animal management and ethics committee, West China Hospital of Sichuan University (ethics approval number: No.20230608002). During the experimental period, all animals had sufficient living space and free access to water and food. The experimental animals were all sacrificed using intravenous overdose of sodium pentobarbital.

## Data Availability

All data needed to evaluate the conclusions in the paper are present in the paper or the Supplementary Materials.
