# Supplementary material for: Charge-Engineered LPS-Targeting Magnetic Nano-adsorbents with Optimized Harvesting Strategy Advance Sepsis Blood Purification Nanotherapeutic
Source: Research (Wash D C). 2025 Dec 8;8:0991. doi: 10.34133/research.0991 (PMC12682954; doi:10.34133/research.0991)
Supplement: Supplementary 1 — Supplementary Methods Figs. S1 to S46 Tables S1 to S6 Movies S1 and S2 [file research.0991.f1.zip › Supplemental information.docx]

**Supplemental information**

**Charge-engineered LPS-targeting magnetic nano-adsorbents with optimized harvesting strategy advance sepsis blood purification nanotherapeutic**

Xianda Liu^1^, Shengjun Cheng^1^, Xijing Yang^2^, Yilin Wang^1^, Shifan Chen^1^, Ziyue Ling^1^, Yujie Xiao^1^, Weifeng Zhao^1,*^, and Changsheng Zhao^1,3^

^1^ College of Polymer Science and Engineering, State Key Laboratory of Advanced Polymer Materials, Sichuan University, China

^2^ The Experimental Animal Center of West China Hospital, Sichuan University, China

^3^ Med-X Center for Materials, Sichuan University, China

*Corresponding author. E-mail: weifeng@scu.edu.cn

**Content**

[**S1. Supplemental methods** 1](#_Toc205507501)

[**S1.1 Characterization** 1](#_Toc205507502)

[**S1.2 In-vitro LPS removal experiments** 1](#_Toc205507503)

[*S1.2.1 LPS concentration testing method* 1](#_Toc205507504)

[*S1.2.2 LPS clearance experiment in DPBS buffer* 2](#_Toc205507505)

[*S1.2.3 LPS clearance experiment in whole blood* 2](#_Toc205507506)

[*S1.2.4 Adsorption isotherm fitting* 2](#_Toc205507507)

[*S1.2.5 Adsorption kinetic models fitting* 3](#_Toc205507508)

[**S1.3 Computer simulation** 5](#_Toc205507509)

[*S1.3.1 Electrostatic Potential (ESP) Charge Distribution Simulation* 5](#_Toc205507510)

[*S1.3.2 Molecule docking* 5](#_Toc205507511)

[*S1.3.3 Molecular dynamics (MD) simulation* 5](#_Toc205507512)

[**S1.4 Hemocompatibility evaluations** 6](#_Toc205507513)

[*S1.4.1 Hemolysis* 6](#_Toc205507514)

[*S1.4.2 Complete blood count (CBC) in vitro* 6](#_Toc205507515)

[*S1.4.3 PLT activation analysis* 7](#_Toc205507516)

[*S1.4.4 Complement activation analysis* 7](#_Toc205507517)

[**S1.5 Cytotoxicity test** 8](#_Toc205507518)

[**S1.6 Affinity assessment of the MNPs for major blood biomolecules** 8](#_Toc205507519)

[*S1.6.1 Raman spectroscopy analysis of the MNPs after co-Incubation with FIB solution* 8](#_Toc205507520)

[*S1.6.2 Quantification of Protein Adsorption* 9](#_Toc205507521)

[**S1.7 Partial thromboplastin time (PTT) measurement** 9](#_Toc205507522)

[**S1.8 Inflammatory signaling detection of macrophage** 9](#_Toc205507523)

[**S1.9 Nanoparticle *in-vivo* residue analysis** 10](#_Toc205507524)

[**S2. Supplemental figures** 12](#_Toc205507525)

[**S3. Supplemental tables** 57](#_Toc205507526)

[**Reference** 63](#_Toc205507527)

# **S1. Supplemental methods**

## **S1.1 Characterization**

The microscopic morphology of the nanoparticles was observed by scanning electron microscopy (SEM, Apreo 2C, Thermo Fischer Scientific, USA) and transmission electron microscopy (TEM, Talos F200x, Thermo Fischer Scientific, USA). The elemental composition of the MNPs were investigated by X-ray photoelectron spectroscopy (XPS, K-Alpha, Thermo Fischer Scientific, USA). Fourier transform infrared spectroscopy (FTIR, Nicolet 560, USA) was used to study the chemical structure of the NMPs. Dynamic light scattering (DLS, Zetasizer Nano-ZS, Malvern, UK) was used to test the zeta potential and hydrodynamic radius of the MNPs. In the zeta potential test, the system pH was adjusted by hydrochloric acid and sodium hydroxide, and the experiments were carried out at 37 °C.

## **S1.2 In-vitro LPS removal experiments**

### *S1.2.1 LPS concentration testing method*

The LPS concentration in the PBS buffer was assessed using the endotoxin endpoint chromogenic kit (BIOENDO, China). The test samples were diluted with pyrogen-free water (BIOENDO, China) and maintained at a constant temperature of 37 °C. Following this, the diluted samples were combined with Limulus amebocyte lysate (LAL) regent and incubated at 37 °C for a designated period. Afterward, the chromogenic substrate was introduced, and the mixture was further incubated at 37 °C for a designated period before the stop solution was added. The absorbance at 405 nm was immediately measured. A standard curve was generated using LPS standards, and the LPS concentration in the solution was determined through regression fitting.

The LPS concentration in blood was assessed using the Endosafe^®^ Portable Test System (PTS™, Charles River Laboratories, USA) [1]. Plasma was separated by centrifuging whole blood samples at 2576×g for 15 min. The obtained plasma was then diluted using pyrogen-free water. Subsequently, 25 μL of the diluted plasma was extracted and applied to the LAL cartridge (Charles River Laboratories, USA), and the LPS concentration in the plasma was determined through the PTS™ preset program.

### *S1.2.2 LPS clearance experiment in DPBS buffer*

LPS was introduced into a DPBS buffer, and the concentration was maintained at approximately 100 EU/mL (~10 ng/mL in the DPBS). Subsequently, 10 mg of the MNPs were added to 10 mL of the LPS solution and incubated at 37 °C with shaking. After 15, 30, 60, 90, 120, 150 and 180 min of co-incubation, 5 μL of the solution was extracted and subjected to a 100-fold dilution with pyrogen-free water, followed by centrifugation at 3283×g for 3 min, and the supernatant was extracted to test the endotoxin concentration. The removal rate was calculated using **Equation S1**.

| $\text{Removal rate}\text{ (\%) =}\frac{\text{C}_{\text{0}}\text{-}\text{C}_{\text{t}}}{\text{C}_{\text{0}}}\text{×100\%}$ | (S1) |
| --- | --- |

where *C*_0_ is the initial LPS concentration (EU/mL), and *C*_t_ is the LPS concentration after co-incubation at different times (EU/mL).

### *S1.2.3 LPS clearance experiment in whole blood*

LPS was introduced into fresh whole blood anticoagulated with recombinant hirudin (Boatman Biotech, China) to create a septic blood model, with the concentration of approximately 20 EU/mL (~10 ng/mL in the blood). 2 mL of the septic blood were treated with 2 mg of the MNPs and incubated at 37 °C with shaking. After 15, 30, 60, 90, 120, 150 and 180 min of co-incubation, 50 μL of blood was centrifuged at 3283×g for 3 min, and the plasma was collected. The supernatant was diluted 20-fold with pyrogen-free water for testing the LPS concentration. The removal rate was calculated using **Equation S1**.

### *S1.2.4 Adsorption isotherm fitting*

A series of gradient concentrations of LPS solutions (10~8,000 EU/mL) were prepared using pyrogen-free water. 2 mL of the LPS solution was combined with 2 mg of the MNPs, tightly sealed, and incubated at 37 °C for 24 h. Afterward, the supernatant was collected by centrifugation at 3283×g for 3 min, and the LPS concentration of the solution after co-incubated was tested. The equilibrium adsorption capacities (*q*_e_) at various initial LPS concentrations were calculated using **Equation S2** and fitted to the Langmuir adsorption isotherm model (**Equation S3**) [2], Temkin adsorption isotherm model (**Equation S4**) [3], and Freundlich adsorption isotherm model (**Equation S5**) [4].

| $\text{q}_{\text{e}}\text{ = }\frac{\left( \text{C}_{\text{0}}\text{-}\text{C}_{\text{t}} \right)\text{×}\text{V}}{\text{m}}$ | (S2) |
| --- | --- |

where *q*_e_ (EU/mg)is the equilibrium adsorption capacity, *V* (mL) is the volume of LPS solution, *m* (mg) is the mess of the NMPs,

| $\text{q}_{\text{e}}\text{ = }\frac{\text{q}_{\text{max }}\text{K}_{\text{L}}\text{C}_{\text{e}}}{\text{1 + }\text{K}_{\text{L}}\text{C}_{\text{e}}}$ | (S3) |
| --- | --- |

where *q*_max_ (EU/mg) is the theoretical maximum adsorption capacity, *C*_e_ (EU/mL) is the equilibrium concentration of the LPS solution, *K*_L_ is the constants related to adsorption affinity.

| $\text{q}_{\text{e}}\text{ = }\frac{\text{RT}}{\text{b}_{\text{T}}}\text{ln}\text{(}\text{K}_{\text{T}}\text{C}_{\text{e}}\text{)}$ | (S4) |
| --- | --- |

where *b*_T_ (J/mol) and *K*_T_ are the constants.

| $\text{q}_{\text{e}}\text{ = }\text{K}_{\text{F}}{\text{C}_{\text{e}}}^{\text{1}\text{/}\text{n}}$ | (S5) |
| --- | --- |

where n characterizes the nonlinear adsorption behavior and surface heterogeneity*.* and *K*_F_ is the constants related to adsorption affinity.

### *S1.2.5 Adsorption kinetic models fitting*

Changes in the concentration of LPS in the DPBS or whole blood co-incubated with MNPs were monitored over a 180 min period, following the experimental methodology described in **S1.2.2** and **S1.2.3**, respectively. The collected data were fitted to pseudo-first-order model (**Equation S6**), pseudo-second-order adsorption kinetic model (**Equation S7**) [5], intraparticle diffusion model (**Equation S8**) [6], and film diffusion model (**Equation S9**) [7].

| $\text{q}_{\text{t}}\text{ = }\text{q}_{\text{e}}\text{(}\text{1-}\text{e}^{\text{-}\text{k}_{\text{1}}\text{t}}\text{)}$ | (S6) |
| --- | --- |

where *q*_t_ (EU/mg) is the amount of LPS absorbed by the MNPs at time *t*, *k*_1_ (min^-1^) is the rate constants of pseudo-first-order model.

| $\text{q}_{\text{t}}\text{ = }\frac{\text{k}_{\text{2}}{\text{q}_{\text{e}}}^{\text{2}}\text{t}}{\text{1}\text{+}\text{k}_{\text{2}}\text{q}_{\text{e}}\text{t}}$ | (S7) |
| --- | --- |

where *k*_2_ (mg·EU^-1^·min^-1^) is the rate constants of pseudo-second-order model.

| $\text{q}_{\text{t}}\text{ = }\text{k}_{\text{W-M}}\text{ t}^{\text{ 1/2}}$ | (S8) |
| --- | --- |

where *k*_W-M_ (EU·mg^-1^·min^-1/2^) is the rate constants of Weber-Morris Intraparticle Diffusion Model.

| $\text{A t}\text{ =}\text{ -}\text{ (1-}\frac{\text{q}_{\text{e}}}{\text{q}_{\text{m}}}\text{)}\text{ }\text{ln}\text{ (1-}\frac{\text{q}_{\text{t}}}{\text{q}_{\text{e}}}\text{) - (}\frac{\text{V}\text{C}_{\text{0}}}{\text{m}\text{q}_{\text{e}}}\text{-1) ln (1-}\frac{\text{m}\text{q}_{\text{e}}}{\text{V}\text{C}_{\text{0}}}\text{ }\frac{\text{q}_{\text{t}}}{\text{q}_{\text{m}}}\text{)}$ | (S9) |
| --- | --- |
| $\text{A}\text{ }\text{=}\text{ }\frac{\text{3}\text{m}\text{k}_{\text{f}}}{\text{rVρ}_{\text{p}}}\text{ (}\frac{\text{V}\text{C}_{\text{0}}}{\text{m}\text{q}_{\text{e}}}\text{ - }\frac{\text{q}_{\text{e}}}{\text{q}_{\text{m}}}\text{) }$ | (S10) |

Where *A* is a parameter defining by **Eq. S10**, *q*_m_ (EU/mg) is the theoretical maximum adsorption capacity derived from the Langmuir adsorption isotherm. *C*_0_ (EU/mL) is the initial LPS concentration, *m* (mg) is the adsorbent mass, *V* (mL) is the LPS solution volume, *r* (nm) is the adsorbent radius, *ρ*_p_ (mg/cm^3^) the adsorbent particle density, and *k*_f_ (nm/s) is the external film mass transfer coefficient [7].

## **S1.3 Computer simulation**

### *S1.3.1 Electrostatic Potential (ESP) Charge Distribution Simulation*

The simulation of ESP distribution diagrams was conducted using Materials Studio 2020 software (BIOVIA, USA). Initially, molecular structures of the APAN, the QAPAN, the CPAPAN, and the PCAPAN were imported and optimized via energy minimization until convergence. Subsequently, the total electron density of the molecules was simulated using the DMol3 module. The electrostatic potential was then predicted and mapped onto the total electron density surface to generate the ESP distribution diagrams.

### *S1.3.2 Molecule docking*

The polymer side chains of the MNP were globally flexibly docked with the core oligosaccharide and lipid A portions of LPS. Using AutoDock 4.2 software, the active region search space covered the entire receptor molecule. The Lamarckian genetic algorithm was used to generate the conformations of both receptor and ligand molecules, and AutoDock was used for docking scoring. The scenario with the lowest binding free energy was selected for result presentation. The results were visualized using Discovery Studio 2019 (BIOVIA, USA).

### *S1.3.3 Molecular dynamics (MD) simulation*

Atomistic MD simulations have been performed in the GROMACS (version 2020.6) simulation package, using the General Amber force field (GAFF2) and the SPCE water model. 6 polymers with 100 repeat units were first inserted into a cubic box around 8 nm using the amorphous builder in the Materials Studio 2020 software (BIOVIA, USA). The polymer structure was equilibrated for 20 ns before expansion along the z-axis for further interaction with the LPS molecules. 5 LPS molecules were inserted into the polymer box. More than 15,000 water molecules were added to solvate the whole system. Ions were added by randomly replacing water molecules to neutralize the system's charge. After thousands of steps of energy minimization, the systems were equilibrated under the NPT ensemble and followed the production runs of 50 ns. The temperature was coupled to 298 K using the Nose-Hoover method, and the pressure was coupled to 1 atm using the Parrinello-Rahman method. The cutoff scheme of 1.2 nm was implemented for the non-bonded interactions, and the Particle Mesh Ewald method with a Fourier spacing of 0.1 nm was applied for the long-range electrostatic interactions. All covalent bonds with hydrogen atoms were constrained using the LINCS algorithm.

## **S1.4 Hemocompatibility evaluations**

### *S1.4.1 Hemolysis*

Whole blood anticoagulated with sodium citrate was centrifuged at 644×g for 10 min to isolate RBCs. The RBCs were diluted with 2-fold saline and separated by centrifugation to purify. The washing procedure was repeated for 5 times, and the RBCs were diluted with saline to 5×10^5^ cells/mL. The MNPs (1 mg/mL) were incubated with 1 mL of the RBC suspension, and saline and water were used as negative and positive controls, respectively. After 3 h of incubation at 37 °C, the suspension was centrifuged at 3283×g for 15 min, and the supernatant was collected to determine the absorbance at 540 nm by a microplate spectrophotometer (Multiskan SkyHigh, Thermo Fischer Scientific, USA). The hemolysis ratio was determined using the **Equation S11**.

| $\text{Hemolysis ratio}\text{ (\%) = }\frac{\text{A}_{\text{s}}\text{-}\text{A}_{\text{n}}}{\text{A}_{\text{p}}\text{-}\text{A}_{\text{n}}}\text{×100\%}$ | (S11) |
| --- | --- |

where *A*_s_, *A*_p_ and *A*_n_ represent the absorbance values of the MNPs, positive and negative control, respectively.

### *S1.4.2 Complete blood count (CBC) in vitro*

The MNPs were incubated with 500 μL EDTA-anticoagulated whole blood at 37 °C for 2 h. Subsequently, the MNP was separated from the blood using a magnet. The incubated whole blood was thoroughly mixed and subjected to CBC by a hematology analyzer (BC-5100, Mindray, China).

### *S1.4.3 PLT activation analysis*

PLT activation by the MNPs was assessed by flow cytometry. Platelet-rich plasma (PRP) was obtained by centrifugation of sodium citrate-anticoagulated human blood at 800×g for 10 min. 200 μg of the MNPs was mixed with 200 μL of PRP in PP tubes and incubated at 37 °C for 30 min. Untreated and thrombin receptor activator peptide-6 (TRAP-6, Sigma-Aldrich, 0.1 mM) treated PRP were used as negative and positive controls, respectively. After 30 min co-incubation, the MNPs were removed magnetically. 10 μL of PRP was taken and mixed with 90 μL of PPP (PLT was diluted to approximately 10^7^ cells/mL), followed by the addition of 5 μL of anti-Human CD41a-APC (Pharmingen™, USA) and 20 μL of anti-Human CD62p-PE (Pharmingen™, USA), and placed in a 4 °C darkness for 30 min. The samples were halted with 300 µL of flow cytometry staining buffer (eBioscience™, USA) and detected using a flow cytometer (FACSCelesta™, BD, USA) by gating PLT-specific events based on anti-CD41a-APC. The sample signals were normalized based on the average fluorescence intensity of TRAP-6-activated PLTs. All experimental acquisition was performed using FACSDiva™ software. Data analysis used FlowJo™ software (version 10.8.1).

### *S1.4.4 Complement activation analysis*

Collect human whole blood anticoagulated with sodium citrate (1:9 v/v) and add recombinant hirudin to it (16000 ATU/mL, 1:40 v/v). The MNPs (1 mg/mL) were incubated with 150 μL anticoagulated whole blood, and 8 μL divalent cation solution (100 mM, containing Ca^2+^ and Mg^2+^) were added to restore complement activation. The blood containing MNPs were at 37 °C for 30 min. Saline and 25 μg/mL of cobra-venom factor (CVF, Hengfei Biotech., China) were used as negative and positive controls, respectively. After the incubation, the complement activation was stopped by adding 10 μL of EDTA solution (80 mM). The incubated blood was centrifugated at 3283×g for 15 min, and the concentrations of C3a and C5a in the supernatant were measured by Complement C3a Human and Complement C5a Human ELISA Kit (Thermo Fischer Scientific, USA).

## **S1.5 Cytotoxicity test**

EA.hy926 (ATCC: CRL-2922™) cells were grown in Dulbecco’s modified eagle medium (DMEM, Gibco™, USA) supplemented with 10% fetal bovine serum (FBS, Gibco™, USA) and 1% penicillin-streptomycin (5,000 U/mL, Gibco™, USA). The cells were cultured in a humidified atmosphere of 5% CO_2_ at 37 °C. The culture medium was changed each 3 days.

EA.hy926 was inoculated into 48-well plates (15,000 cells/well) and co-cultured with the MNPs at 25, 50, and 100 μg/mL. After 1, 2, and 3 days of incubation, 450 μL of medium and 50 μL of CCK-8 (ZETA™, USA) were added to the cells, and a microplate spectrophotometer read the absorbance at 450 nm after 2 h of incubation. Another co-cultured cell was taken and added with Calcein-AM/Ethidium homodimer I (Invitrogen™, USA), and the cells were observed live and dead by fluorescence microscopy (DMi8, Leica, Germany).

## **S1.6 Affinity assessment of the MNPs for major blood biomolecules**

### *S1.6.1 Raman spectroscopy analysis of the MNPs after co-Incubation with FIB solution*

The MNPs (1 mg/mL) were co-incubated with human FIB (4 mg/mL) for 1 h. The co-incubated MNPs were then separated by centrifugation at 3283×g for 15 min and alternately washed three times with PBS and UP water. Raman spectra of the MNPs before and after FIB co-incubation were acquired using a confocal laser Raman spectrometer (XploRA PLUS, HORIBA, Japan) at 785 nm excitation wavelength, with data collected across the Raman shift range of 500-1800 cm^-1^.

### *S1.6.2 Quantification of Protein Adsorption*

The MNPs (1 mg/mL) were co-incubated with 40 mg/mL HSA or 4 mg/mL FIB for 1 h. The MNPs were alternately washed three times with UP water and PBS buffer to remove unbound proteins. Subsequently, the protein-bound MNPs were incubated with 5 mL of 2% sodium dodecyl sulfate (SDS) solution at 37 °C for 1 h to elute adsorbed proteins. The eluted protein concentration was quantified using the bicinchoninic acid (BCA) colorimetric assay (Pierce™ BCA Protein Assay Kits, Thermo Fisher Scientific, USA) to determine the protein adsorption capacity of the MNPs.

## **S1.7 Partial thromboplastin time (PTT) measurement**

Add 100 μL of 250 mM CaCl₂ solution to a 96-well plate preheated to 37°C. Co-incubate 1 mg/mL MNPs with 200 μL PPP at 37°C for 3 min, then centrifuge to separate the PPP. Transfer 150 μL of the separated PPP to an EP tube. Add 100 μL of phospholipid saline solution (containing 0.2 wt% rabbit brain phospholipids + 0.1 wt% soybean brain phospholipids + 1 wt% BSA) to the EP tube, followed by incubation at 37°C for 3 min. Take 200 μL of the phospholipid-incubated PPP and add it to the pre-CaCl₂-loaded 96-well plate. Use a microplate reader in kinetic mode to measure absorbance at 660 nm, recording data every 10 s for 10 min. The time corresponding to a 50% change in absorbance is defined as the PTT value.

## **S1.8 Inflammatory signaling detection of macrophage**

RAW264.7 (ATCC: TIB-71™) was sellected as the macrophage model. RAW264.7 was inoculated into 48-well plates (150,000 cells/well). LPS (1 μg/mL) was added to the medium, followed by 1 mg/mL of the MNPs. After 1 h of co-incubation, the MNPs were removed by a magnet, and saline was used as a negative control. After the treated cells were incubated for another 6 h, the medium was aspirated, and the cells were fixed with 4% paraformaldehyde for 15 min. After fixation, the cells were permeabilized with 0.25% Triton X-100 solution for 15 min, then rinsed once with PBS. The samples were blocked with 1% BSA solution for 4 h. The anti-iNOS antibody (EPR16635, Abcam, UK) and anti-TNF-*α* antibody (EPR19147, Abcam, UK), were added and incubated at 4 °C for 10 h. The unbound primary antibodies were washed away with PBS. The goat anti-mouse IgG H&L (Alexa Fluor^®^ 647), goat anti-rabbit IgG H&L (FITC), and DAPI (Abcam, UK) were added and incubated at 4 °C for 10 h. The signals were observed using a fluorescence microscope and analyzed by the ImageJ program. The cultured cells were centrifuged at 3283×g for 15 min to extract the supernatant. The concentrations of IL-6 and TNF-α in the supernatant were determined using Mouse IL-6 and Mouse TNF-α kit (Abcam, UK). The viability of RAW264.7 was assessed using the CCK-8 assay, following the experimental protocol outlined in **S1.5**.

## **S1.9 Nano-adsorbents *in-vivo* residue analysis**

### *S1.9.1 Determination of residual Fe element content in organs*

Take 0.6~1.2 g of PCAPAN-Fe treated rabbit heart, liver, spleen, lung, and kidney tissues. The tissues were weighed out into a pre-cleaned digestion vessel. 10 mL of 1:1 nitric acid was added to all sample vessels. The tissues were microwave digested, and the content of Fe element was determined by an inductively coupled plasma-optical emission spectrometry (ICP-OES, Avio™ 200, PerkinElmer, USA). Normal rabbit organ tissues were used as blank control.

### *S1.9.2 Fluorescence-Labeled nano-adsorbent accumulation in organs*

First, the PCAPAN-Fe was labeled with Cy5 (Cy5^®^ Conjugation Kit (Fast) - Lightning-Link^®^, Abcam, UK). The Cy5-labeled PCAPAN-Fe was then administered to rabbits following the standard procedure using the ELMAS. A control group was established using tubing with the magnetic separation module removed to simulate conditions without nano-adsorbent separation. After treatment, the rabbits were euthanized, and tissues from the heart, liver, spleen, lung, and kidney were collected, embedded in a frozen section media (Neg-50, epredia, USA), and rapidly frozen on dry ice. Subsequently, frozen sections were prepared and fixed. The tissue cytoskeleton was stained with SF488-labeled phalloidin (Solarbio, China). Finally, the frozen sections were scanned using a fluorescence slide scanner (SLIDEVIEW VS200, OLYMPUS, Japan), with tissue cells displaying a green signal and the nano-adsorbents taken up by the organs displaying a red signal.

# **S2. Supplemental figures**

**
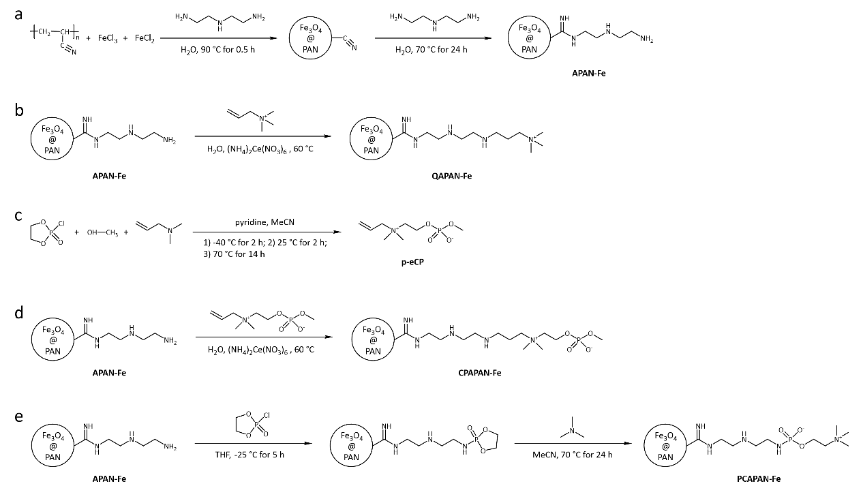
**

**Fig. S1 a** Preparation of the APAN-Fe, **b** the QAPAN-Fe, **c** prop-2-enyl choline phosphate (p-eCP), **d** the CPAPAN-Fe, and **e** the PCAPAN-Fe.


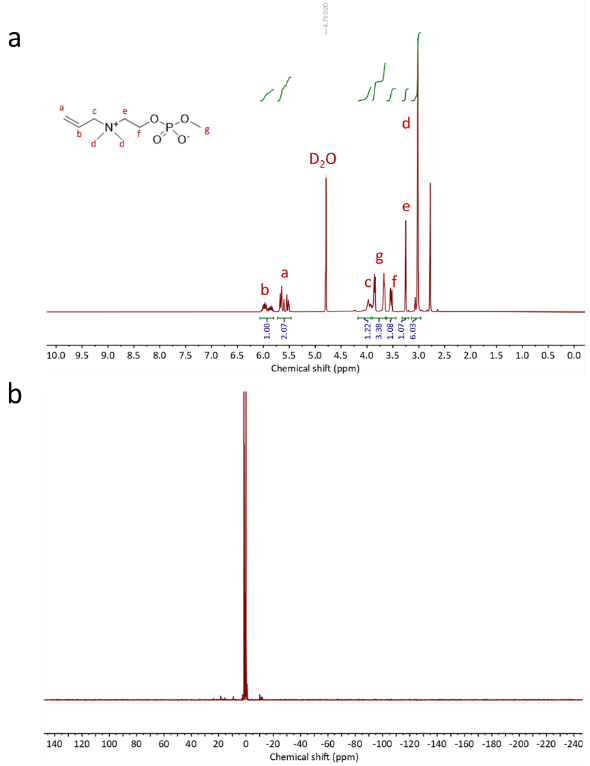


**Fig. S2 a** ^1^H-NMR and **b** ^31^P-NMR spectrum of the p-eCP.


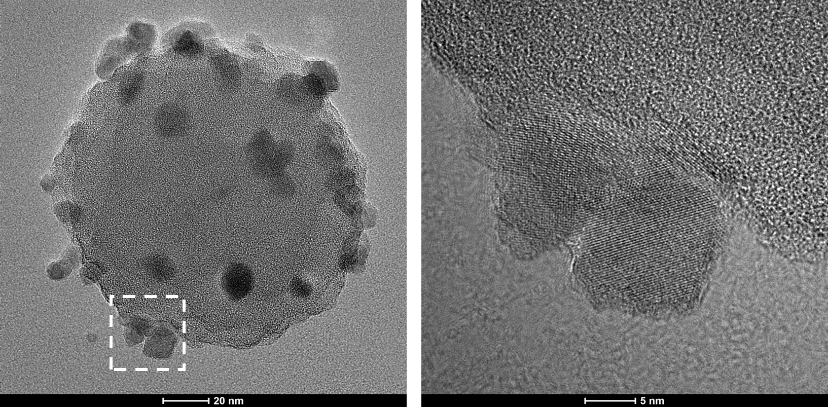


**Fig. S3** TEM images of the APAN-Fe.


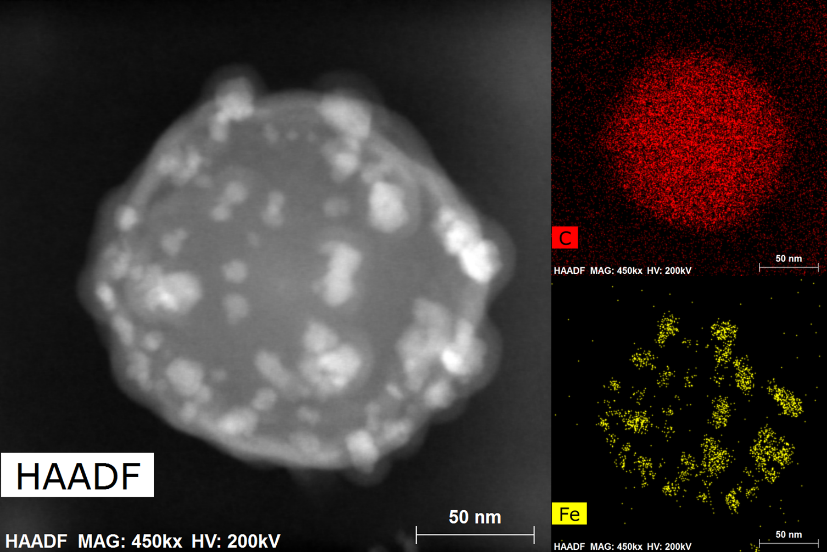


**Fig. S4** HAADF image and C, Fe species mapping of the APAN-Fe.


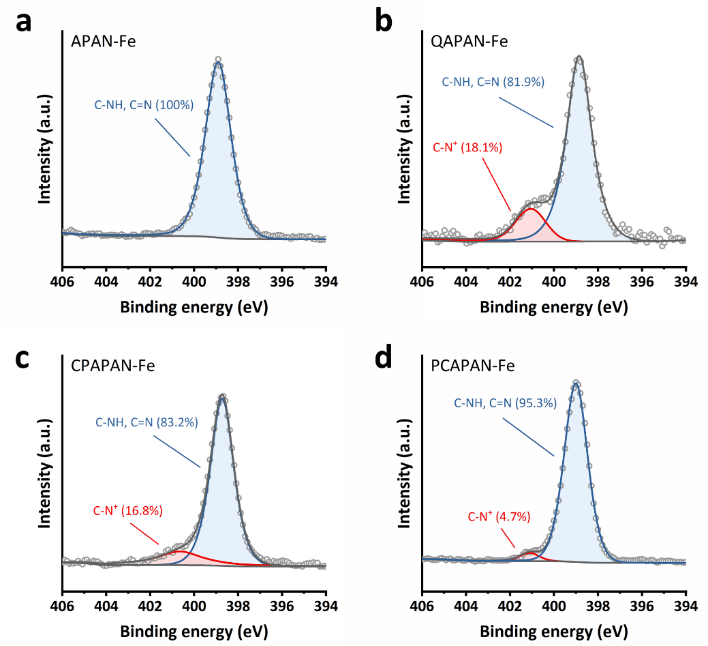


**Fig. S5** N 1s spectra of **a** the APAN-Fe, **b** the QAPAN-Fe, **c** the CPAPAN-Fe, and

**d** the PCAPAN-Fe.


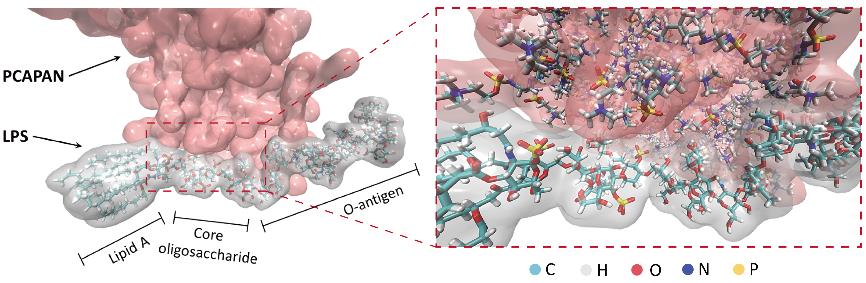


**Fig. S6** Molecular dynamics simulation of the binding transient at 50 ns of LPS and PCAPAN interaction.


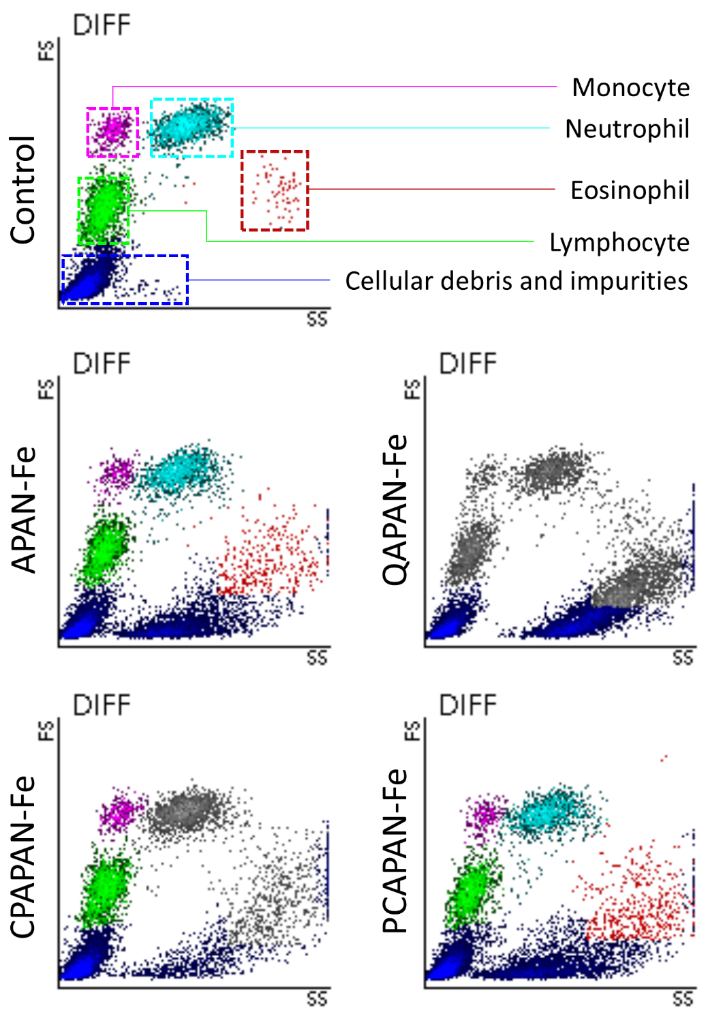


**Fig. S7** Differential white blood cell count (DIFF) scatter (forward (FS), side (SS)) charts of pristine blood and blood after incubation with the MNPs.


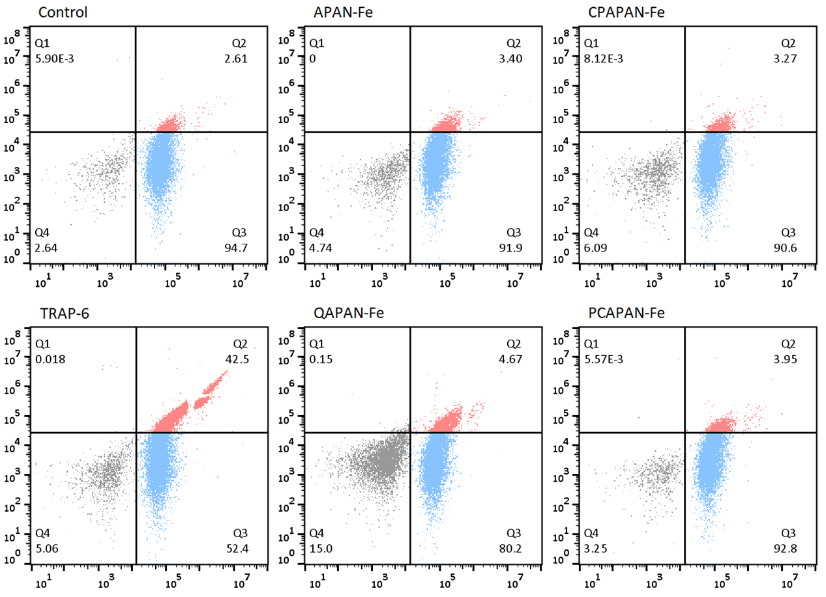


**Fig. S8** Flow cytometric analysis of anti-CD62p/anti-CD41a double staining (x-axis: CD41a, y-axis: CD62p). The PRP was treated with the MNPs (1 mg/mL) for 30 min. Untreated and TRAP-6 (0.1 mM) treated PRP were used as negative and positive controls, respectively.


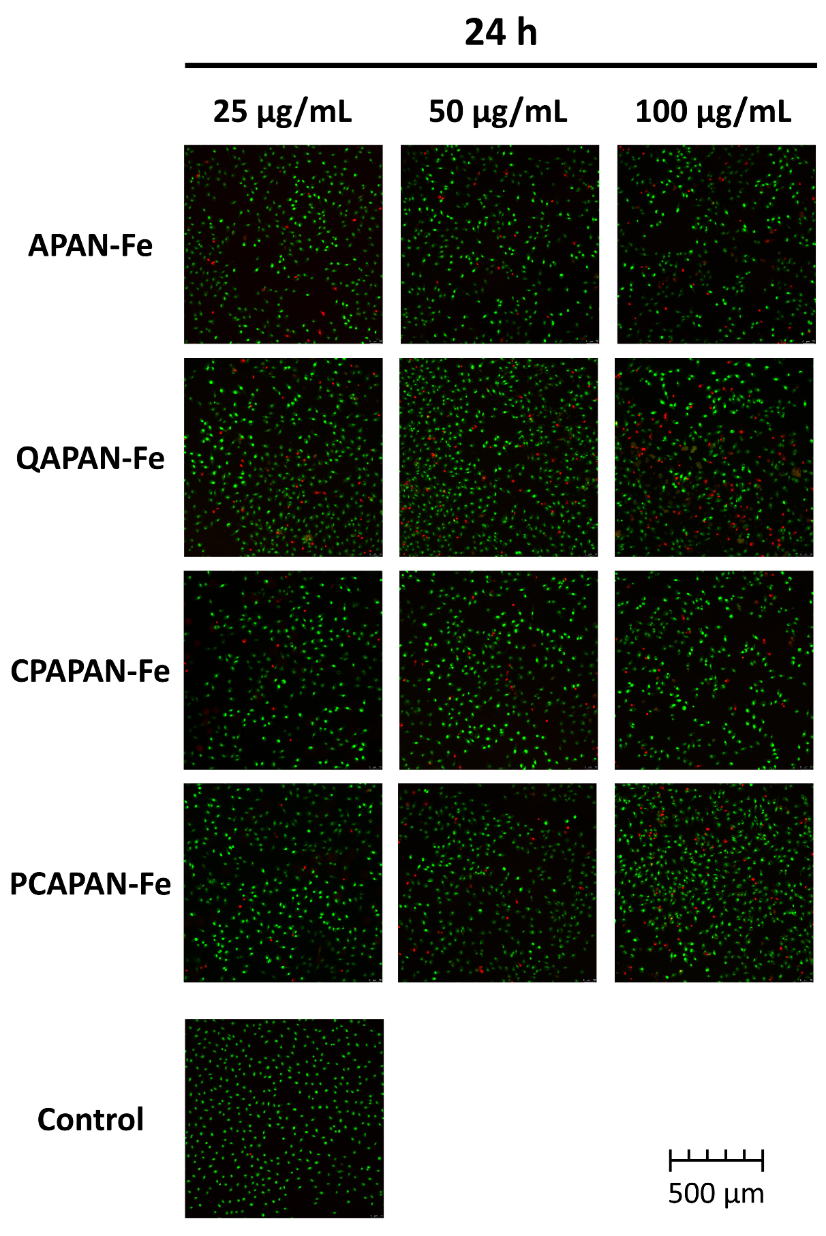


**Fig. S9** Fluorescent photographs of live/dead staining in the EA.hy926 co-cultured with different concentrations (25, 50, 100 μg/mL) of the MNPs for 24 h. (Live: green, Calcein-AM; Dead: red, Ethidium homodimer I).


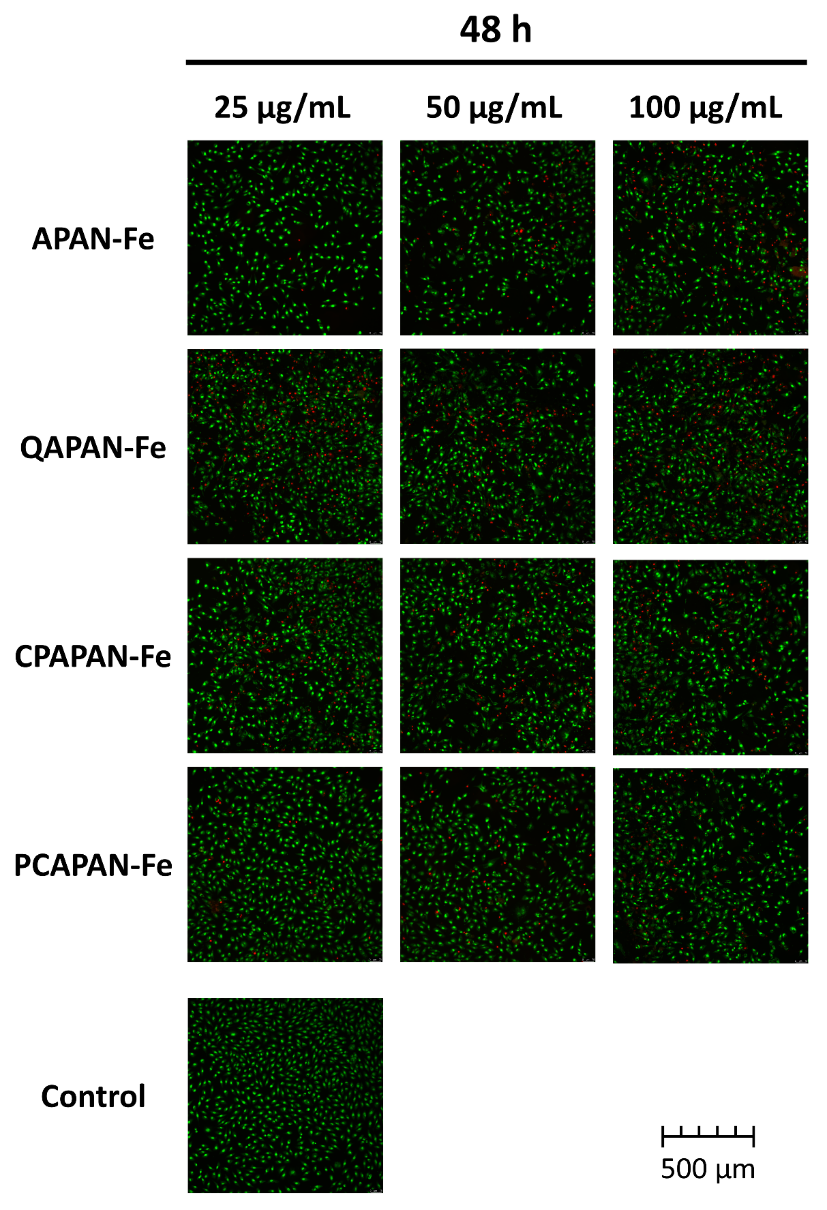


**Fig. S10** Fluorescent photographs of live/dead staining in the EA.hy926 co-cultured with different concentrations (25, 50, 100 μg/mL) of the MNPs for 48 h. (Live: green, Calcein-AM; Dead: red, Ethidium homodimer I).


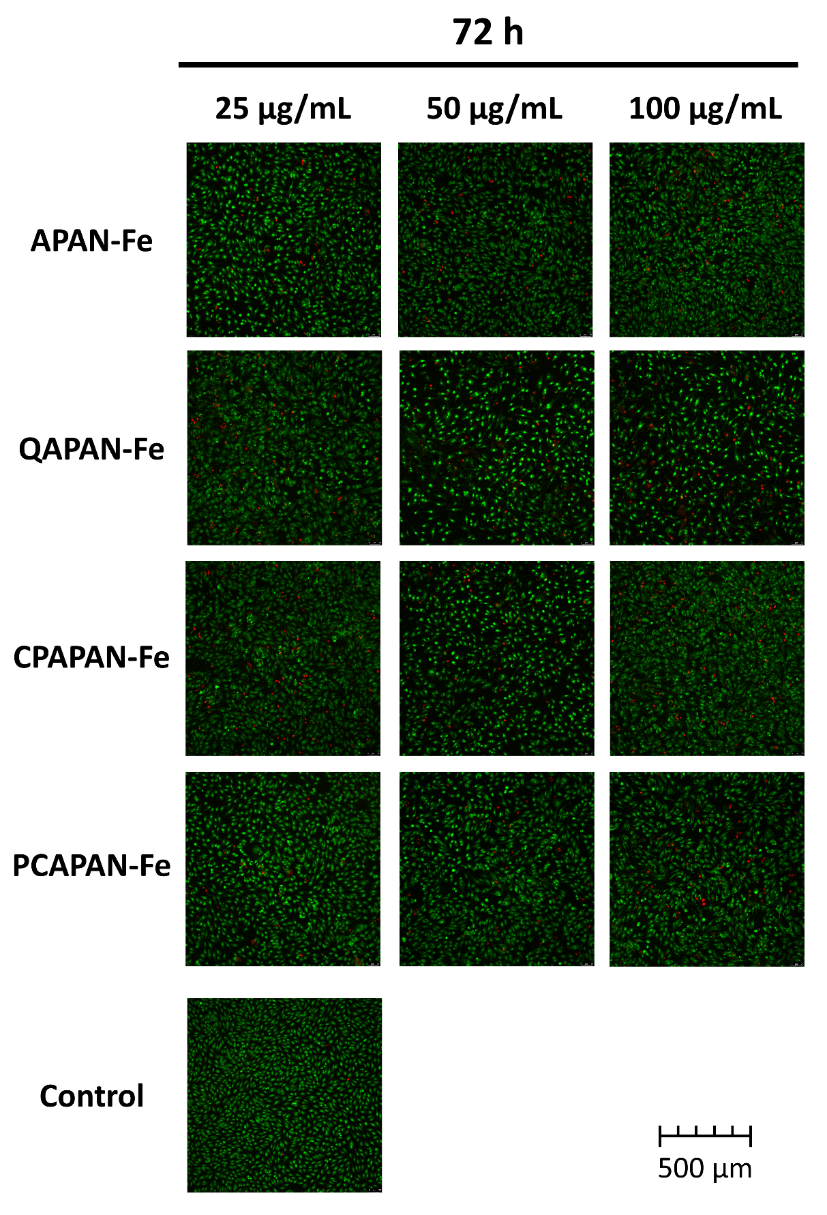


**Fig. S11** Fluorescent photographs of live/dead staining in the EA.hy926 co-cultured with different concentrations (25, 50, 100 μg/mL) of the MNPs for 72 h. (Live: green, Calcein-AM; Dead: red, Ethidium homodimer I).

**
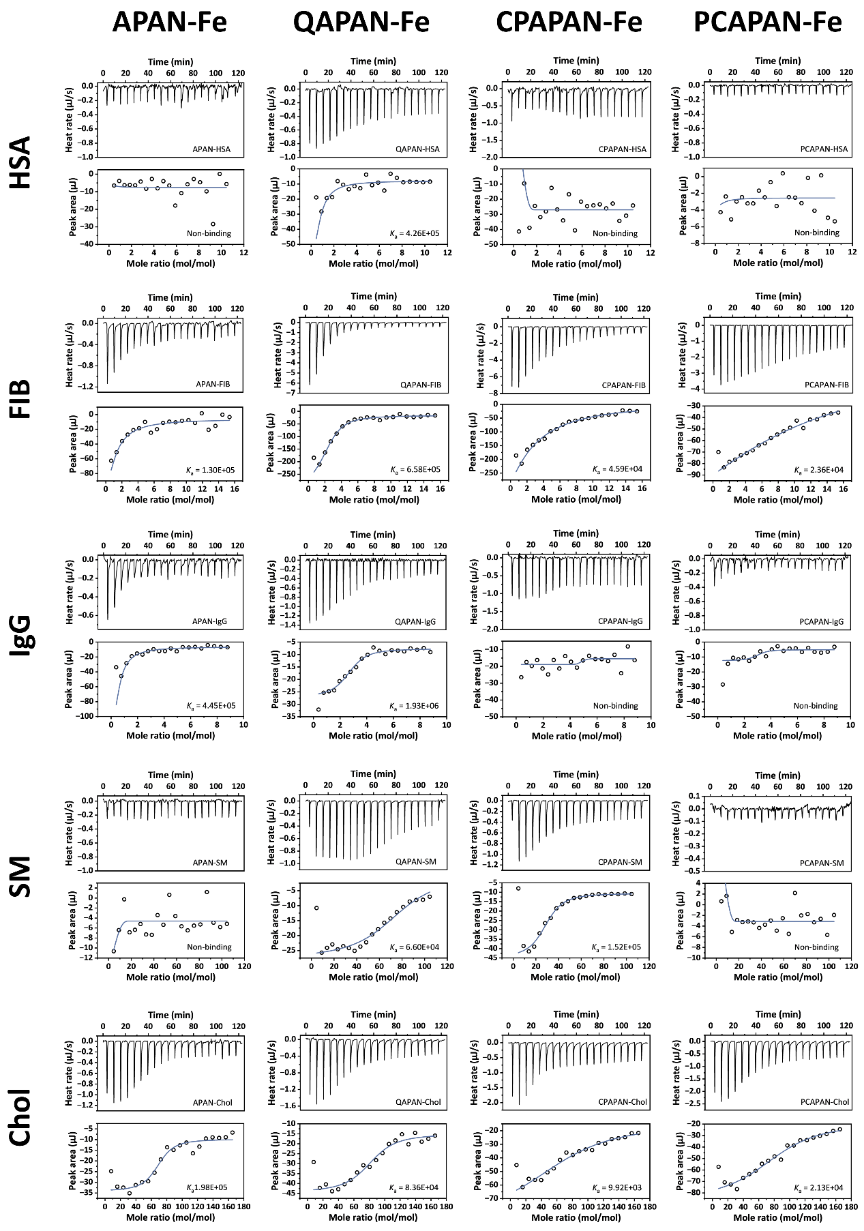
**

**Fig. S12** ITC titration and fitted curves of the MNPs binding with human serum albumin (HSA), fibrinogen (FIB), immunoglobulin G (IgG), sphingomyelin (SM) and cholesterol (Chol)

**
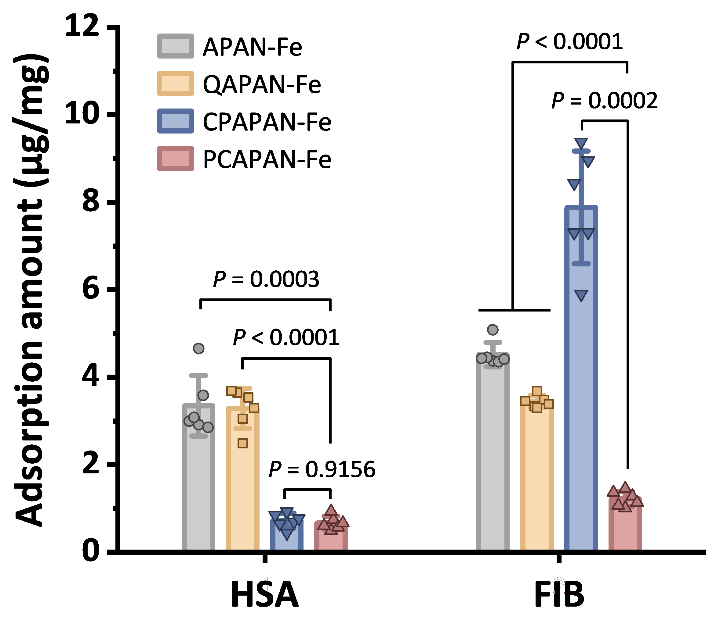
**

**Fig. S13** Adsorption amount of HSA and FIB by the APAN-Fe, the QAPAN-Fe, the CPAPAN-Fe, and the PCAPAN-Fe. (n = 6, Statistical significance was calculated via one-way ANOVA with Tukey’s post-hoc test)


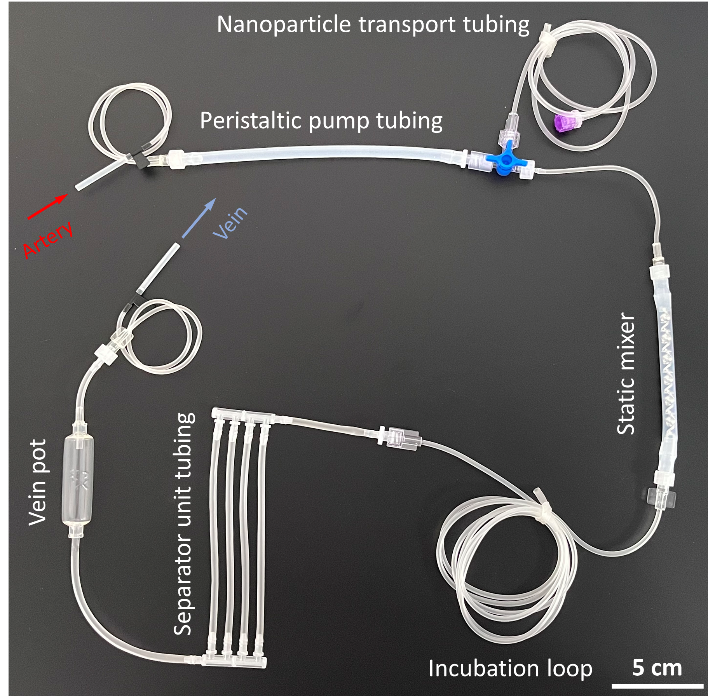


**Fig. S14** Photograph of the circulatory line for the extracorporeal LPS-targeting magnetic array system (ELMAS).


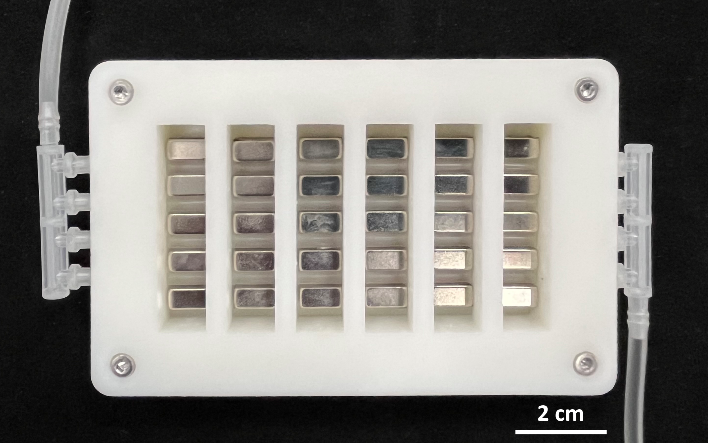


**Fig. S15** Photograph of the magnetic separator unit for the ELMAS.


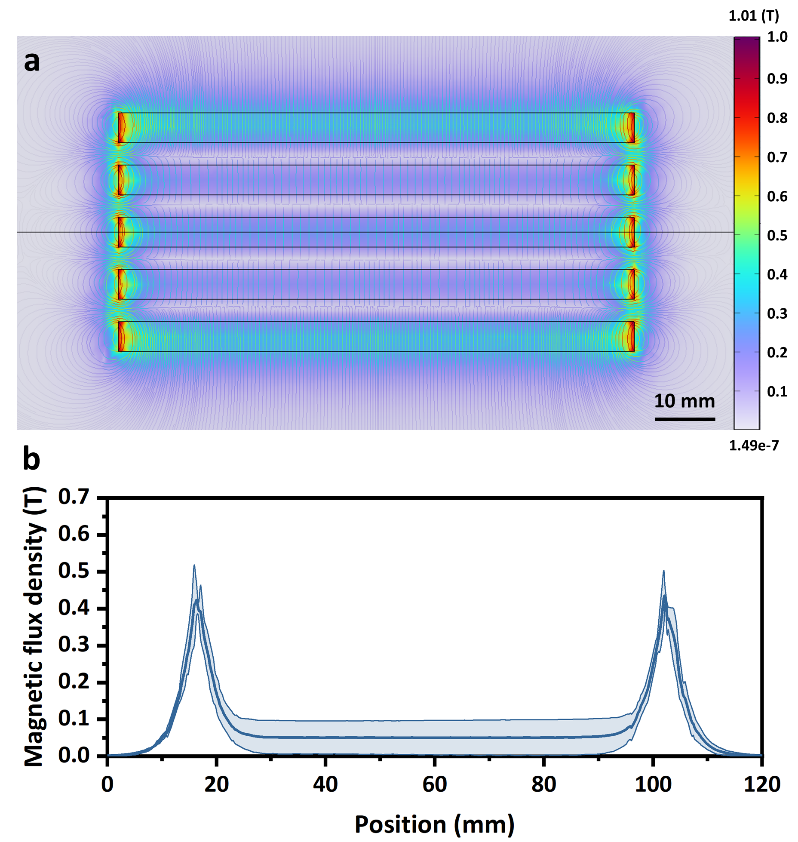


**Fig. S16** Computer simulation results using a bar magnet instead of a magnetic array as the magnetic separator unit scheme **a** Magnetic flux density distribution pattern.

**b** Values of magnetic flux density along the direction of fluid motion in the magnetic field.


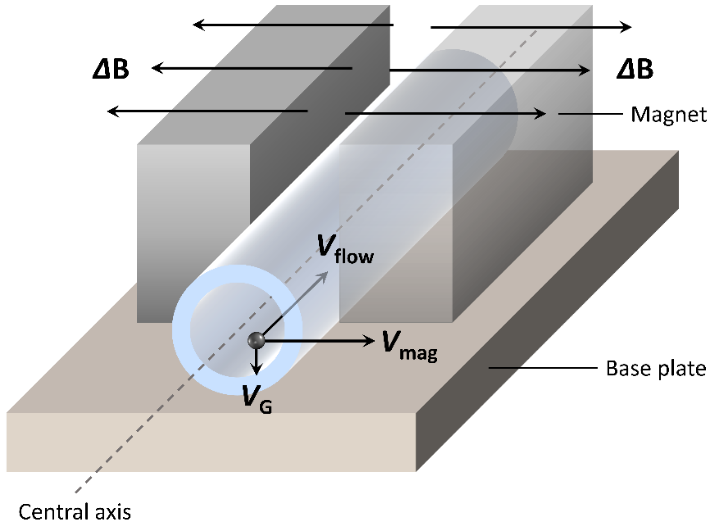


**Fig. S17** Schematic of the velocity component of the PCAPAN-Fe motion in the magnetic separation unit.


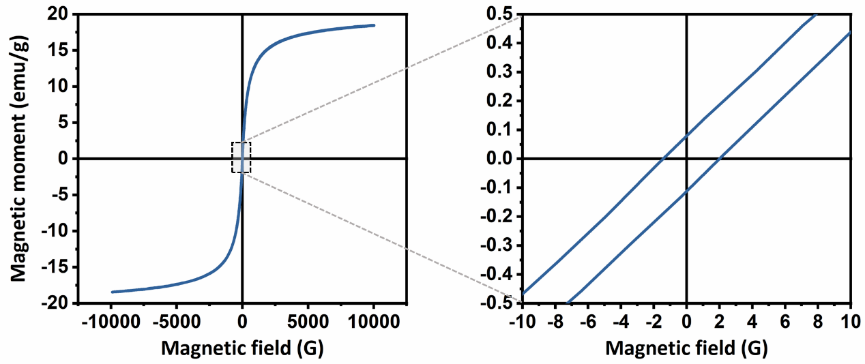


**Fig. S18** Magnetic hysteresis curve for the PCAPAN-Fe (scanning rate of 25~30 Oe/s at 37 °C. VSM instrument model: 7404, Lake Shore, USA)


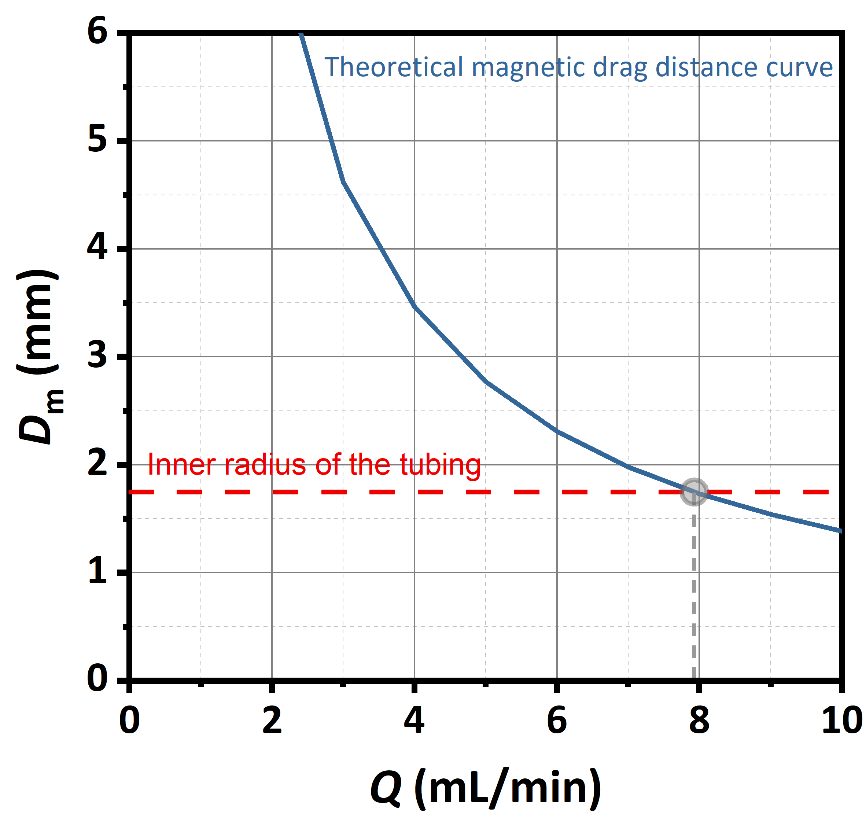


**Fig. S19** The estimated distance component along the tubing axial direction during the magnetic movement of the PCAPAN-Fe (*D*_m_). The designed inner radius of the tubing in the magnetic separator unit was 1.75 mm, Thus, the condition of magnetic recovery is *D*_m_ > 1.75 mm. Theoretical calculations show that the separator unit can effectively recover the PCAPAN-Fe at blood flow rates below 7.9 mL/min.


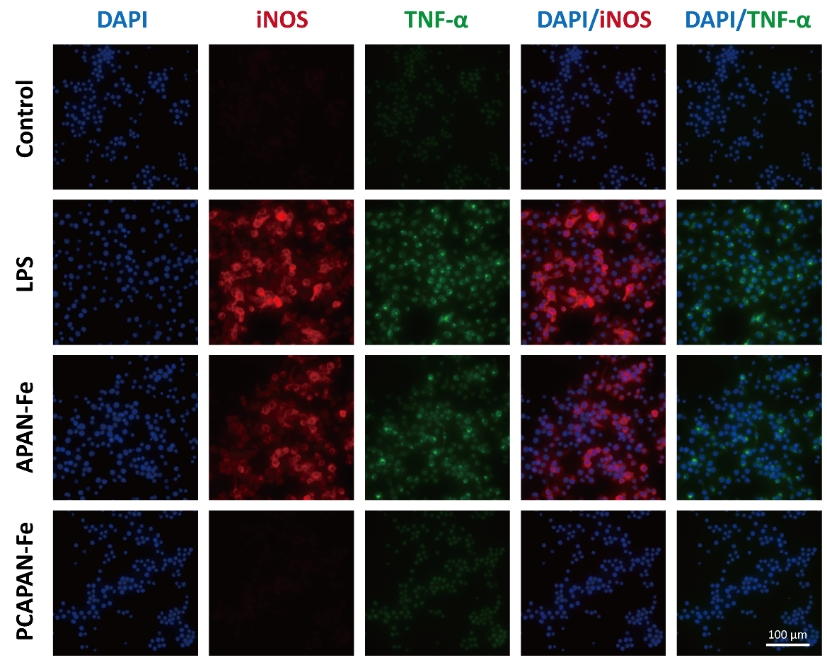


**Fig. S20** Fluorescence photographs of iNOS and TNF-*α* expression by the MNP-treated RAW264.7 after adding LPS (1 μg/mL). The MNPs (1 mg/mL) were magnetically removed after 1 h of co-culture with LPS-containing cells, and the treated cells were incubated for an additional 12 h. Normal and untreated cells as negative and positive controls, respectively. (Exposure time: blue: 120 ms, red: 500 ms, green: 500 ms)


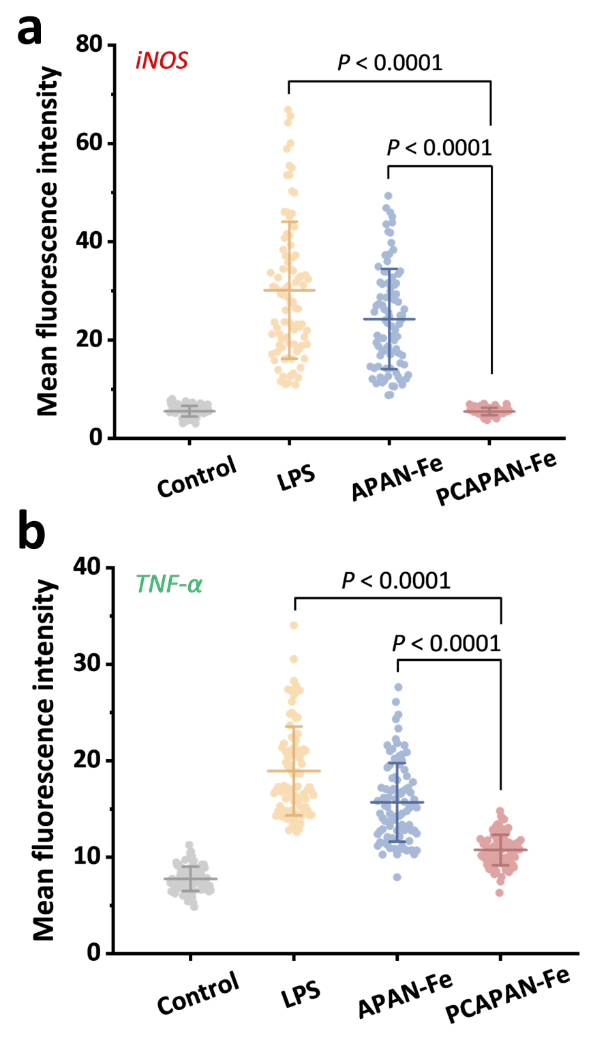


**Fig. S21** Statistics on mean fluorescence intensity of the **a** iNOS and **b** TNF-α expressed in the RAW264.7. (Three independent experiments with 30 cells each were included in the statistics. Statistical significance was calculated via one-way ANOVA with Tukey’s post-hoc test)


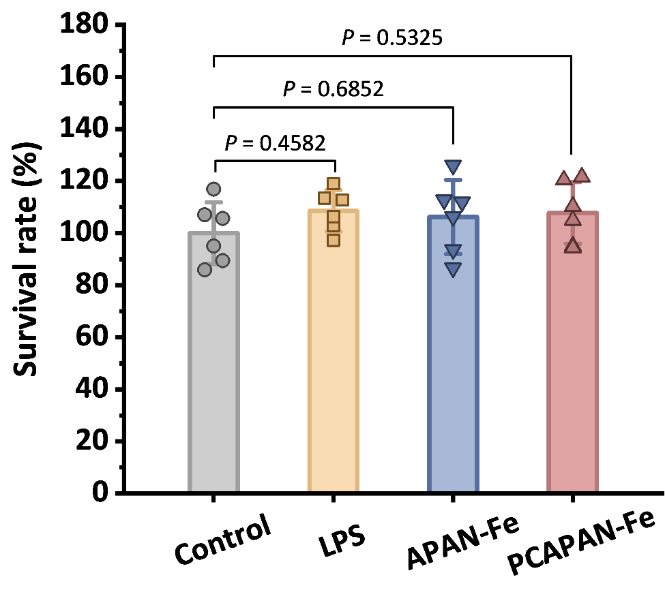


**Fig. S22** Survival of RAW264.7 after one day of co-culture with the APAN-Fe and the PCAPAN-Fe. (n = 6, Statistical significance was calculated via one-way ANOVA with Tukey’s post-hoc test)


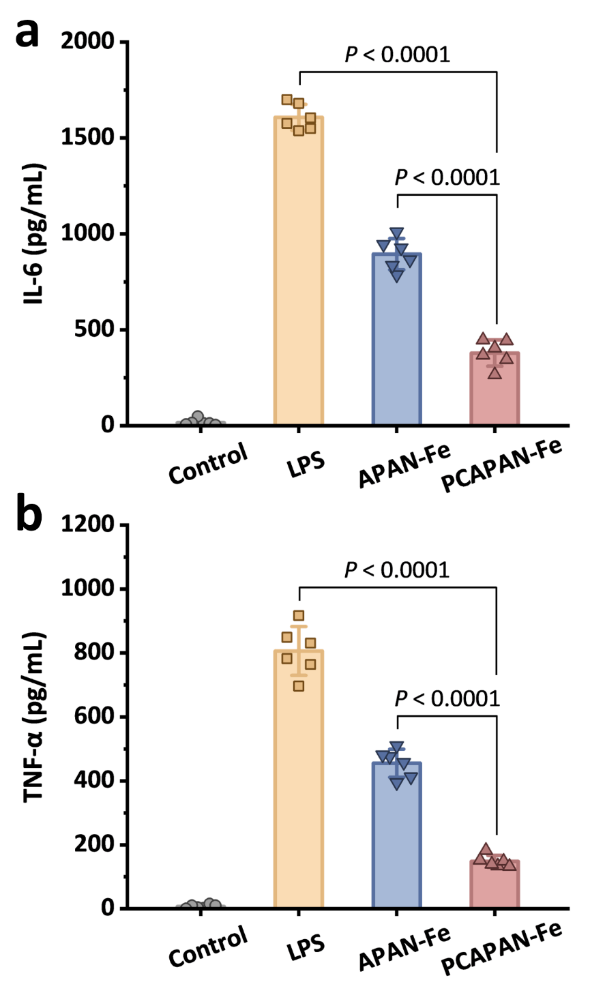


**Fig. S23** Concentrations of **a**TNF-*α* and **b** IL-6 released by the MNP-treated RAW264.7 after adding LPS, with normal and untreated cells as negative and positive controls, respectively. (n = 6, Statistical significance was calculated via one-way ANOVA with Tukey’s post-hoc test)

**
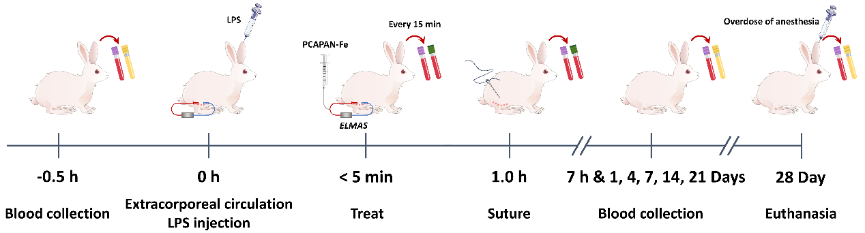
**

**Fig. S24** Experimental workflow of the PCAPAN-Fe-loaded ELMAS for early intervention in a septic rabbit model.

**
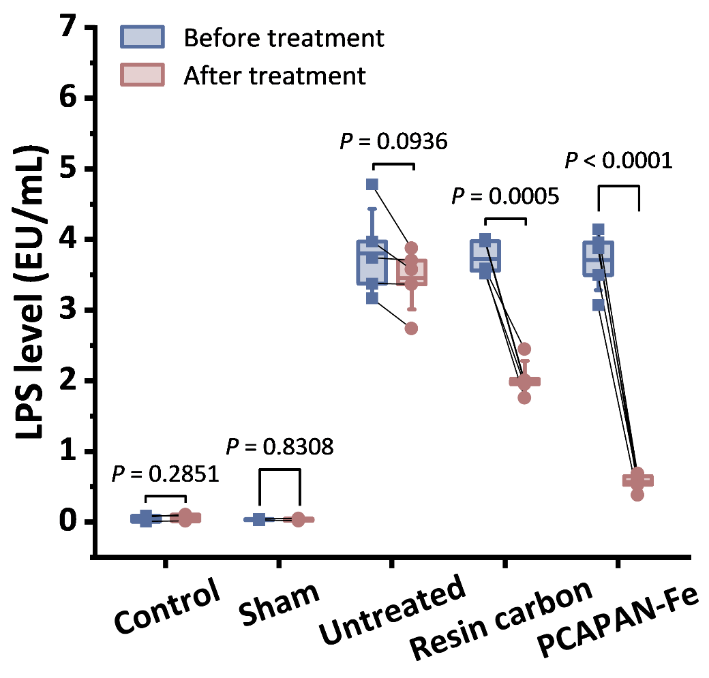
**

**Fig. S25** LPS clearance efficiency pre- and post-treatment in early-intervention septic rabbits. (n = 3 technical replicates from 5 biological replicates for each group. Statistical significance was calculated via paired two-tailed Student’s t-test)

**
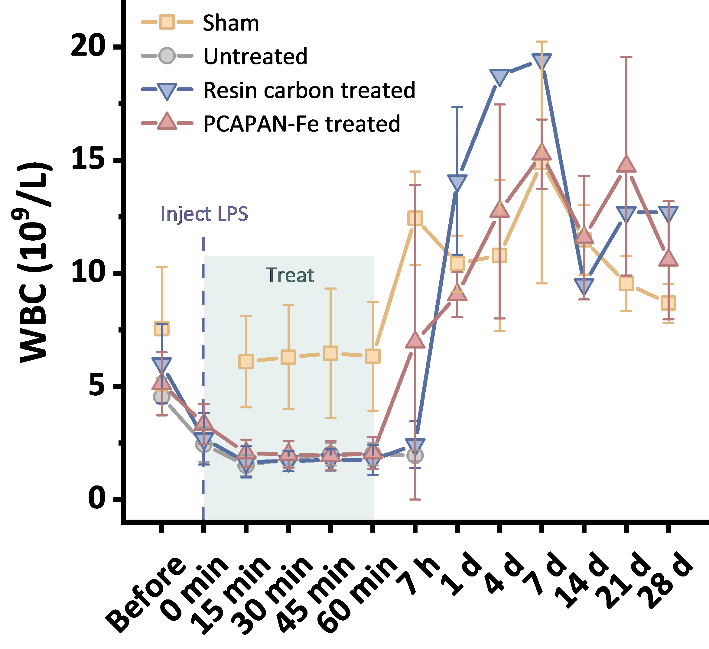
**

**Fig. S26** WBC counts of the rabbits in each early-intervention group over time.


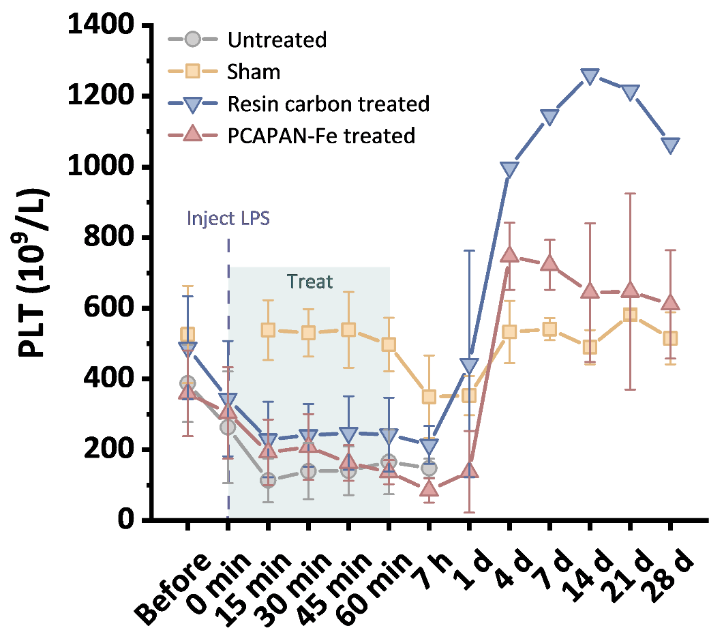


**Fig. S27** PLT counts of the rabbits in each early-intervention group over time.

**
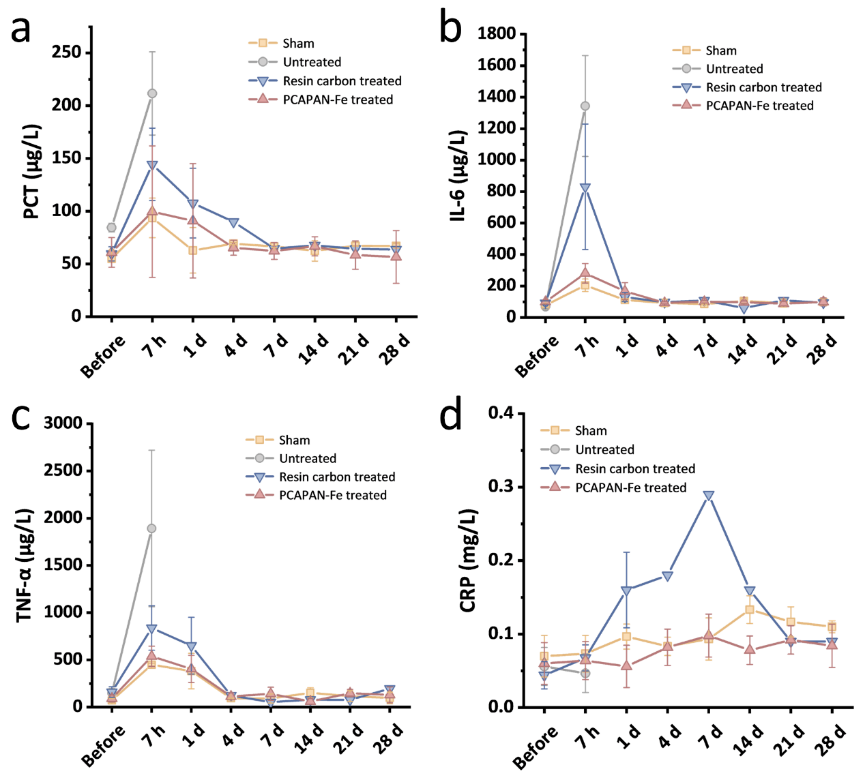
**

**Fig. S28 a** PCT, **b** IL-6, **c** TNF-α, and **d** CRP levels of the rabbits in each early-intervention group over time.


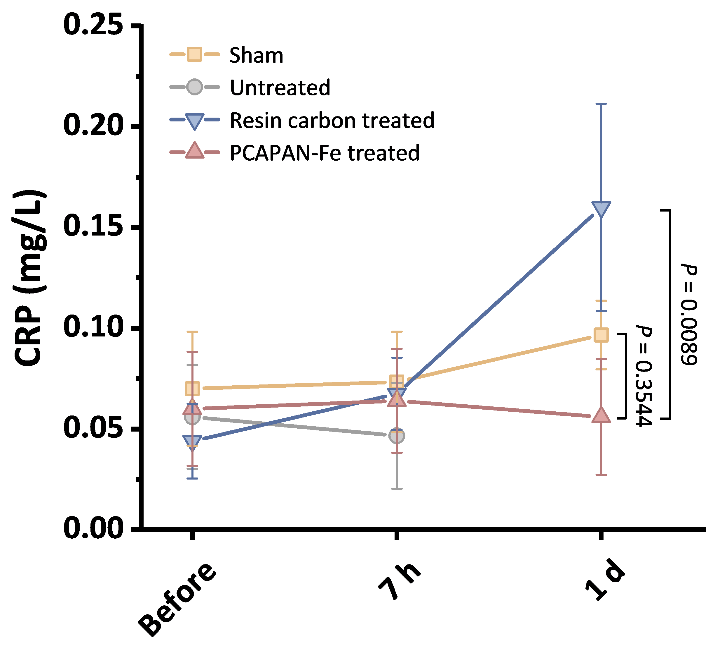


**Fig. S29** Changes in CRP levels 1-day after early sepsis intervention with analysis of significant differences (1-day survival counts:​​ Sham-operated, n = 5; Untreated, n = 0; Commercial resin carbon-treated, n = 4; PCAPAN-Fe-treated, n = 5. Statistical significance was calculated via one-way ANOVA with Tukey’s post-hoc test)

**
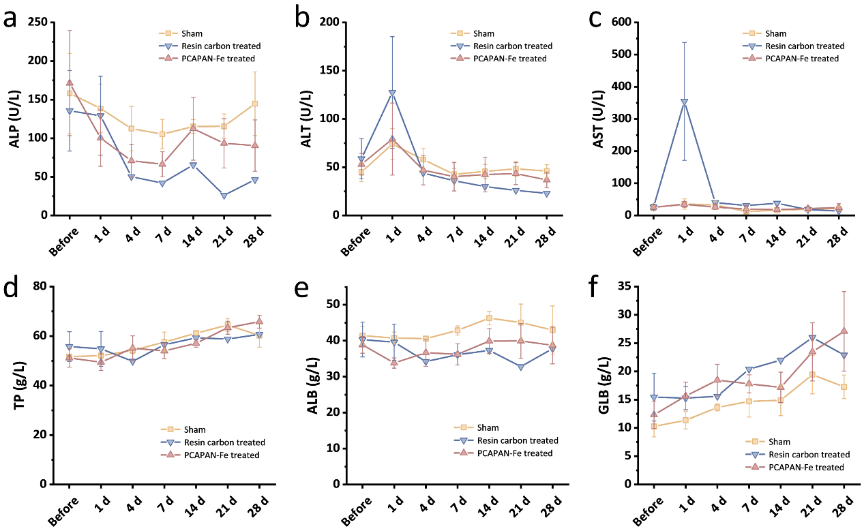
**.**Fig. S30** Biochemical indicators related to liver function at different experimental time points for rabbits in each early-intervention group. **a** ALP: alkaline phosphatase, **b** ALT: alanine transaminase, **c** AST: aspartate transaminase, **d** TP: total protein,

**e** ALB: albumin, and **f** GLB: globulin.

**
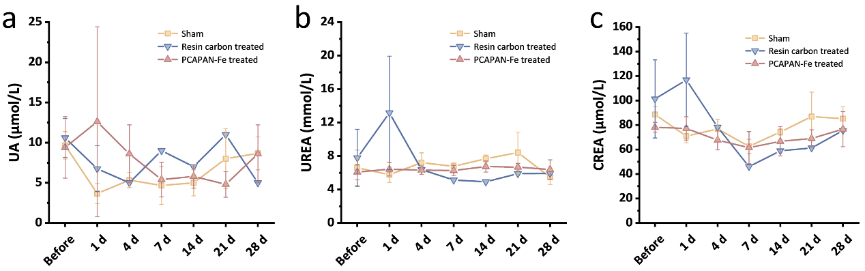
**

**Fig. S31** Biochemical indicators related to kidney function at different experimental time points for rabbits in each early-intervention group. **a** UA: uric acid, **b** UREA: urea nitrogen, **c** CREA: creatinine.


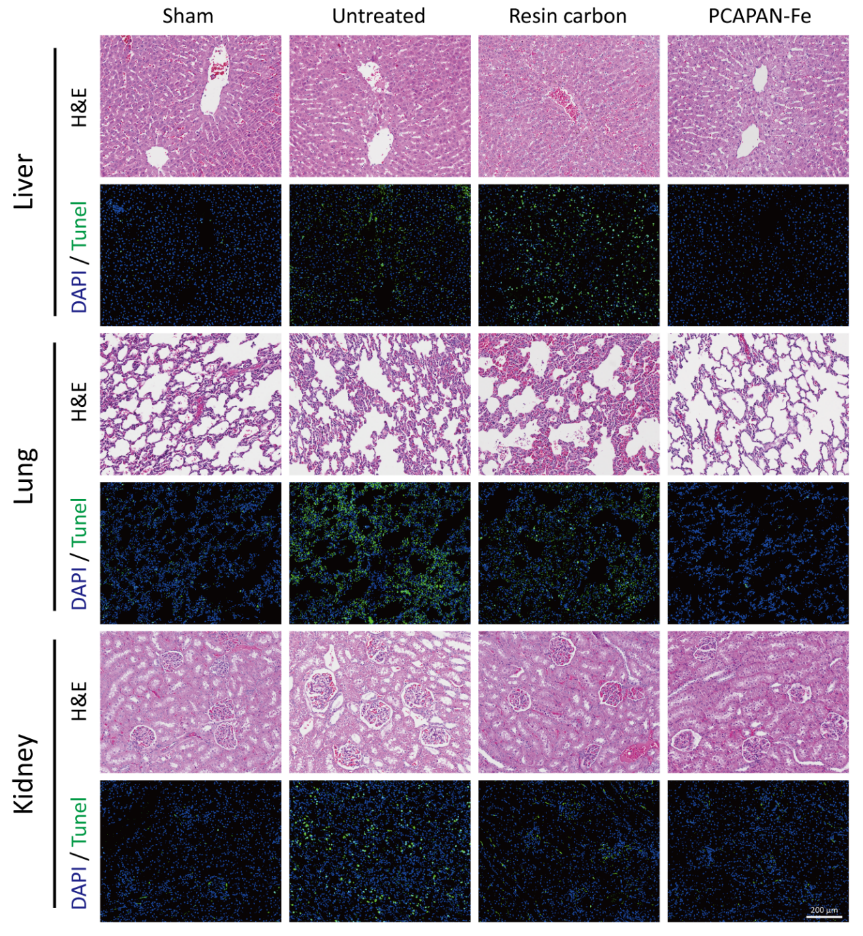


**Fig. S32** Representative H&E staining (20×) and TUNEL fluorescence (green: TUNEL; blue: DAPI; 20×) of liver, lung, and kidney tissues for the early intervention sepsis model.


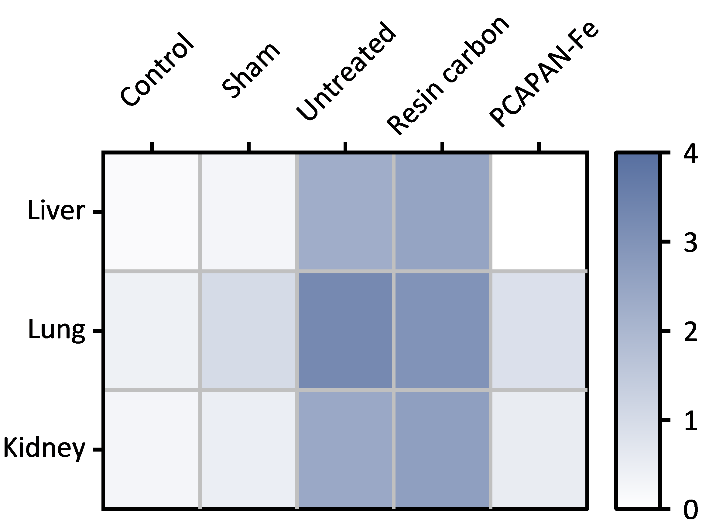


**Fig. S33** Semi-quantitative histopathological injury scoring based on H&E staining for the early intervention sepsis model (no damage = 0, abnormal field (0, 25%] = 1, (25%, 50%] = 2, (50%, 75%] = 3, (75%, 100%] = 4. Scoring was performed using a blind analysis).


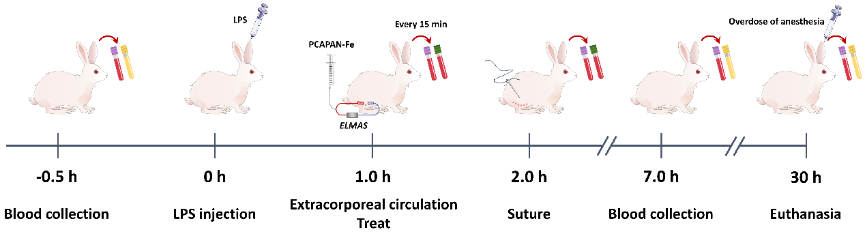


**Fig. S34** Experimental workflow of the PCAPAN-Fe-loaded ELMAS for progressive sepsis treatment in a septic rabbit model.


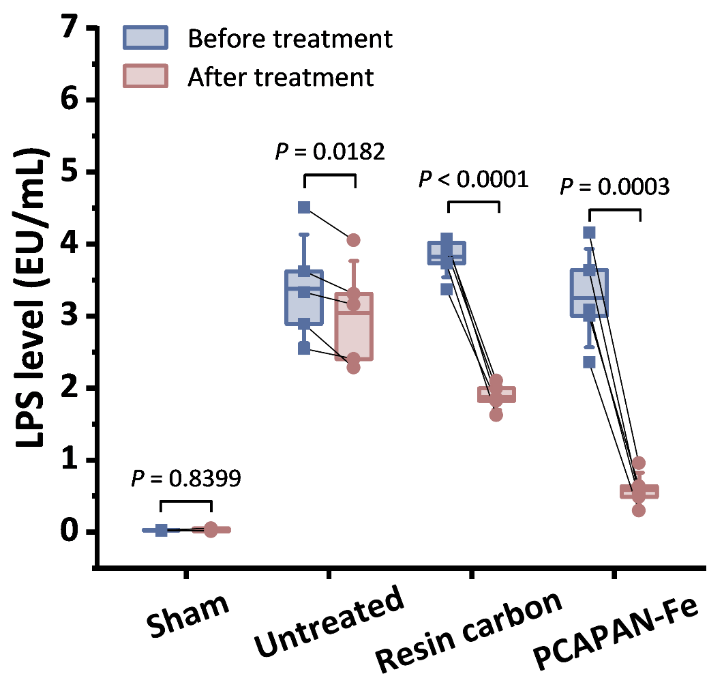


**Fig. S35** LPS clearance efficiency pre- and post-treatment in progressive-state septic rabbits. (n = 3 technical replicates from 5 biological replicates for each group. Statistical significance was calculated via paired two-tailed Student’s t-test)


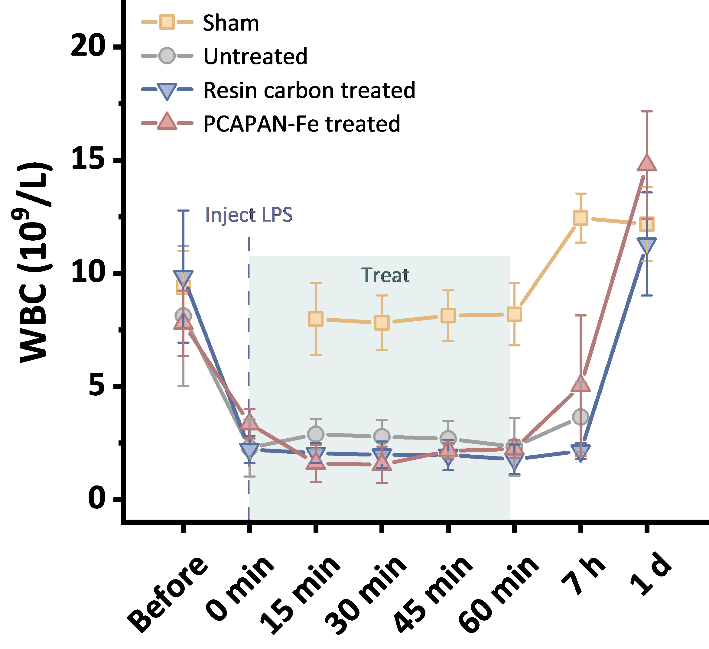


**Fig. S36** WBC counts of the rabbits in each progressive sepsis treatment group over time.

**
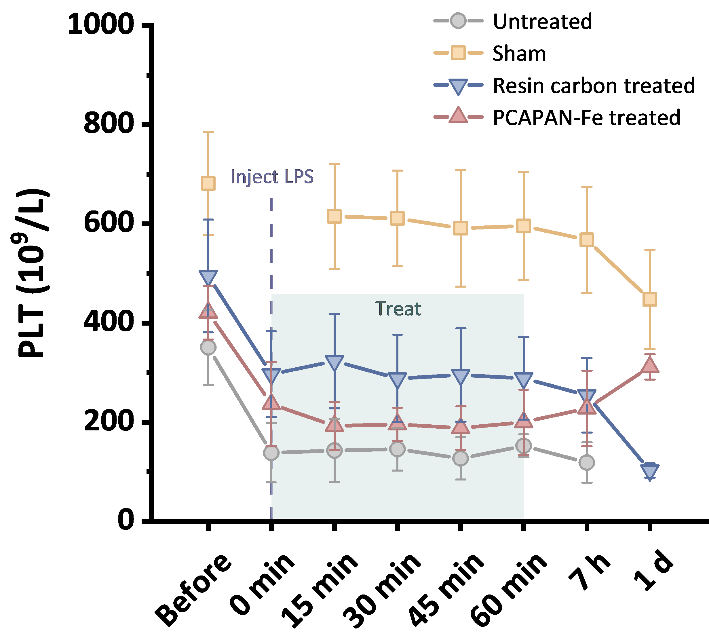
**

**Fig. S37** PLT counts of the rabbits in each progressive sepsis treatment group over time.

**
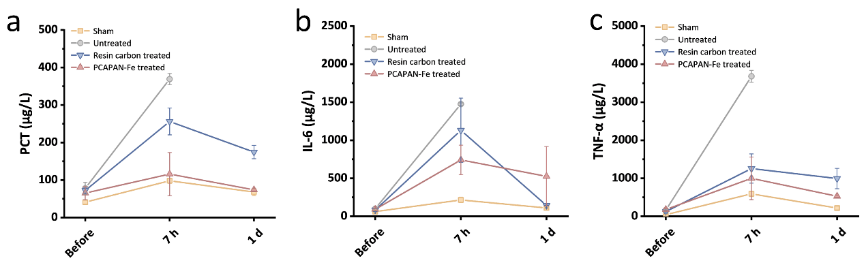
**

**Fig. S38 a** PCT, **b** IL-6, and **c** TNF-α levels of the rabbits in each progressive sepsis treatment group over time.

**
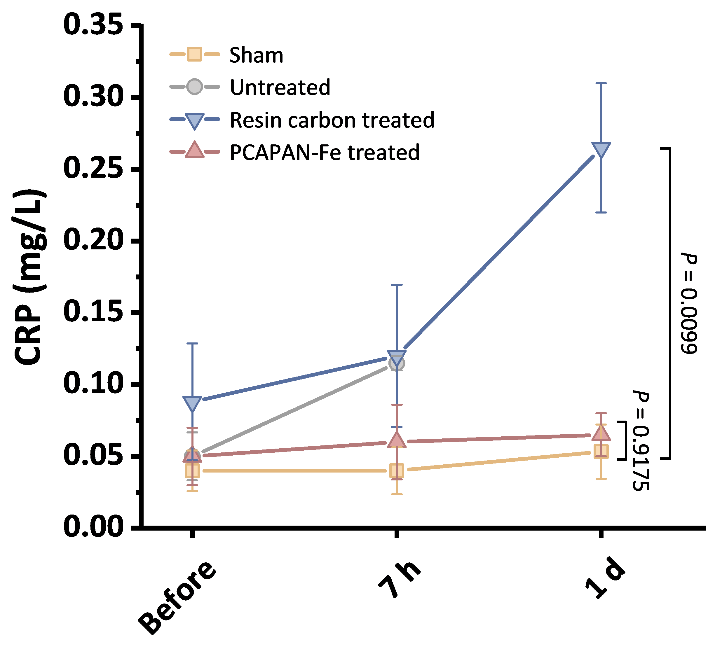
**

**Fig. S39** Changes in CRP levels 1-day of the rabbits in each progressive sepsis treatment group with analysis of significant differences (1-day survival counts:​​ Sham-operated, n = 5; Untreated, n = 0; Commercial resin carbon-treated, n = 2; PCAPAN-Fe-treated, n = 2. Statistical significance was calculated via one-way ANOVA with Tukey’s post-hoc test)

**
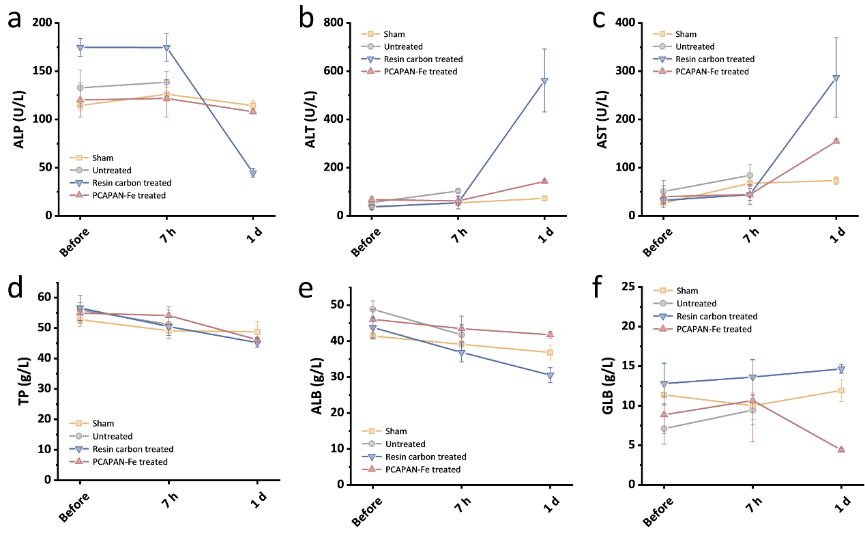
**

**Fig. S40** Biochemical indicators related to liver function at different experimental time points for rabbits in each progressive sepsis treatment group. **a** ALP: alkaline phosphatase, **b** ALT: alanine transaminase, **c** AST: aspartate transaminase, **d** TP: total protein, **e** ALB: albumin, and **f** GLB: globulin.

**
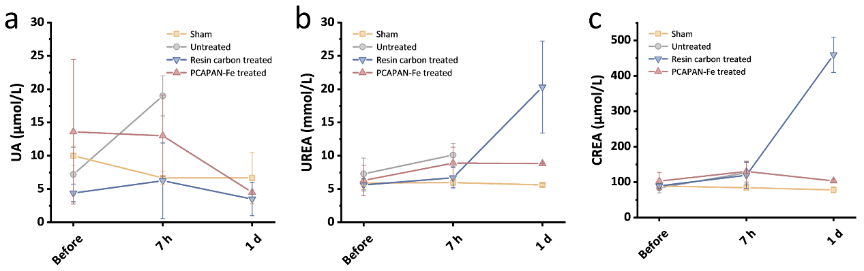
**

**Fig. S41** Biochemical indicators related to kidney function at different experimental time points for rabbits in each progressive sepsis treatment group. **a** UA: uric acid,

**b** UREA: urea nitrogen, **c** CREA: creatinine.

**
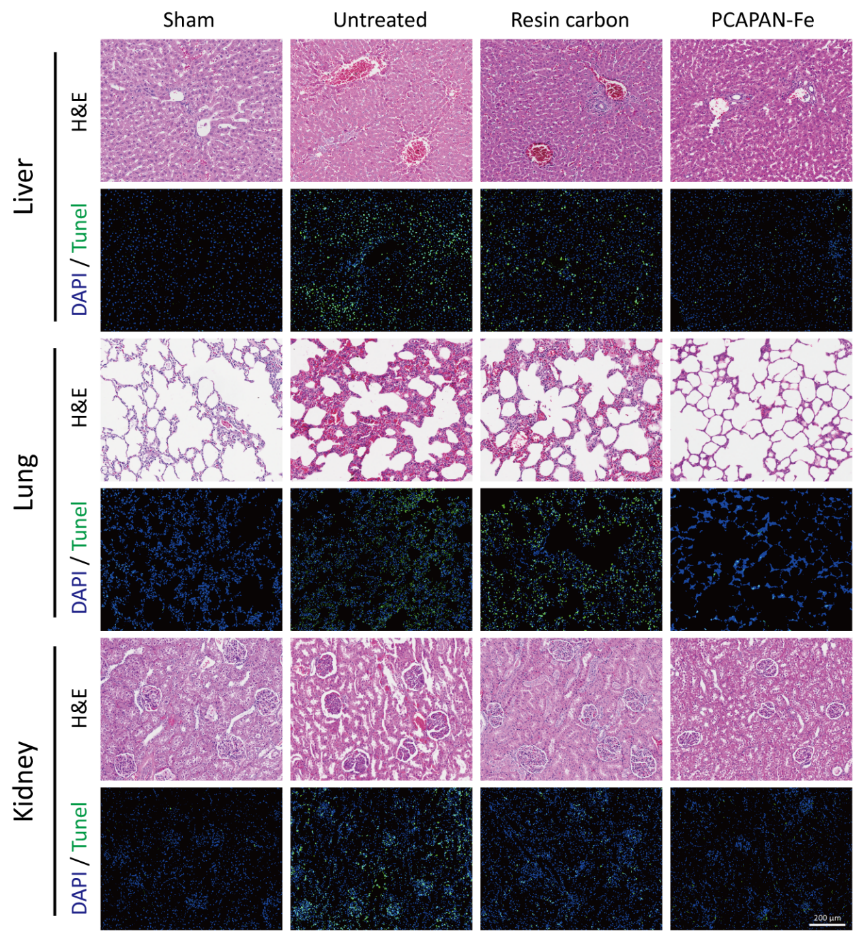
**

**Fig. S42** Representative H&E staining (20×) and TUNEL fluorescence (green: TUNEL; blue: DAPI; 20×) of liver, lung, and kidney tissues for the progressive sepsis model.


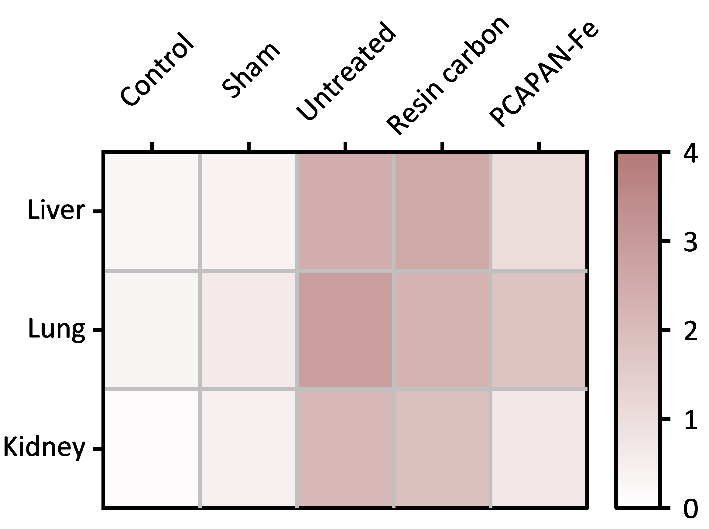


**Fig. S43** Semi-quantitative histopathological injury scoring based on H&E staining for the progressive sepsis model. (no damage = 0, abnormal field (0, 25%] = 1, (25%, 50%] = 2, (50%, 75%] = 3, (75%, 100%] = 4. Scoring was performed using a blind analysis)

**
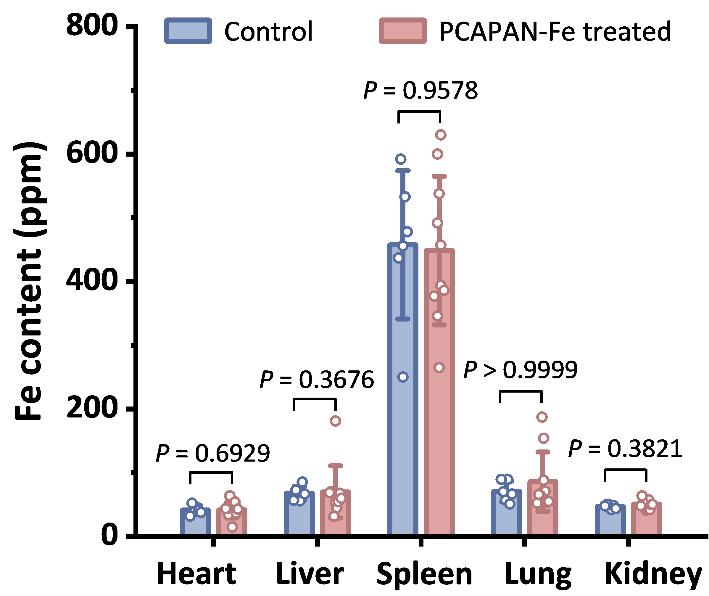
**

**Fig. S44** Fe content in major organs of the PCAPAN-Fe treated rabbits, normal rabbits as control. (Normal rabbits: n = 6, treated rabbits: n = 10. Statistical significance was calculated via unpaired two-tailed student’s t-test)

**
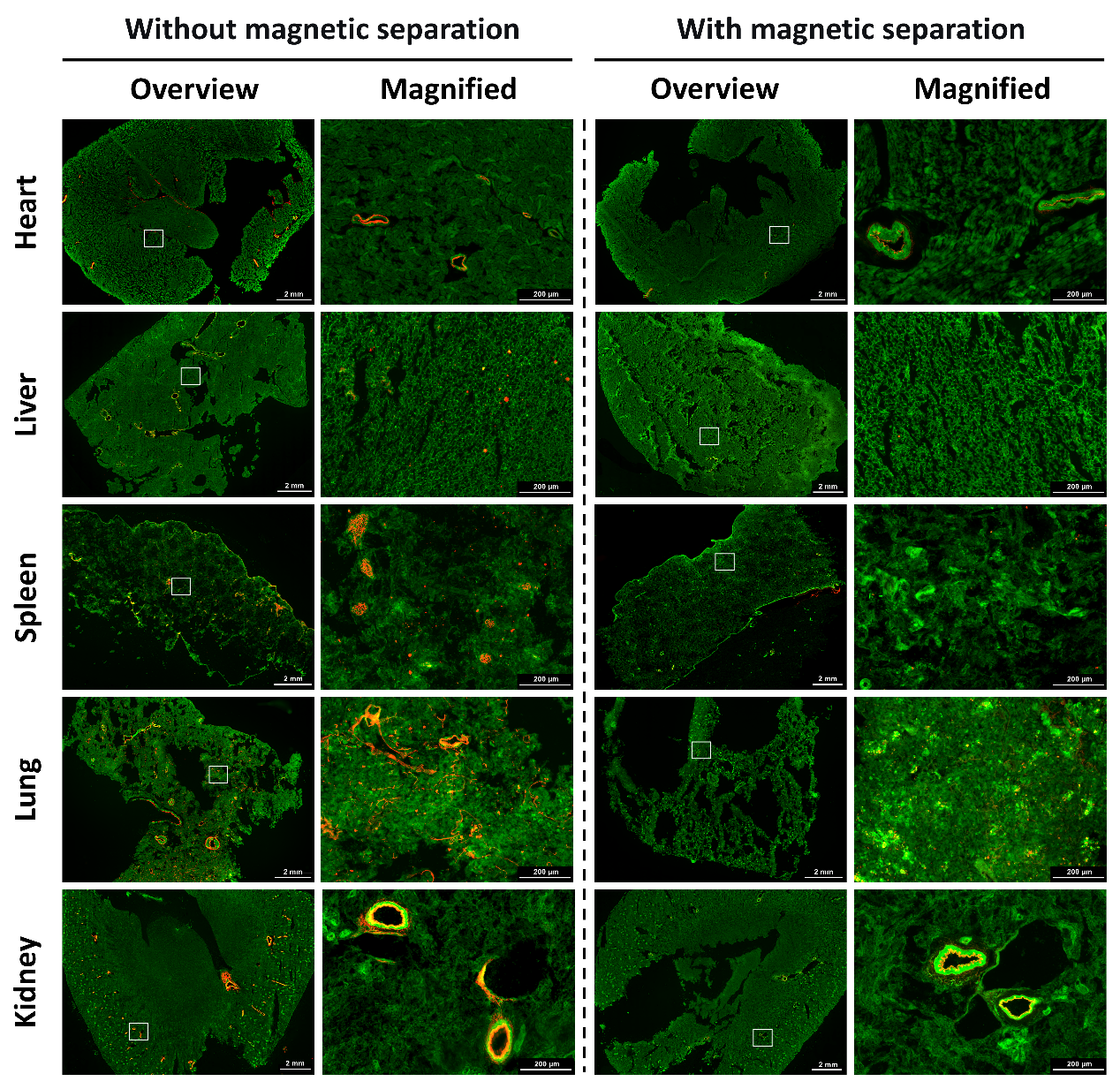
**

**Fig. S45** Uptake of the PCAPAN-Fe in rabbit organs with and without magnetic separation unit. (Green: SF488-labeled phalloidin, Red: Cy5-labeled PCAPAN-Fe; Overview: 0.5~0.7×, Magnified: 10×)


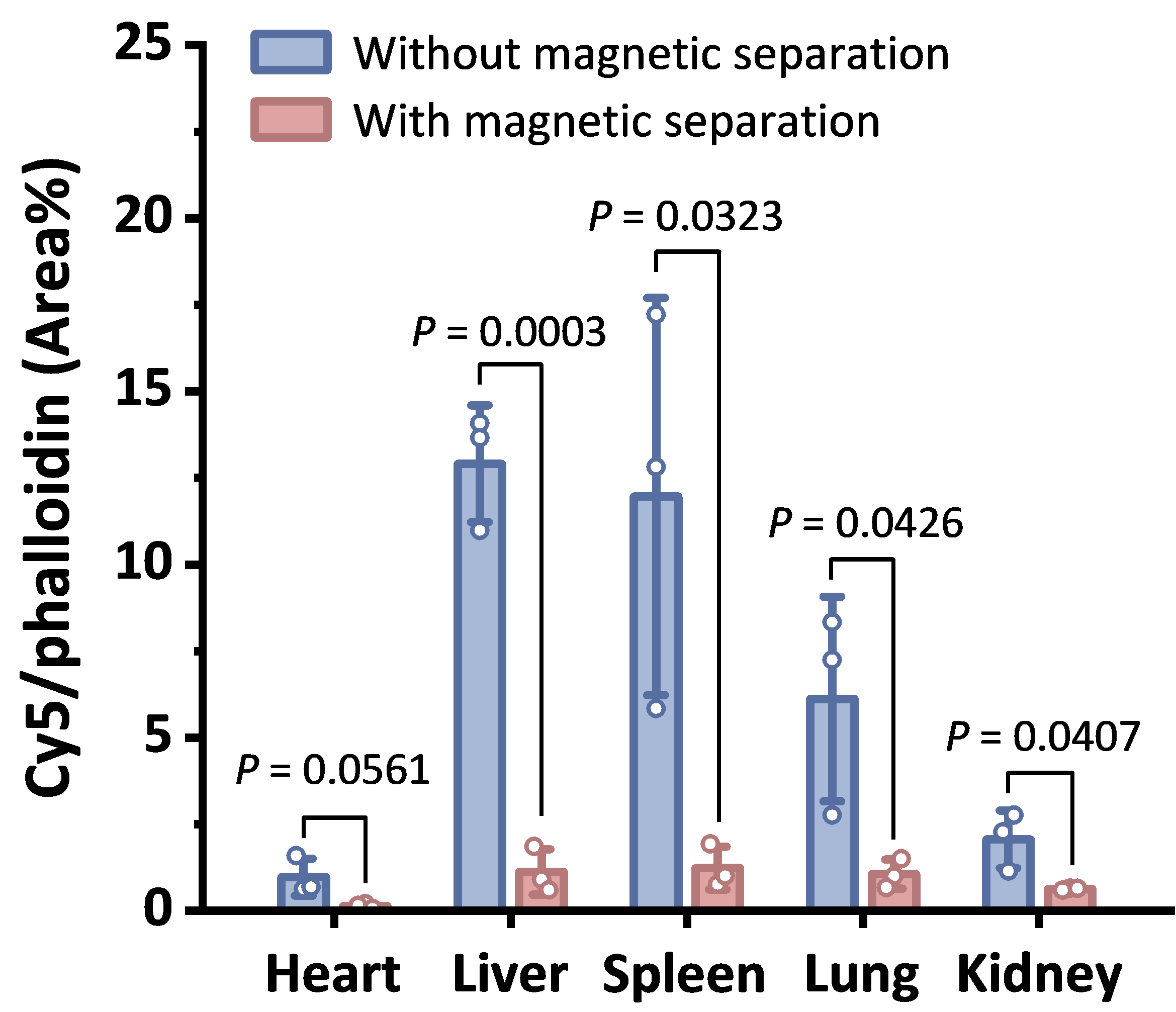


**Fig. S46** Comparison of fluorescence area percentage of the Cy5-labeled PCAPAN-Fe in tissue sections with versus without magnetic separation. (n = 3. Statistical significance was calculated via unpaired two-tailed student’s t-test)

# **S3. Supplemental tables**

**Table S1** Assignment of chemical groups to peak positions in the FTIR spectra

| **FTIR wavenumber (cm^-1^)** | **Assignment** | **Reference** |
| --- | --- | --- |
| 3402 | *ν* N-H | Setnescu *et al.*[8] |
| 2934 | *ν* C-H, *ν* C-H_2_, *ν* C-H_3_, *ν*_as_ C-H_2_ | Fu *et al.*[9] |
| 2242 | *ν* C≡N | Setnescu *et al.*[8] |
| 1737 | *ν* C-O | Setnescu *et al.*[8] |
| 1630 | *δ* N-H | Zhang *et al.*[10] |
| 1455 | *δ* C-H_2_ | Setnescu *et al.*[8] |
| 1361 | *δ_s_* C-H_3_ | Setnescu *et al.*[8] |
| 1247 | *ν* C-N | Zhang *et al.*[10] |
| 1219 | *ν* P-O | Treia *et al.*[11] |
| 1152 | *ν_s_* C-O-C | Zhang *et al.*[10] |
| 1072 | *ν_as_* O-CH_2_-C, *ρ* C-H_2_, *ρ* C-H_3_ | Fu *et al.*[9] |
| 997 | *ν_as_* P-O-C | Treia *et al.*[11] |

***** Stretching vibration (*ν*), Bending vibration (*δ*), Symmetrical bending vibration (*δ_s_*), Symmetrical stretching vibration (*ν_s_*), Antisymmetric stretching vibration (*ν_as_*), Rocking vibration (*ρ*).

**Table S2** Fitting results of the adsorption isotherm models.

| **Models** | **Parameters** | **APAN-Fe** | **QAPAN-Fe** | **CPAPAN-Fe** | **PCAPAN-Fe** |
| --- | --- | --- | --- | --- | --- |
| Langmuir isotherm | *q*_max_ (EU/mg) | 643.87 | 9030.43 | 7986.35 | 7275.23 |
|  | *K_L_* | 0.0017 | 0.0052 | 0.0028 | 0.0055 |
|  | *R*^2^ | 0.9449 | 0.9724 | 0.9699 | 0.9898 |
| Temkin isotherm | *b_T_* (J/mol) | -10.66 | 3.79 | -1.19 | 17.72 |
|  | *K_T_* (mL/EU) | 193.15 | 1661.08 | 2346.27 | 1591.20 |
|  | *R*^2^ | 0.7723 | 0.7763 | 0.8171 | 0.8134 |
| Freundlich isotherm | *n* | 1.67 | 2.39 | 1.85 | 2.24 |
|  | *K*_F_ | 6.66 | 459.68 | 148.00 | 318.81 |
|  | *R*^2^ | 0.8954 | 0.9343 | 0.9242 | 0.9733 |

**Table S3** ITC parameter fitting results of the MNPs.

| **Parameters** | **APAN-Fe** | **QAPAN-Fe** | **CPAPAN-Fe** | **PCAPAN-Fe** |
| --- | --- | --- | --- | --- |
| *K*_d_ (μM) | 3.10 ± 1.47 | 0.39 ± 0.21 | 1.34 ± 0.85 | 0.41 ± 0.36 |
| *K*_a_ (M^-1^) | 3.23×10^5^ | 2.56×10^6^ | 7.46×10^5^ | 2.44×10^6^ |
| *n* | 0.093 ± 0.372 | 1.644 ± 0.173 | 1.053 ± 0.138 | 1.141 ± 0.139 |
| *ΔH* (kJ/mol) | -499.6 ± 255.3 | 429.1 ± 116.6 | -646.2 ± 283.0 | -123.6 ± 54.51 |
| *-TΔS* (kJ/mol) | 463.58 | -467.19 | 611.33 | 85.73 |
| *ΔG* (kJ/mol) | -36.02 | -38.09 | -34.87 | -37.87 |

**Table S4** The adsorption kinetics model fitting results in DPBS solution.

| **Models** | **Parameters** | **APAN-Fe** | **QAPAN-Fe** | **CPAPAN-Fe** | **PCAPAN-Fe** |
| --- | --- | --- | --- | --- | --- |
| Pseudo-first-order model | *k_1_* (min^-1^) | 0.0242 | 0.0614 | 0.0243 | 0.0463 |
|  | *q_e_* (EU/mg) | 31.862 | 104.914 | 99.564 | 105.380 |
|  | *R*^2^ | 0.9783 | 0.9923 | 0.9815 | 0.9907 |
| Pseudo-second-order model | *k_2_* (mg·EU^-1^·min^-1^) | 5.837×10^-4^ | 8.195×10^-4^ | 1.887×10^-4^ | 5.070×10^-4^ |
|  | *q_e_* (EU/mg) | 40.365 | 115.859 | 125.899 | 120.453 |
|  | *R*^2^ | 0.9626 | 0.9699 | 0.9684 | 0.9635 |
| Weber-Morris model | *k_W-M_* (EU·mg^-1^·min^-1/2^) | 4.814 | 15.638 | 14.492 | 14.463 |
|  | *R*^2^ | 0.9647 | 0.9158 | 0.9809 | 0.9404 |
| Film mass transfer model | *A* (min^-1^) | 0.0332 | 0.0830 | 0.0377 | 0.0682 |
|  | *k_f_* (nm/s) | 6.519 | 55.146 | 22.933 | 45.364 |
|  | *R*^2^ | 0.9751 | 0.9624 | 0.9770 | 0.9519 |

**Table S5** The adsorption kinetics model fitting results in human whole blood.

| **Models** | **Parameters** | **APAN-Fe** | **QAPAN-Fe** | **CPAPAN-Fe** | **PCAPAN-Fe** |
| --- | --- | --- | --- | --- | --- |
| Pseudo-first-order model | *k_1_* (min^-1^) | 0.0269 | 0.0384 | 0.0316 | 0.0462 |
|  | *q_e_* (EU/mg) | 6.8833 | 19.937 | 12.473 | 20.409 |
|  | *R*^2^ | 0.9724 | 0.9952 | 0.9946 | 0.9888 |
| Pseudo-second-order model | *k_2_* (mg·EU^-1^·min^-1^) | 0.0037 | 0.0020 | 0.0023 | 0.0026 |
|  | *q_e_* (EU/mg) | 8.248 | 23.364 | 15.089 | 23.325 |
|  | *R*^2^ | 0.9846 | 0.9766 | 0.9817 | 0.9630 |
| Weber-Morris model | *k_W-M_* (EU·mg^-1^·min^-1/2^) | 0.562 | 2.775 | 1.788 | 3.626 |
|  | *R*^2^ | 0.9713 | 0.9668 | 0.9781 | 0.9968 |
| Film mass transfer model | *A* (min^-1^) | 0.0310 | 0.0559 | 0.0288 | 0.0693 |
|  | *k_f_* (nm/s) | 7.039 | 35.924 | 11.750 | 45.450 |
|  | *R*^2^ | 0.9316 | 0.9750 | 0.9776 | 0.9634 |

**Table S6** Comparative analysis of protein and LPS adsorption capacities: literature-reported adsorbents versus the current study.

| **Adsorbent** | **Type** | **LPS adsorption capacity (EU/g)** | **Protein adsorption capacity (mg/g)** | **Reference** |
| --- | --- | --- | --- | --- |
| PEI/PDA@PSF ENMs | Nanofiber | 10142 | 97.8 | Dou *et al.*[12] |
| PES/PDA-Ser | Nanofiber | 1280 | 32.98 | Zhao *et al.*[13] |
| GPESDM | Nanofiber | 28417 | 40 | Bao *et al.*[14] |
| Nanofiber sponge | Nanofiber | 17889 | 73.2 | Huang *et al.*[15] |
| 90CA10SF | Nanofiber | 859.3 | 8.45 | Cai *et al.*[16] |
| C_2_-K_1_ | Bead | 202.8 | 0.153 | Li *et al.*[17] |
| ET-CCSPD | Bead | 272.5 | 0.191 | Liu *et al.*[1] |
| PS-Hep-Phe | Bead | 25.15 | 16.07 | Dang *et al.*[18] |
| PVA-AMWCNT | Bead | 114 | 2.92 | Zhong *et al.*[19] |
| CPG-Ln-MSs | Bead | 455.3 | 0.128 | Wei *et al.*[20] |
| PGA-P-H | Bead | 2659.1 | 0.75 | Yang *et al.*[21] |
| PAI | Bead | 433.5 | 0.52 | Ling *et al.*[22] |
| P-Ce-PMB | Bead | 9.2 | 0.53 | Im *et al.*[23] |
| PCAPAN-Fe | Nanoparticle | 7.3×10^6^ | 0.65 | **This work** |

# **Reference**

[1] Liu X, Xu T, Jiang C, Li Y, Su B, Zhao W and Zhao C. Ultraporous polyquaternium-carboxylated chitosan composite hydrogel spheres with anticoagulant, antibacterial, and rapid endotoxin removal profiles for sepsis treatment. *Biomacromolecules*. 2022;23(9):3728-3742. <https://doi.org/https://doi.org/10.1021/acs.biomac.2c00583>

[2] Duff DG, Ross SM and Vaughan DH. Adsorption from solution: An experiment to illustrate the langmuir adsorption isotherm. *J Chem Educ*. 1988;65(9):815. <https://doi.org/https://doi.org/10.1021/ed065p815>

[3] Johnson RD and Arnold FH. The temkin isotherm describes heterogeneous protein adsorption. *Biochim Biophys Acta, Protein Struct Mol Enzymol*. 1995;1247(2):293-297. <https://doi.org/https://doi.org/10.1016/0167-4838(95)00006-G>

[4] Foo KY and Hameed BH. Insights into the modeling of adsorption isotherm systems. *Chem Eng J*. 2010;156(1):2-10. <https://doi.org/https://doi.org/10.1016/j.cej.2009.09.013>

[5] Wang J and Guo X. Adsorption kinetic models: Physical meanings, applications, and solving methods. *J Hazard Mater*. 2020;390(122156. <https://doi.org/https://doi.org/10.1016/j.jhazmat.2020.122156>

[6] Wu F-C, Tseng R-L and Juang R-S. Initial behavior of intraparticle diffusion model used in the description of adsorption kinetics. *Chem Eng J*. 2009;153(1-3):1-8. <https://doi.org/https://doi.org/10.1016/j.cej.2009.04.042>

[7] Yao C and Chen T. A film-diffusion-based adsorption kinetic equation and its application. *Chem Eng Res Des*. 2017;119(87-92. <https://doi.org/https://doi.org/10.1016/j.cherd.2017.01.004>

[8] Setnescu R, Jipa S, Setnescu T, Kappel W, Kobayashi S and Osawa Z. Ir and x-ray characterization of the ferromagnetic phase of pyrolysed polyacrylonitrile. *Carbon*. 1999;37(1):1-6. <https://doi.org/https://doi.org/10.1016/S0008-6223(98)00168-7>

[9] Fu D, Lu Y, Peng Z and Zhong W. A zwitterionic hydrogel with a surprising function of increasing the ionic conductivity of alkali metal chloride or sulfuric acid water-soluble electrolyte. *J Mater Chem A*. 2023;<https://doi.org/https://doi.org/10.1039/D3TA06577D>

[10] Zhang J, Xue Q, Pan X, Jin Y, Lu W, Ding D and Guo Q. Graphene oxide/polyacrylonitrile fiber hierarchical-structured membrane for ultra-fast microfiltration of oil-water emulsion. *Chem Eng J*. 2017;307(643-649. <https://doi.org/https://doi.org/10.1016/j.cej.2016.08.124>

[11] Tiera MJ, Qiu X-P, Bechaouch S, Shi Q, Fernandes JC and Winnik FM. Synthesis and characterization of phosphorylcholine-substituted chitosans soluble in physiological ph conditions. *Biomacromolecules*. 2006;7(11):3151-3156. <https://doi.org/https://doi.org/10.1021/bm060381u>

[12] Dou W, Qi F, Li Y, Wei F, Hu Q, Yao Z, Wang J, Zhang L and Tang Z. Charge-biased nanofibrous membranes with uniform charge distribution and hemocompatibility for enhanced selective adsorption of endotoxin from plasma. *J Membr Sci*. 2023;666(121134. <https://doi.org/https://doi.org/10.1016/j.memsci.2022.121134>

[13] Zhao K, Lin R, Chen W, Li Z, Wu K, Guan B, Jiao Y and Zhou C. Polydopamine-assisted immobilization of l-serine onto pes electrospun fiber membrane for effective endotoxin removal. *Compos Commun*. 2020;20(100365. <https://doi.org/https://doi.org/10.1016/j.coco.2020.100365>

[14] Bao J, Chen S, Liu X, Ling Z, Jiang C, Han Z, Wang W, Wei R, Zhao C and Zhao W. Electrospun groove shaped fibers with excellent hemocompatibility and highly selective capture of live bacteria from blood. *Chem Eng J*. 2025;509(161250. <https://doi.org/https://doi.org/10.1016/j.cej.2025.161250>

[15] Huang Y, Yuan Z, Zhao D, Wang F, Zhang K, Li Y, Wen Y and Wang C. Polymyxin b immobilized nanofiber sponge for endotoxin adsorption. *Eur Polym J*. 2019;110(69-75. <https://doi.org/https://doi.org/10.1016/j.eurpolymj.2018.11.008>

[16] Cai Y, Li Z and Hong Y. Construction of porous bilayer cellulose acetate/silk fibroin membranes for bloodstream infection treatment. *ACS Appl Eng Mater*. 2023;1(6):1655-1668. <https://doi.org/https://doi.org/10.1021/acsaenm.3c00165>

[17] Li Y, Li J, Shi Z, Wang Y, Song X, Wang L, Han M, Du H, He C and Zhao W. Anticoagulant chitosan-kappa-carrageenan composite hydrogel sorbent for simultaneous endotoxin and bacteria cleansing in septic blood. *Carbohydr Polym*. 2020;243(116470. <https://doi.org/https://doi.org/10.1016/j.carbpol.2020.116470>

[18] Dang Q, Li C-G, Jin X-X, Zhao Y-J and Wang X. Heparin as a molecular spacer immobilized on microspheres to improve blood compatibility in hemoperfusion. *Carbohydr Polym*. 2019;205(89-97. <https://doi.org/https://doi.org/10.1016/j.carbpol.2018.08.067>

[19] Zong W, Chen J, Han W, Cheng G, Chen J, Wang Y, Wang W, Ou L, Yu Y and Shen J. Preparation of pva/amino multi-walled carbon nanotubes nanocomposite microspheres for endotoxin adsorption. *Artif Cells, Nanomed, Biotechnol*. 2018;46(1):185-191. <https://doi.org/https://doi.org/10.1080/21691401.2017.1304405>

[20] Wei Z, Fan Z, Peng G, Si H, Yang L, Wang Z, Yin S, Chen S, Wang R and Xie Y. Extracorporeal hemoperfusion therapy for sepsis: Multi-lamellar microspheres towards cascade endotoxin removal and broad-spectrum radical eliminating. *Chem Eng J*. 2022;444(136499. <https://doi.org/https://doi.org/10.1016/j.cej.2022.136499>

[21] Yang B, Liu X, Zhao W, Wei R and Zhao C. Hemocompatible polyethylenimine-grafted poly (glycidyl methacrylate) based microspheres for effective endotoxin removal. *Polymer*. 2024;296(126839. <https://doi.org/https://doi.org/10.1016/j.polymer.2024.126839>

[22] Ling Z, Jiang C, Liu X, Li Y, Zhao W and Zhao C. Histidine-inspired polyacrylonitrile-based adsorbent with excellent hemocompatibility for the simultaneous removal of bacteria and endotoxin in septic blood. *Compos Part B-Eng*. 2023;266(110994. <https://doi.org/https://doi.org/10.1016/j.compositesb.2023.110994>

[23] Im P, Jeong HG, Kim D, Song J, Phan NM, Choi ES, Kim YM, Jeong SH, Park J and Park I. Extracorporeal catalytic hemoperfusion therapy for refractory septic shock using ceria nanoparticle‐embedded porous microbeads. *Adv Funct Mater*. 2025;2415949. <https://doi.org/https://doi.org/10.1002/adfm.202415949>
